# Assembling draft genomes using contiBAIT

**DOI:** 10.1093/bioinformatics/btx281

**Published:** 2017-05-05

**Authors:** Kieran O’Neill, Mark Hills, Mike Gottlieb, Matthew Borkowski, Aly Karsan, Peter M Lansdorp

**Affiliations:** 1 Michael Smith Genome Sciences Centre, BC Cancer Agency, University of British Columbia, Vancouver, BC, Canada; 2 Department of Pathology and Laboratory Medicine, Faculty of Medicine, University of British Columbia, Vancouver, BC, Canada; 3 Terry Fox Laboratory, BC Cancer Agency, University of British Columbia, Vancouver, BC, Canada; 4 Bioinformatics Training Program, Faculty of Medicine, University of British Columbia, Vancouver, BC, Canada; 5 Independent Researcher, University of British Columbia, Vancouver, BC, Canada; 6 Hematology Division, Faculty of Medicine, University of British Columbia, Vancouver, BC, Canada; 7 European Research Institute for the Biology of Ageing, University Medical Centre Groningen, Groningen, The Netherlands

## Abstract

**Summary:**

Massively parallel sequencing is now widely used, but data interpretation is only as good as the reference assembly to which it is aligned. While the number of reference assemblies has rapidly expanded, most of these remain at intermediate stages of completion, either as scaffold builds, or as chromosome builds (consisting of correctly ordered, but not necessarily correctly oriented scaffolds separated by gaps). Completion of *de novo* assemblies remains difficult, as regions that are repetitive or hard to sequence prevent the accumulation of larger scaffolds, and create errors such as misorientations and mislocalizations. Thus, complementary methods for determining the orientation and positioning of fragments are important for finishing assemblies. Strand-seq is a method for determining template strand inheritance in single cells, information that can be used to determine relative genomic distance and orientation between scaffolds, and find errors within them. We present contiBAIT, an R/Bioconductor package which uses Strand-seq data to repair and improve existing assemblies.

**Availability and Implementation:**

contiBAIT is available on Bioconductor. Source files available from GitHub.

**Supplementary information:**

Supplementary data are available at *Bioinformatics* online.

## 1 Introduction

High-quality genome assemblies have revolutionized the analysis of mutations, structural variation, gene expression and evolution. However, many organisms do not have finished assemblies, posing challenges to researchers as regions cannot be located to chromosomes and errors may be construed as biological effects. Genome assembly typically relies on overlapping sequences to increase the size of contiguous sequences (contigs). Biases in genomic representation from hard-to-sequence and repetitive regions can hide overlaps, resulting in assembly gaps that are difficult to span. Therefore, other methods are essential to connect adjacent contigs into larger scaffolds and ultimately help finish assemblies.

We previously developed Strand-seq, a single cell sequencing technique that sequences only template strands (Falconer *et al.*, 2012), allowing us to identify multiple misorientations within the mouse assembly (GRCm37). Using just the contig sequences from the mouse, MGSCv3, we were further able to show that we could cluster contigs into the chromosome to which they belong (Hills *et al.*, 2013). To aid in this task, we developed *Bioinformatics Analysis of Inherited Templates* (BAIT) (Hills *et al.*, 2013). Strand-seq also has the ability to identify sister chromatid exchanges (SCEs) at unprecedented resolution by detecting changes in template strand sequences (Falconer *et al.*, 2012), which we used as a linkage metric to order fragments within each chromosome (Hills *et al.*, 2013).

Here, we present contiBAIT, a multi-platform R/Bioconductor toolkit for genome finishing using Strand-seq. Like BAIT, contiBAIT identifies SCEs in order to cluster fragments into chromosomes and order fragments within chromosomes. contiBAIT extends BAIT’s functionality by detecting misassembled and misoriented contigs within an assembly, while also improving upon BAIT’s accuracy and run time.

## 2 Detection of missassembly

**Fig. 1:**
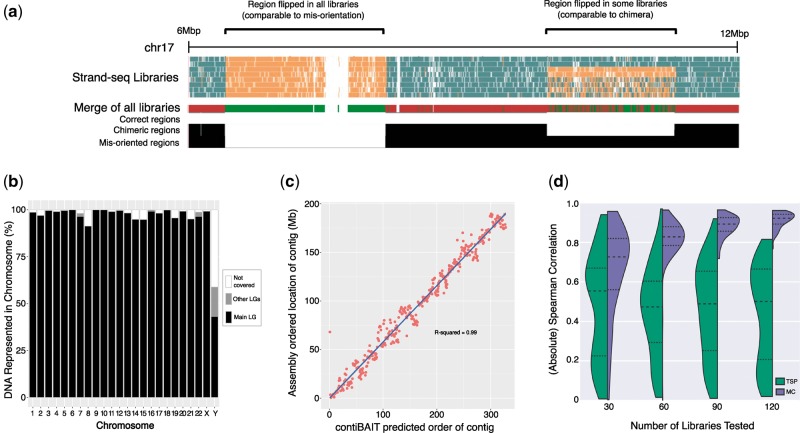
(**a**) Misorientation and chimera detection. Eight libraries with read directions artificially changed to model misorientations and chimeras. contiBAIT merges libraries and correctly identifies features, represented by the black histogram (**b**). Clustering on 120 GRCh38scaf aligned human samples. Proportion of LGs (largest LG in black, subsequent LGs in grey, gaps in white) representing each chromosome (x axis). The majority of chromosomes are represented by only a single LG, and no LG contains substantial numbers of fragments from any but the primary chromosome. (**c**) Ordering by Monte Carlo method on human chromosome 4 aligned to GRCh38scaf. The location predicted by contiBAIT showed high correlation with the actual order of fragments. (**d**) Comparison of TSP and Monte Carlo algorithm on GRCh38scaf chromosome 4, showing distributions and quartiles for sets of 100 random samplings. The Monte Carlo method showed a marked improvement over the TSP formulation, especially with more libraries

Misassemblies can be defined as errors in orientation or localization of sequences. Orientation errors occur when a fragment is correctly placed but has the opposite orientation of its neighbours (although a homozygous polymorphic inversion may have the same appearance). Mislocalizations occur when low level component sequences from non-adjacent locations, such as different chromosomes, are fused together. These errors, respectively, known as misorientations and chimeras, affect the strand-state patterns in detectable ways (see Fig. 1a): Misorientations present as a complementary switch in template strand directionality. Chimeras have independent template strand directionalities on either side of the fusion.

contiBAIT uses a circular binary segmentation algorithm (Olshen *et al.*, 2004) to find these changes in template strands and uses them to reorient or split fragments for downstream clustering. On eight test libraries harbouring an artificial misorientation and chimera, contiBAIT merged similar libraries together, and accurately identified features to within a 1562 bp interval on average (Fig. 1a). These data suggest that contiBAIT is uniquely effective at identifying and correcting errors within a given assembly.

## 3 Clustering fragments into linkage groups

contiBAIT takes a set of unlinked fragments and clusters them into putative chromosomes in two passes. In the first pass, these fragments are clustered based on heterozygous or homozygous strand-state patterns across libraries without taking orientation into consideration. This creates a cluster of fragments for each chromosome, called a linkage group (LG). In the second pass, contiBAIT reorients fragments within each LG so that all are in the same direction.

Genome assemblies may have hundreds of thousands of contigs, which presents a challenge for clustering. This challenge is compounded by the sparsity of Strand-seq data. We were unable to find a clustering method that handles large, sparse, categorical datasets. Consequently, we developed a memory efficient clustering algorithm based on the ‘Chinese restaurant’ process, described in detail in Supplementary Figure S1.

We tested the clustering ability of contiBAIT by aligning 120 human Strand-seq libraries from (Sanders *et al.*, 2016) to an artificial assembly, which we created by splitting the human GRCh38 into 500 kbp ’scaffolds’ (subsequently referred to as GRCh38scaf). contiBAIT clustered 98.5% of the mappable genome into LGs, with 99.5% of that clustering into the largest 24 LGs, representing one LG for each chromosome in the (male) human sample. These LGs were both sensitive and specific for the true chromosomes (Fig. 1b).

Only six fragments (0.1%) misclustered into an LG which they did not belong, while the remaining 5600 fragments clustered correctly. The 0.5% of the assembly that did not cluster into the first 24 LGs represented a heterozygous inversion (on chr7q11.22) present in the human sample relative to the GRCh38 reference, and the pseudoautosomal regions of the sex chromosomes (present on both chrX and chrY, but only represented on chrX in GRCh38) (Sanders *et al.*, 2016).

## 4 Ordering fragments within linkage groups

SCEs within libraries allow fragments to be localized within chromosomes (Hills *et al.*, 2013). In BAIT, we modelled fragment order as a travelling salesman problem (TSP) (Hills *et al.*, 2013). However, this approach did not account for biologically unlikely strand-state changes or the rarity of SCEs per cell, and had NP-hard time complexity. contiBAIT employs a Monte Carlo method to quickly order fragments within each LG, while penalizing excessive SCEs and unlikely strand-state changes (see Supplementary Fig. S2).

We compared the fragment ordering algorithms from BAIT and contiBAIT on 120 libraries aligned to GRCh38scaf. contiBAIT’s predicted order of contigs within chromosomes showed a strong correlation with the actual order of these fragments (Fig. 1c). This correlation was better than using the TSP formulation, even when relatively few libraries were tested (Fig. 1d). Moreover, clustering and ordering of GRCh38scaf was 938× faster for contiBAIT than BAIT (52 versus 48, 735 minutes on a 2.93GHz i7 CPU).

## 5 Conclusion

contiBAIT is a platform-independent analysis tool for finishing assemblies using Strand-seq data. Compared with BAIT, contiBAIT is almost three orders of magnitude faster and more accurate at clustering and ordering fragments into chromosomes. Moreover, contiBAIT has the unique ability to accurately detect misorientations and assembly chimeras within fragments. With these capabilities, contiBAIT is poised to be a valuable addition to the toolkit for constructing and finishing reference genome assemblies, augmenting standard sequencing methodologies and increasing efficiency for directed resequencing efforts.

## Funding

This work was supported by the Canadian Institutes of Health Research [RMF-92093, CGS-M, CIHR BTP fellowship]; U.S. National Institutes of Health [R01GM094146]; Canadian Cancer Society; Michael Smith Foundation for Health Research; Terry Fox Foundation [018006 and 150265]; and Terry Fox Research Institute (TFF-122869).

*Conflict of Interest*: none declared.

## Supplementary Material

Supplementary Information

Supplementary Data
