# Supplementary material for: Assembling draft genomes using contiBAIT
Source: Bioinformatics. 2017 May 5;33(17):2737–9. doi: 10.1093/bioinformatics/btx281 (PMC5860061; doi:10.1093/bioinformatics/btx281)
Supplement: Supplementary Information [file btx281_supplementary_information.pdf]

Supplementary Information

February 28, 2017

Additional Notes

- A more detailed discussion of an earlier version of contiBAIT, along with a comparison to BAIT on a mouse data set, have been presented in chapter 7 of Dr O'Neill's PhD thesis. This document is available at `https://open.library.ubc.ca/cIRcle/collections/ubctheses/24/items/1.0135595`, and the relevant chapter has been included as supplemental information (under the CC-BY license.)
- In Fig 1c, there is a dot at position (0,70). This is a fragment that was incorrectly assigned to this chromosome during the clustering phase. During ordering, it was correctly placed at the end of the chromosome (since it is not actually on this chromosome).

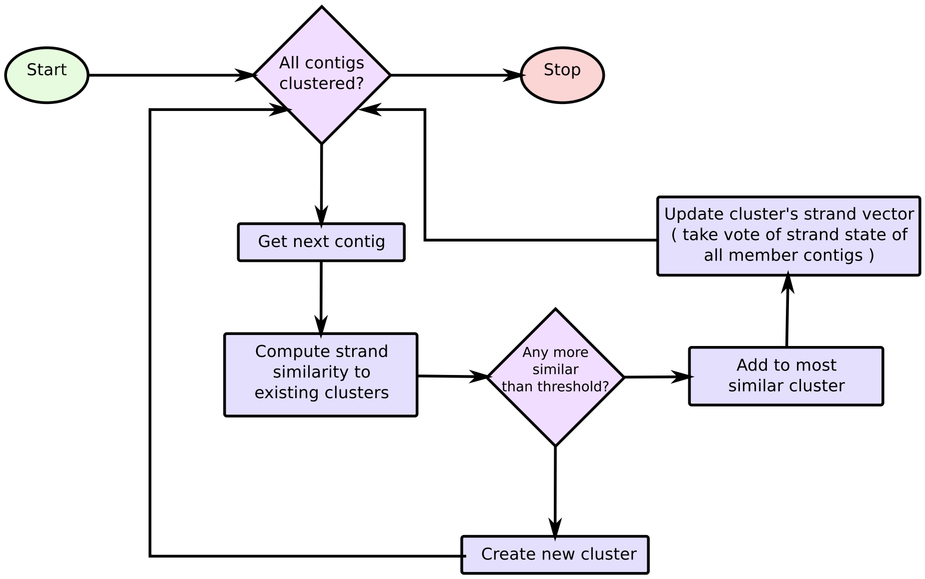

Figure 1: **Flow diagram illustrating three-star Chinese restaurant clustering.** For each contig, the similarity in strand state between the contig and the existing clusters (if any) is computed. If this similarity exceeds a pre-defined threshold for any of the existing clusters, the contig is assigned to the most similar cluster. If no clusters are similar enough, a new cluster is created. For the purposes of computing similarity, a consensus strand state is computed for each cluster by a simple vote of the strand states of its members.

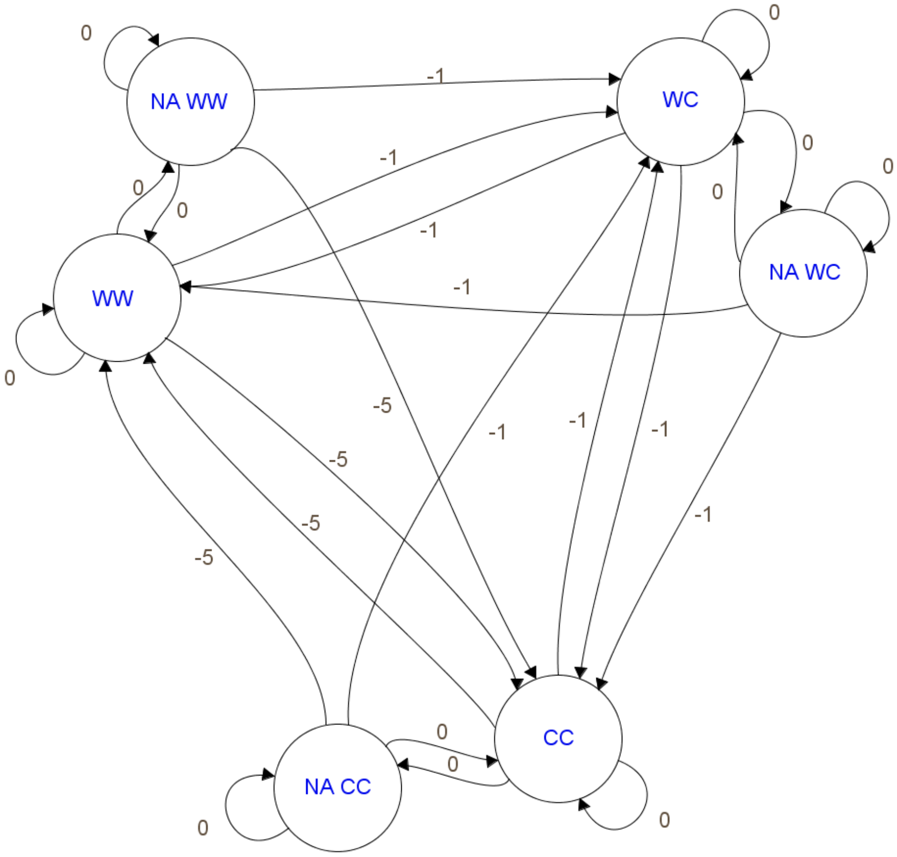

Figure 2: Scoring function for the Monte Carlo ordering algorithm. WW-CC transitions are assigned a -5 penalty. Other changes in strand state receive a -1 penalty (to minimise apparent SCEs). Missing strand states (NA) are accounted for by remembering the state before.
