# Supplementary material for: Assembling draft genomes using contiBAIT
Source: Bioinformatics. 2017 May 5;33(17):2737–9. doi: 10.1093/bioinformatics/btx281 (PMC5860061; doi:10.1093/bioinformatics/btx281)
Supplement: Supplementary Data [file btx281_supplementary_thesis_chaper_7.pdf]

Automated Analysis of Single Cell Leukemia Data

by

Kieran O'Neill

B.Sc. University of Natal, 2002
B.Sc. (Hons) University of KwaZulu-Natal, 2004
M.Sc. University of KwaZulu-Natal, 2008

A THESIS SUBMITTED IN PARTIAL FULFILLMENT OF
THE REQUIREMENTS FOR THE DEGREE OF

DOCTOR OF PHILOSOPHY

in

The Faculty of Graduate and Postdoctoral Studies

(Bioinformatics)

THE UNIVERSITY OF BRITISH COLUMBIA

(Vancouver)

July 2014

© Kieran O'Neill 2014

Abstract

Acute myeloid leukemia (AML) is a high grade malignancy of non-lymphoid cells of the hematopoietic system. AML is a heterogeneous disease, and numerous attempts have been made to risk-stratify AML so that appropriate treatment can be offered. Single cell analysis methods could provide insights into the biology of AML leading to risk-stratified and functionally tailored treatments and hence improved outcomes. Recent advances in flow cytometry allow the simultaneous measurement of up to 17 antibody markers per cell for up to millions of cells, and it is performed routinely during AML clinical workup. However, despite vast amounts of flow cytometry data being gathered, comprehensive, objective and automated studies of this data have not been undertaken. Another method, strand-seq, elucidates template strand inheritance in single cells, with a range of potential applications, none of which had been automated when this thesis work commenced. I have developed bioinformatic methods enabling research into AML using both these types of data.

I present flowBin, a method for faithfully recombining multitube flow cytometry data. I present flowType-DP, a new version of flowType, able to process flow cytometry and other single cell data having more than 12 markers (including flowBin output). I demonstrate the application of flowBin to AML data, for digitally isolating abnormal cells, and classifying AML patients. I also use flowBin in conjunction with flowType to find cell types associated with clinically relevant gene mutations in AML.

I present BAIT, a software package for accurately detecting sister chromatid exchanges in strand-seq data. I present functionality to place unbridged contigs in late-build genomes into their correct location, and have, with collaborators, published the corrected locations of more than half the unplaced contigs in the current build of the mouse genome. I present contiBAIT, a software package for assembling early-build genomes which consist entirely of unanchored, unbridged contigs. ContiBAIT has the potential to dramatically improve the quality of many model organism genomes at low cost.

These developments enable rapid, automated, objective and reproducible deep profiling of AML flow cytometry data, subclonal cell analysis of AML cytogenetics, and improvements to model organisms used in AML research.

Preface

Attribution of Work and Published Materials

Chapter 2 (Background)

In chapter 2, the section* on flow cytometry bioinformatics incorporates material from a review article which I co-authored for PLoS Computational Biology [1]. I wrote roughly half of the original article, while much of the remainder was written by my co-first author, Nima Aghaeepour. Josef Ŝpidlen provided one section*, while Ryan Brinkman provided comments and oversight. The remainder of the chapter is my own, original work.

The full citation is:
K. O'Neill, N. Aghaeepour, J. Špidlen, and R. Brinkman, "Flow Cytometry Bioinformatics," *PLoS Comput Biol*, vol. 9, p. e1003365, Dec. 2013

Chapter 3 (Enhanced FlowType)

Chapter 3 details substantial improvements made especially to the flowType and also to the RchyOptimyx Bioconductor packages. This work was published in Bioinformatics as an application note, [2] and the chapter incorporates text from that article. FlowType was originally created by Nima Aghaeepour [3]. RchyOptimyx was originally created by Nima and Adrin Jalali, with some input from myself [4]. Adrin and I jointly developed the core algorithm of the new flowType, and collaborated to implement it in C++. I incorporated the C++ code into the flowType package, performed the performance assessment work (besides Nima's contribution), and wrote the article. Adrin made the minor changes necessary in RchyOptimyx. Nima did the initial run of the time performance assessment of the new flowType, as well as updating documentation for the two packages. The remaining authors contributed to the conception and writing.

The full citation is:
K. O'Neill, A. Jalali, N. Aghaeepour, H. Hoos, and R. R. Brinkman, "Enhanced flowType-/RchyOptimyx: A Bioconductor pipeline for discovery in high-dimensional cytometry data," *Bioinformatics*, vol. 30, pp. 1329–30, Jan. 2014

Chapters 4 and 5 (FlowBin)

Although not yet published, the contents of Chapters 4 and 5 describing the design, validation and applications of flowBin, are entirely my own work, with the following exceptions: Donna Hogge provided half of the *NPM1* genotype data, while Aly Karsan

provided the other half. The whole-genome AML mutation data was provided by Linda Chang, Gerben Duns, Jeremy Parker and Aly Karsan. Bakul Dalal provided the flow cytometry data, as well as scientific guidance. Nima Aghaeepour and Ryan Brinkman provided scientific guidance.

The second section* in Chapter 5 describes work I undertook as an entry to the Flow Cytometry: Critical Assessment of Population Identification Methods (FlowCAP) competition, which was ultimately published in 2013.[5] I have incorporated some text which I authored from the supplementary material of that paper; Nature Publishing Group does not assert any copyright or licensing on supplementary material and so I have full rights to re-use it here without explicit permission. The remainder of the work described in that section* was undertaken by myself, with scientific guidance from Nima Aghaeepour, Habil Zare and Ryan Brinkman.

The full citation is:
N. Aghaeepour, G. Finak, T. F. Consortium, T. D. Consortium, H. Hoos, T. R. Mosmann, R. Brinkman, R. Gottardo, and R. H. Scheuermann, "Critical assessment of automated flow cytometry data analysis techniques," *Nature Methods*, vol. 10, pp. 445–445, May 2013

Chapters 6 (BAIT) and 7 (ContiBAIT)

Chapter 6 and a small part of Chapter 7 incorporate text from an article published in Genome Medicine on which I was second author, and which represented a collaborative work primarily between myself and Mark Hills, the first author. [6] Mark developed much of the framework code for the BAIT package we described in that article, and wrote most of the article. Although I contributed extensively to the design of the evaluation work presented in the results section* of that paper, the work itself was performed by Mark. However, the more technical aspects of BAIT were mostly my work, including the core of the sister chromatid exchange prediction algorithm, the algorithm for placing unbridged contigs in late-build genomes, and the algorithm for clustering contigs into putative chromosomal linkage groups in early-build genomes. I also contributed extensively to the writing of the methodology section* of the article. The remaining authors – Ester Falconer, Ryan Brinkman and Peter Lansdorp, provided guidance for the project and writing. As such, I have incorporated text and figures from that article pertaining to those parts of the methodology to which I was the main contributor, as well as those parts of the results section*s, where my work was validated.

The parts of Chapter 7 not taken from the BAIT paper are my own work, with the exception that Mark Hills performed the initial alignments of read data, and generated the . The remaining algorithmic design, implementation and validation was all my own work, with substantial planning input from Mark.

The full citation is:
M. Hills, K. O'Neill, E. Falconer, R. Brinkman, and P. M. Lansdorp, "BAIT: Organizing genomes and mapping rearrangements in single cells," *Genome Medicine*, vol. 5, p. 82, Sept. 2013

Ethics approval

As portions of this work were performed on retrospective human clinical diagnostic data, approval was obtained from the University of British Columbia / British Columbia Cancer Agency Research Ethics Board (certificate number H08-00667).

Table of Contents

Abstract . . . ii

Preface . . . iii

Table of Contents . . . vi

List of Tables . . . x

List of Figures . . . xi

List of Abbreviations . . . xiii

Acknowledgments . . . xiv

Dedication . . . xvi

1 Introduction and Problem Statement . . . 1
- 1.1 Problem Statement . . . 2
- 1.2 Solutions . . . 2

2 Background . . . 4
- 2.1 Flow Cytometry Bioinformatics . . . 4
  - 2.1.1 Flow Cytometry Data . . . 4
  - 2.1.2 Steps in Computational Flow Cytometry Data Analysis . . . 6
  - 2.1.3 Data Pre-processing . . . 7
  - 2.1.4 Identifying Cell Populations . . . 9
  - 2.1.5 Diagnosis and Discovery . . . 15
- 2.2 Acute Myeloid Leukemia . . . 18
  - 2.2.1 Leukemia Subtypes and Heterogeneity . . . 19
  - 2.2.2 Flow Cytometry Immunophenotyping . . . 21
  - 2.2.3 Multitube Flow Cytometry Data . . . 22
  - 2.2.4 Bioinformatic Approaches for Combining Multitube Data . . . 26
  - 2.2.5 Surveying the Immunophenotypic Landscape of Leukemia . . . 27
- 2.3 Strand-seq . . . 28
  - 2.3.1 Detection of Sister Chromatid Exchange Events . . . 28
  - 2.3.2 Applications of Strand-seq to Leukemia . . . 30
  - 2.3.3 Automated Analysis of Strand-seq Data . . . 31

3 Enhanced FlowType for High-Dimensional Single Cell Data 32
3.1 Introduction 32
3.2 Methodology 33
3.2.1 Dynamic Programming Implementation 33
3.2.2 Breadth-First Strategy 34
3.2.3 Partitioning into Multiple Expression Levels 35
3.3 Evaluation 36
3.3.1 Performance 36
3.3.2 Memory Limits 36
3.3.3 Example Case: Multiple Expression Levels 37
3.4 Discussion 38

4 FlowBin: Deep Profiling of Multitube Flow Cytometry Data 39
4.1 Introduction 39
4.2 Design and Implementation 41
4.2.1 Population Marker Normalization 42
4.2.2 Binning of Population Markers 42
4.2.3 Bin Matching Across Tubes 43
4.2.4 Expression Measurement 43
4.2.5 Downstream Analysis 43
4.3 Methods 44
4.3.1 Validation of Quantile Normalization 44
4.3.2 Comparison of Binning Methods 44
4.3.3 Comparison to Per-cell Nearest-neighbours 44
4.3.4 Validation on Simulated Multitube Data from Polychromatic Flow Cytometry 45
4.4 Results 45
4.4.1 Validation of Quantile Normalization 45
4.4.2 Comparison of Binning Methods 46
4.4.3 Comparison to Per-cell Nearest-neighbours 47
4.4.4 Validation on Simulated Multitube Data from Polychromatic Flow Cytometry 48
4.5 Discussion 51
4.5.1 Validation of Quantile Normalization 51
4.5.2 Comparison of Binning Methods 51
4.5.3 Comparison to Nearest Neighbours 51
4.5.4 Validation on Simulated Multitube Data from Polychromatic Flow Cytometry 52
4.5.5 Conclusions 53

5 Applications of FlowBin to AML 55
5.1 Introduction 55
5.1.1 Separation of AML and Normal Cells 55
5.1.2 FlowCAP2 55
5.1.3 FlowBin-FlowType to find *NPM1*-associated cell types 56
5.1.4 Surveying the Immunophenotypic and Genomic Landscape of AML 56

5.2 Methods . . . 57
5.2.1 Separation of AML and Normal Cells . . . 57
5.2.2 FlowCAP2 . . . 57
5.2.3 FlowBin-FlowType to find *NPM1*-associated cell types . . . 58
5.2.4 Surveying the Immunophenotypic and Genomic Landscape of AML 60
5.3 Results . . . 61
5.3.1 Separation of AML and Normal Cells . . . 61
5.3.2 FlowCAP2 . . . 62
5.3.3 FlowBin-FlowType to find *NPM1*-associated cell types . . . 62
5.3.4 Surveying the Immunophenotypic and Genomic Landscape of AML 62
5.4 Discussion . . . 66
5.4.1 Separation of AML and Normal Cells . . . 66
5.4.2 Identifying AML Patients (FlowCAP 2) . . . 68
5.4.3 FlowBin-FlowType to find *NPM1*-associated cell types . . . 68
5.4.4 Surveying the Immunophenotypic and Genomic Landscape of AML 69

6 BAIT: Automated Strand-Seq Analysis . . . 70
6.1 Background . . . 70
6.2 Methodology . . . 71
6.2.1 Automatically Detecting Sister Chromatid Exchanges . . . 71
6.2.2 Placing Contigs in Late-build Genomes Using Strand Inheritance and Sister Chromatid Exchange Information . . . 72
6.3 Results . . . 77
6.3.1 Accurate Localization and Mapping of SCEs . . . 77
6.3.2 Placing Contigs in Late-build Genomes Using Strand Inheritance and Sister Chromatid Exchange Information . . . 79
6.4 Discussion . . . 82

7 ContiBAIT: Assembling Genomes Using strand-seq . . . 84
7.1 Background . . . 84
7.2 Methodology . . . 85
7.2.1 Preprocessing and Calling Template Strand State . . . 85
7.2.2 Anchoring Unbridged Contigs . . . 88
7.2.3 Ordering of Unbridged Contigs within Chromosomes . . . 92
7.3 Results and Validation . . . 93
7.3.1 Contig Anchoring . . . 93
7.3.2 Validation of Contig Ordering . . . 98
7.4 Package . . . 100
7.5 Discussion . . . 100

8 Conclusions and Future Work . . . 102
8.1 Summary of Contributions . . . 102
8.2 Reiteration of Problem Statement . . . 103
8.3 Future Directions . . . 105
8.3.1 FlowType-DP . . . 105
8.3.2 FlowBin . . . 105

8.3.3 Applications of FlowBin to AML 106
8.3.4 BAIT 107
8.3.5 contiBAIT 107

Bibliography 109

List of Tables

2.1 Clinical features and risk stratification of AML 19
2.2 Recurrent non-karyotypic genetic aberrations in AML 20
2.3 Markers commonly assayed in leukemia diagnosis 23

4.1 Markers in simulated multitube data. 46

5.1 Mutations and mutation groups correlated with immunophenotype clusters. 66

6.1 Identification of all misoriented fragments in GRCm38/mm10. 80

List of Figures

2.1 Schematic diagram of a flow cytometer 5
2.2 An example pipeline for analysis of FCM data and some of the Bioconductor packages relevant to each step. 6
2.3 Comparison of consensus of manual gates and automated gates 10
2.4 Cell populations in a high-dimensional mass-cytometry dataset manually gated after dimension reduction using 2D layout for a minimum spanning tree 12
2.5 An example of frequency difference gating 14
2.6 Overview of the flowType/RchyOptimyx pipeline for identification of correlates of protection against HIV 16
2.7 Hematopoeitic markers detected by flow cytometry for leukemia diagnosis. 22
2.8 Multitube flow cytometry data. 24
2.9 Manual analysis of multitube flow data for leukemia immunophenotyping. 25
2.10 Gating leukemic blasts in CD45/SSC: ideal vs reality. 26
2.11 Overview of strand-seq protocol. 29

3.1 Run time comparison of flowType-DP to flowType-BF 36
3.2 Possible thresholds for marker combinations using flowType-DP 37
3.3 Three/four-partition flowType-generated, RchyOptimyx-visualized cell type hierarchy 38

4.1 Overview of the flowBin pipeline, applied to one multitube sample. . . . 41
4.2 One, two and three-dimensional representations of quantile normalization of population markers. 47
4.3 The two options for binning within flowBin: k-means and flowFP, as applied to a 7-tube sample. 48
4.4 Comparison between nearest-neighbours merging and flowBin for two tubes computationally sampled from a real data set. 49
4.5 Performance on flowBin in reproducing a high-colour flow cytometry analysis on simulated flow cytometry data. 50

5.1 Schema for a voting classifier using flowBin output. 57
5.2 Pipeline used to determine *NPM1*-associated immunophenotypes in AML. 58
5.3 An example of RchyOptimyx analysis of one cluster of cell types. . . . 59
5.4 nu-SVM separation of normal and abnormal cell populations in AML samples. 61
5.5 Schema for a voting classifier for flowBin output incorporating balanced bagging. 63

5.6 Overview of cell types which showed significant differences in abundance between *NPM1-mt* and *NPM1-wt*. 64
5.7 Selected classes of cell types showing significant differences in abundance between *NPM1-mt* and *NPM1-wt*. 65
5.8 Top phenotypes and the results of NMF clustering. 67

6.1 Automated identification of sister chromatid exchange (SCE) from strand-seq data. 73
6.2 Optimization of the sister chromatid exchange (SCE) interval detector function. 74
6.3 BAIT localizes unplaced scaffolds in late-version assemblies. 76
6.4 Accuracy of automated sister chromatid exchange (SCE) detection by Bioinformatic Analysis of Inherited Templates (BAIT). 78
6.5 Validation of using strand-seq to map unplaced scaffolds to built genomes. 81

7.1 True ordering of semi-synthetic mouse data for chromosome 3. 86
7.2 Overall ContiBAIT Linkage Group Assignment Pipeline 87
7.3 Flow diagram illustrating three-star Chinese restaurant clustering 89
7.4 Using SCEs to order contigs within a chromosome. 94
7.5 Clustering contigs into linkage groups for early-assembly genomes. 95
7.6 Contig locations in Bioinformatic Analysis of Inherited Templates (BAIT)-compiled linkage groups. 96
7.7 Evaluation of contiBAIT chromosome assignment with different-sized cluster ensembles and different-sized contigs. 98
7.8 A chromosome assignment result after reorientation and merging. 99
7.9 Testing of ordering of contigs on synthetic data 100

List of Abbreviations

CEBPA	CCAAT/enhancer binding protein-alpha. xii, 19
FLT3	Fms-like tyrosine kinase 3. xii, 21
NPM1	nucleophosmin. xii, 19, 21, 62, 66
AML	acute myeloid leukemia. xii, 1, 2, 18, 19, 21, 27, 55–59, 61, 66, 68, 103
BAIT	bioinformatic analysis of inherited templates. xii, 71, 82, 83
BrdU	bromodeoxyuridine. xii, 28
FISH	fluorescence in-situ hybridisation. xii, 31
FlowCAP	Flow Cytometry: Critical Assessment of Population Identification Methods. iv, xii
flowType-BF	flowType brute force. xii, 33
flowType-DP	flowType dynamic programming. xii, 33
FSC	forward scattered light. xii
MDS	myelodysplastic syndrome. xii
MFI	median fluorescent intensity. xii, 43, 52
NK	normal karyotype. xii, 18
SCE	sister chromatid exchange. xii, 30, 70, 71
SSC	side scattered light. xii, 22
SVM	support vector machine. xii, 57, 66
TCGA	the cancer genome atlas. xii, 21

Acknowledgments

The (United States) National Institutes of Health and the Terry Fox Research Initiative generously funded this research.

My supervisor Ryan Brinkman has kept me funded and supported, provided an example in the ways of academic politics, guided me scientifically, and provided extensive advice in the ways of academic writing and presentation. My thesis committee have been a source of sage advice, as well as fair and honest criticism. Paul Pavlidis, Jenny Bryan, Bakul Dalal and Peter Lansdorp, thank-you.

Many administrators have been an enormous support in my thesis work, regularly going above and beyond the call of duty for my sake: Sharon Ruschkowski, Alice Chau, Kristie Baas, Maryam Rahnama, Christine Kelly, Shera Paterson and Cynthia Wong, thank-you all. Most especially, thank-you to Sharon Ruschkowski for swinging me the technical advisor gig on Rise of the Planet of the Apes. Although, in the end, they gutted most of the science and turned it into an action film, the experience was amazing and the money much-needed at the time.

The Brinkman lab provided a collegial, stimulating and fun environment in which this thesis developed. Nima Aghaeepour, Adrin Jalali, Habil Zare, Ali Bashashati, Josef Špidlen, Mélanie Courtot, Adrián Cortés, Parisa Shooshtari, Alireza Khodabakhshi, Radina Droumeva, Mehrnoush Malekesmaeili, Justin Meskas, Marjan Farahbod, and anyone I may have left out, thank-you.

Many collaborators helped with the work that became this thesis, all of whom have been a pleasure to work with: Bakul Dalal, Donna Hogge, Peter Landsdorp, Holger Hoos, Ester Falconer, Aly Karsan, Mark Kirkpatrick, Jeremy Parker, Linda Chang, Gerben Duns, and most especially Adrin Jalali, Nima Aghaeepour and Mark Hills, thank-you all.

The late-PhD Bioinformatics tea house/lunch meetup group has been an invaluable source of friendship, feedback and scientific engagement. Nima Aghaeepour, Adrin Jalali, Rodrigo Goya, Gavin Ha and Andrew McPherson, thank-you and good luck in your respective careers. The rest of the bioinformatics students at UBC and SFU, as well as the students and post-docs at the Terry Fox Laboratory, over the years of hanging out at various socials and conferences, have been both excellent and scientifically stimulating company.

All of my fellow organisers, volunteers and coordinators in GraSPoDS, the AMS Bike Co-op, the organising committee of the Canadian Students Computational Biology Conference 2009, and VanBUG have helped me find a sense of accomplishment beyond that of my scientific work, and gain a wealth of useful skills a PhD alone would never have given me.

John Michael Greer has inspired me in many ways, but most importantly for this thesis, to drastically improve my writing skills.

My family have been enormously understanding and supportive of my decision to move literally to the diametric opposite side of the globe to complete this degree, and have all contrived to come and visit despite the distance. Mom, Dad, Brendan and Rowan, thank-you.

And lastly, but most importantly, my wife Monia, even in the face of the emotional toll of a PhD-bound husband, and the trials and tribulations of moving to Canada, has been steadfast in her love and support. I am quite certain that I owe her, among many other things, my sanity during this process.

Dedication

To Monia, for five years of patience and support.
I love you.

Chapter 1

Introduction and Problem Statement

Acute myeloid leukemia (AML) is a disease with very poor prognosis. Focused research over the past decades has produced significant advances in both prognostic prediction and the understanding of the biology of AML, but little improvement in patient outcomes. New tools and methods are needed to further understand this disease, and from that understanding to develop new and more effective treatments tailored to the risk and biology of individual patients' diseases. In particular, single cell techniques for investigating the clonal and somatic structure of the disease have the potential to increase understanding of the functions of driver mutations, the acquisition of drug resistance, frequency of relapse, relapse-free survival and overall survival. However, appropriate bioinformatic tools are needed to process the large amounts of data these techniques generate.

One potential source of new insight into AML lies in the untapped potential of flow cytometry data. Flow cytometry provides single cell immunophenotypic information for thousands to millions of cells from a single sample [7, 8]. It is performed routinely as part of leukemia clinical diagnosis. However, the analysis used by pathologists is typically manual and low-throughput, focusing only on known patterns. Nevertheless, vast amounts of data are stored in the process, which could be used for deeper retrospective analysis. Chapter 2 provides more detail about flow cytometry as it is applied to leukemia, and about existing bioinformatics methods which can be used for analyzing flow cytometry data.

Another single cell technology with applications to AML research is strand-seq, which allows for determination of single cell template strand inheritance patterns. This information can be used for a range of purposes, from detected genomic rearrangements to assembling genomes. More detail about the strand-seq method and the need for automated analysis tools to complement it may be found in Chapter 2.

1.1 Problem Statement

The problem which this thesis attempted to solve can be summarized:

Although the high-throughput methods of flow cytometry and strand-seq have produced large quantities of data that may facilitate life-saving research into AML treatment, the bioinformatics tools to analyze this data have been limited. Prior to this thesis work, tools for recombining the multitube flow cytometry data typical in AML diagnosis were few and produced imputation errors, while existing downstream methods were poorly suited for the high dimensionality of the resulting data. Tools for automatically analysing strand-seq were non-existent.

1.2 Solutions

Chapter 4 describes a pipeline I developed which combines multi-tube flow cytometry data in a way which minimizes spurious marker combinations, and enables data mining on retrospective multitube flow cytometry data. Chapter 3 describes enhancements I made, in collaboration with Nima Aghaeepour and Adrin Jalali, to the flowType algorithm which enable it to be used on high-dimensional data, such as that produced by flowBin. Chapter 5 details analyses I have performed using flowBin and in some cases flowType, on AML flow cytometry data, and the biological results those have produced.

Chapter 6 describes contributions which I made to BAIT, a software package developed by myself and Mark Hills for automated analysis of strand-Seq data, especially for the purposes of detecting sister chromatic exchanges and placing unmapped contigs

in late-build genomes. Chapter 7 details contiBAIT, a subsequent package which uses strand-seq to assemble early-build genomes from unbridged contigs.

Lastly, Chapter 8 summarizes the contributions I have made, and suggests future work.

Chapter 2

Background

2.1 Flow Cytometry Bioinformatics

2.1.1 Flow Cytometry Data

Flow cytometers operate by hydrodynamically focusing suspended cells so that they separate from each other within a fluid stream. The stream is interrogated by one or more lasers, and the resulting fluorescent and scattered light is detected by photomultipliers. By using optical filters, particular fluorophores on or within the cells can be quantified by peaks in their emission spectra. These may be endogenous fluorophores such as chlorophyll or transgenic green fluorescent protein, or they may be fluorophores covalently bonded to detection molecules such as antibodies for detecting proteins, or hybridization probes for detecting DNA or RNA.

The ability to quantify these has led to flow cytometry being used in a wide range of applications, including but not limited to:

- Monitoring of CD4 count in HIV[9]
- Diagnosis of various cancers[10] [11]
- Analysis of aquatic microbiomes [12]
- Sperm sorting [13]
- Measuring telomere length[14]

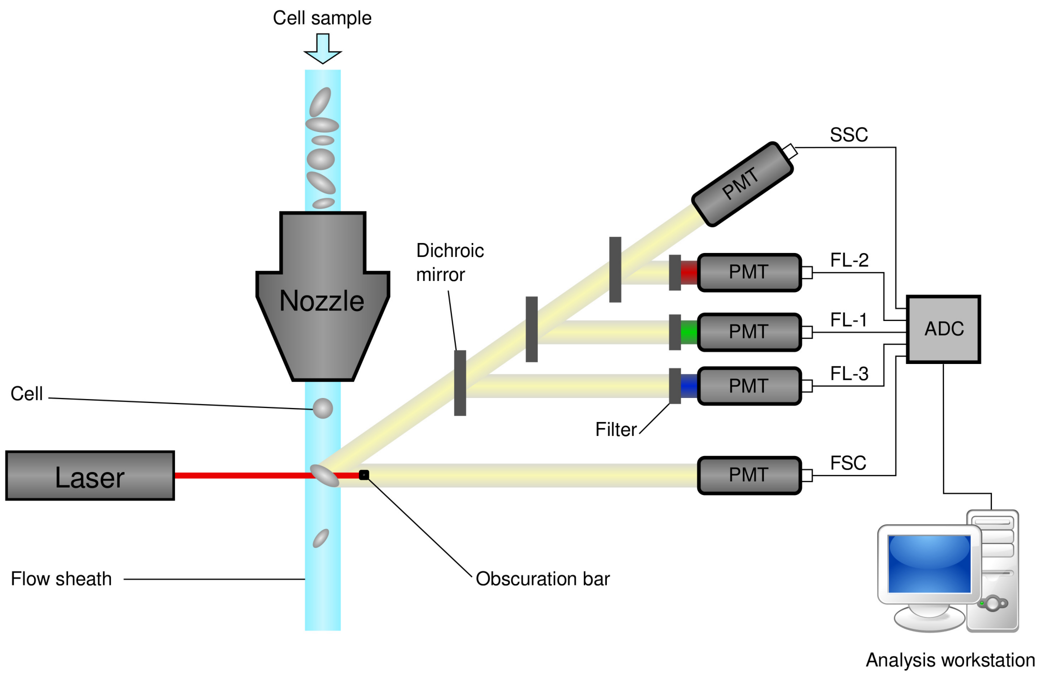

Figure 2.1: Schematic diagram of a flow cytometer, showing focusing of the fluid sheath, laser, optics (in simplified form, omitting focusing), photomultiplier tubes (PMTs), analogue-to-digital converter, and analysis workstation.

Until the early 2000s, flow cytometry could only measure a few fluorescent markers at a time. Through the late 1990s into the mid-2000s, however, rapid development of new fluorophores resulted in modern instruments capable of quantifying up to 18 markers per cell [15]. More recently, the new technology of mass cytometry replaces fluorophores with rare earth elements detected by time of flight mass spectrometry, achieving the ability to measure the expression of 34 or more markers [16]. At the same time, microfluidic real-time polymerase chain reaction (RT-qPCR) methods are providing a flow cytometry-like method of quantifying 48 or more RNA molecules per cell [17]. The rapid increase in the dimensionality of flow cytometry data, coupled with the development of high-throughput robotic platforms capable of assaying hundreds to thousands of samples automatically has created a need for improved computational analysis methods [15].

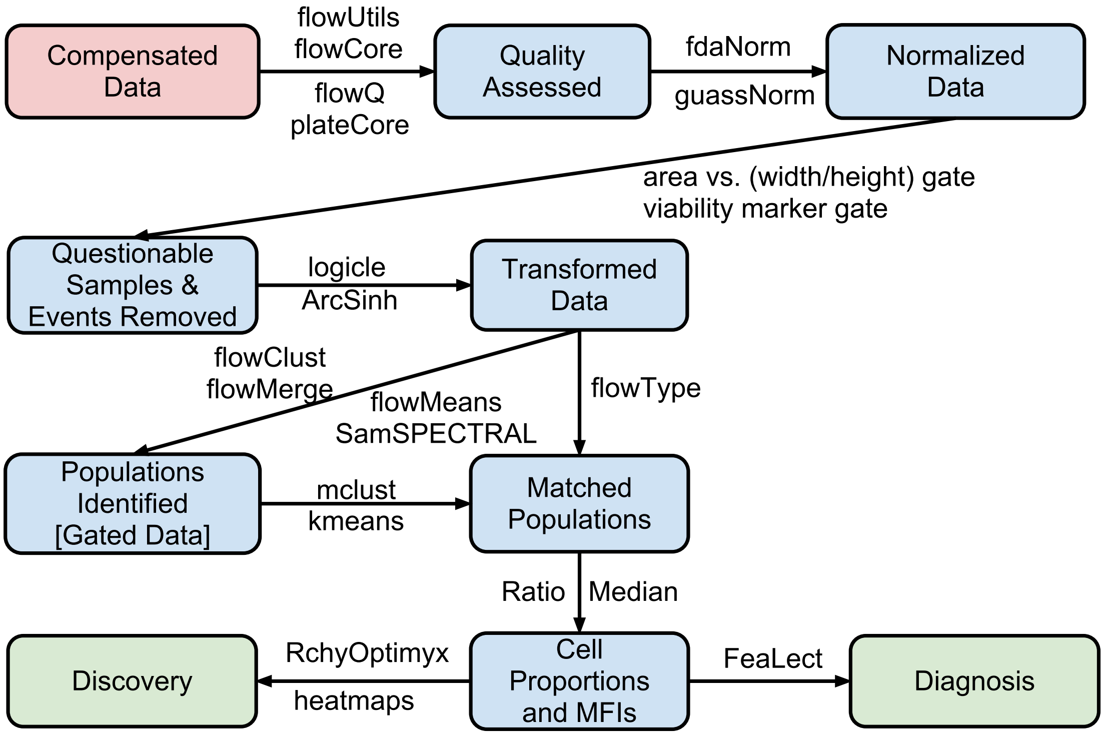

Figure 2.2: An example pipeline for analysis of FCM data and some of the Bioconductor packages relevant to each step.

2.1.2 Steps in Computational Flow Cytometry Data Analysis

The process of moving from primary FCM data to disease diagnosis and biomarker discovery involves four major steps:

1. Data pre-processing (including compensation, transformation and normalization)
2. Cell population identification (a.k.a. gating)
3. Cell population matching for cross sample comparison
4. Relating cell populations to external variables (diagnosis and discovery)

2.1.3 Data Pre-processing

Prior to analysis, flow cytometry data must typically undergo pre-processing to remove artifacts and poor quality data, and to be transformed onto an optimal scale for identifying cell populations of interest. Below are various steps in a typical flow cytometry preprocessing pipeline.

Compensation

When more than one fluorochrome is used with the same laser, their emission spectra frequently overlap. Each particular fluorochrome is typically measured using a bandpass optical filter set to a narrow band at or near the fluorochrome's emission intensity peak. The result is that the reading for any given fluorochrome is actually the sum of that fluorochrome's peak emission intensity, and the intensity of all other fluorochromes' spectra where they overlap with that frequency band. This overlap is termed spillover, and the process of removing spillover from flow cytometry data is called compensation [18].

Compensation is typically accomplished by running a series of representative samples each stained for only one fluorochrome, to give measurements of the contribution of each fluorochrome to each channel [18]. The total signal to remove from each channel can be computed by solving a system of linear equations based on this data to produce a spillover matrix, which when inverted and multiplied with the raw data from the cytometer produces the compensated data [18, 19]. The processes of computing the spillover matrix, or applying a precomputed spillover matrix to compensate flow cytometry data, are standard features of flow cytometry software [20].

Transformation

Cell populations detected by flow cytometry are often described as having approximately log-normal expression [21]. As such, they have traditionally been transformed to a logarithmic scale. In early cytometers, this was often accomplished even before data

acquisition by use of a log amplifier. On modern instruments, data is usually stored in linear form, and transformed digitally prior to analysis.

However, compensated flow cytometry data frequently contains negative values due to compensation, and cell populations do occur which have low means and normal distributions [22]. Logarithmic transformations cannot properly handle negative values, and poorly display normally distributed cell types [22, 23]. Alternative transformations which address this issue include the log-linear hybrid transformations Logicle[24] and Hyperlog [25], as well as the hyperbolic arcsine and the Box-Cox [26].

A comparison of commonly used transformations concluded that the biexponential and Box-Cox transformations, when optimally parameterized, provided the clearest visualization and least variance of cell populations across samples [23]. However, a later comparison of the flowTrans package used in that comparison indicated that it did not parameterize the Logicle transformation in a manner consistent with other implementations, potentially calling those results into question [27]. Based on these results, either the Box-Cox transformation, or Logicle, when correctly paramaterised, are likely to be best choice for transformation.

Quality Control

Particularly in larger flow cytometry data sets, quality control and assessment are important tasks. However, neither the software provided by instrument manufacturers nor most commonly used analysis suites provide such functionality [28]. One solution is to visualize summary statistics, such as the empirical distribution functions of single dimensions of technical or biological replicates to ensure they are the similar [28]. For more rigor, the Kolmogorov–Smirnov test can be used to determine if individual samples deviate from the norm [28]. The Grubbs' test for outliers may be used to detect samples deviating from the group.

A method for quality control in higher-dimensional space is to use probability binning with bins fit to the whole data set pooled together [29]. Then the standard deviation of

the number of cells falling in the bins within each sample can be taken as a measure of multidimensional similarity, with samples that are closer to the norm having a smaller standard deviation [29]. With this method, higher standard deviation can indicate outliers, although this is a relative measure as the absolute value depends partly on the number of bins.

With all of these methods, the cross-sample variation is being measured. However, this is the combination of technical variations introduced by the instruments and handling, and actual biological information that is desired to be measured. Disambiguating the technical and the biological contributions to between-sample variation can be a difficult to impossible task [30].

Normalization

Particularly in multi-centre studies, technical variation can make biologically equivalent populations of cells difficult to match across samples. Normalization methods to remove technical variance, frequently derived from image registration techniques, are thus a critical step in many flow cytometry analyses. Single-marker normalization can be performed using landmark registration, in which peaks in a kernel density estimate of each sample are identified and aligned across samples [30].

2.1.4 Identifying Cell Populations

The complexity of raw flow cytometry data (dozens of measurements for thousands to millions of cells) makes answering questions directly using statistical tests or supervised learning difficult. Thus, a critical step in the analysis of flow cytometric data is to reduce this complexity to something more tractable while establishing common features across samples. This usually involves identifying multidimensional regions that contain functionally and phenotypically homogeneous groups of cells [32]. There are a variety of methods by which this can be achieved, detailed below.

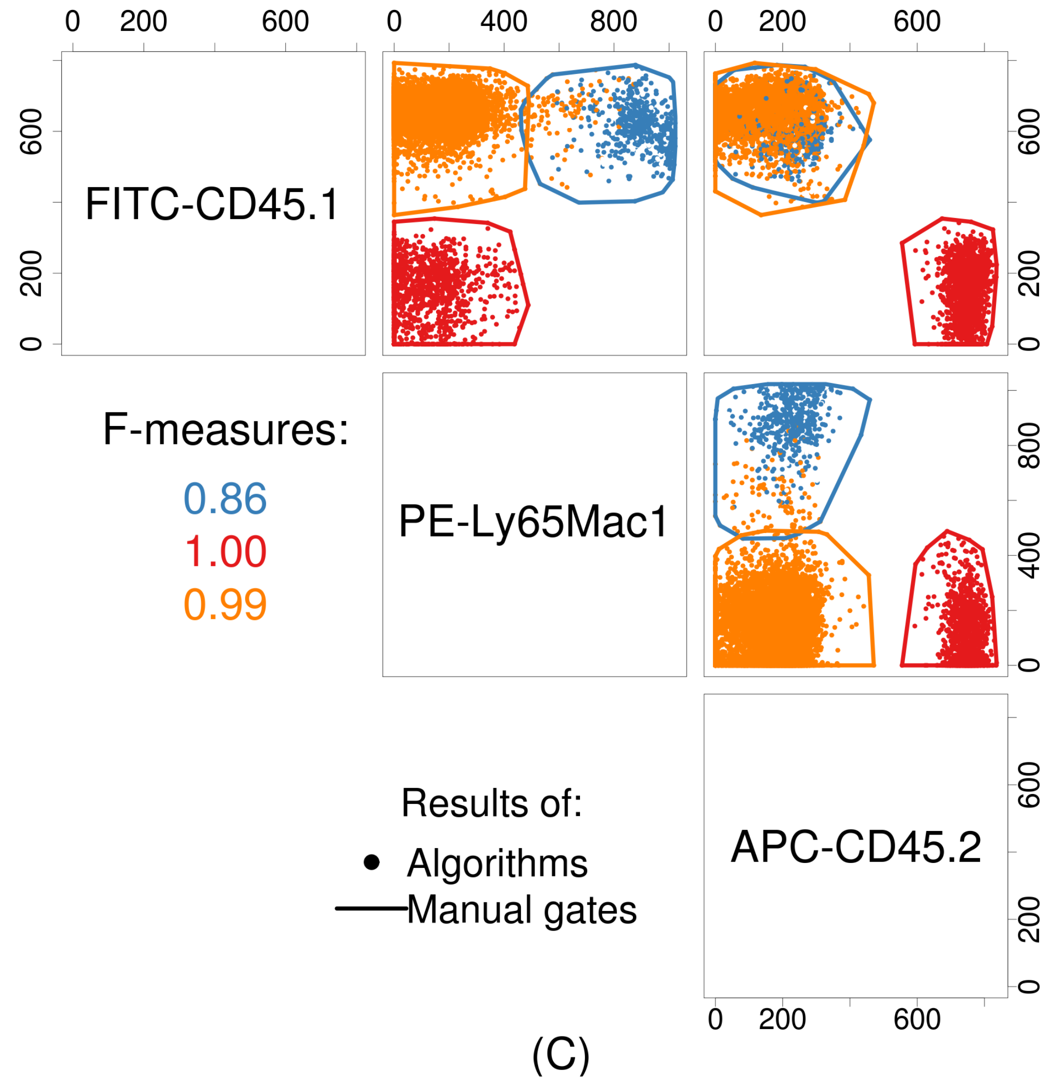

(C)

Figure 2.3: Comparison of consensus of eight independent manual gates (polygons) and automated gates (colored dots). The consensus of the manual gates and the algorithms were produced using the CLUE package [31] Figure ©Nature Publishing Group 2013 [5], re-used under the terms of the CC-BY 3.0 license.

.

Gating

The data generated by flow-cytometers can be plotted in one or two dimensions to produce a histogram or scatter plot. The regions on these plots can be sequentially separated, based on fluorescence intensity, by creating a series of subset extractions, termed "gates". In datasets with a low number of dimensions and limited cross-sample technical and biological variability (e.g., clinical laboratories), manual analysis of specific cell populations can produce effective and reproducible results. However, exploratory analysis of a large number of cell populations in a high-dimensional dataset is not feasible [33]. In addition, manual analysis in less controlled settings (e.g., cross-laboratory studies) can increase the overall error rate of the study [34, 35]. However, despite the considerable advances in computational analysis, manual gating remains the main solution for the identification of cell populations.

Gating Guided by Dimensionality Reduction

The number of scatter plots that need to be investigated increases with the square of the the number of markers measured (or faster since some markers need to be investigated several times for each group of cells to resolve high-dimensional differences between cell types that appear to be similar in most markers) [37] To address this issue, principal component analysis has been used to summarize the high-dimensional datasets using a combination of markers that maximizes the variance of all data points.[38] However, PCA is a linear method and is not able to preserve complex and non-linear relationships. More recently, two dimensional minimum spanning tree layouts have been used to guide the manual gating process. Density-based down-sampling and clustering was used to better represent rare populations and control the time and memory complexity of the minimum spanning tree construction process.[39]. More sophisticated dimension reduction algorithms are yet to be investigated.

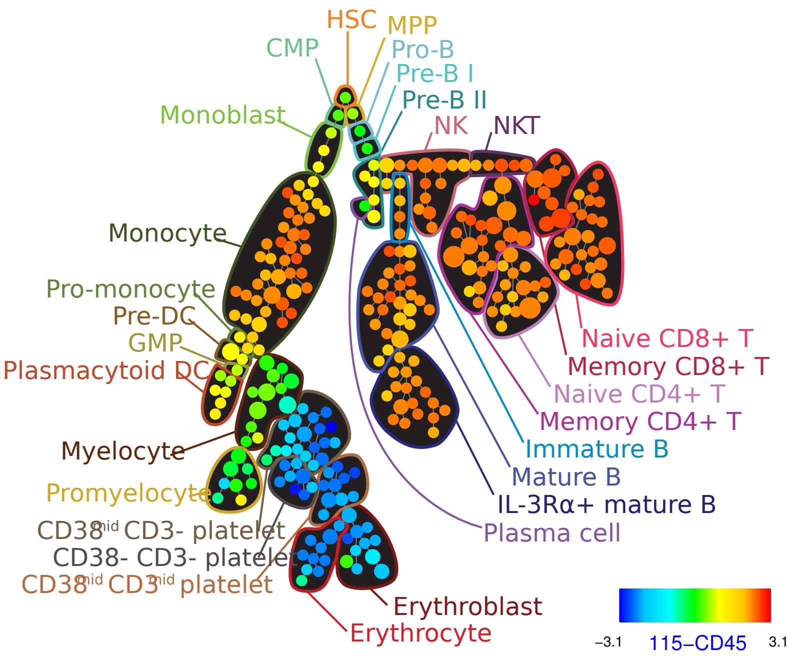

Figure 2.4: Cell populations in a high-dimensional mass-cytometry dataset manually gated after dimension reduction using 2D layout for a minimum spanning tree. Figure reproduced from the data provided in [36]

Automated Gating

Developing computational tools for identification of cell populations has been an area of active research only since 2008. Many individual clustering approaches have recently been developed, including model-based algorithms (e.g., flowClust [26] and FLAME [40]), density based algorithms (e.g. FLOCK [41] and SWIFT, graph-based approaches (e.g. SamSPECTRAL [42]) and most recently, hybrids of several approaches (flowMeans [43] and flowPeaks [44]). These algorithms are different in terms of memory and time complexity, their software requirements, their ability to automatically determine the required number of cell populations, and their sensitivity and specificity. The FlowCAP (Flow Cytometry: Critical Assessment of Population Identification Methods) project, with active participation from most academic groups with research efforts in the area, is providing a way to objectively cross-compare state-of-the-art automated analysis approaches [5].

Probability Binning Methods

Where most automated gating methods attempt to identify coherent cell populations, probability binning is primarily a method for surveying the distribution of cells within a sample, which can also be used for gating. In probability binning, flow cytometry data is split into quantiles on a univariate basis [45]. The locations of the quantiles can then be used to test for differences between samples (in the variables not being split) using the chi-squared test [45].

This was later extended into multiple dimensions in the form of frequency difference gating, a binary space partitioning technique where data is iteratively partitioned along the median [46]. These partitions (or bins) are fit to a control sample. Then the proportion of cells falling within each bin in test samples can be compared to the control sample by the chi squared test.

Finally, cytometric fingerprinting uses a variant of frequency difference gating to set bins and measure for a series of samples how many cells fall within each bin [29]. These

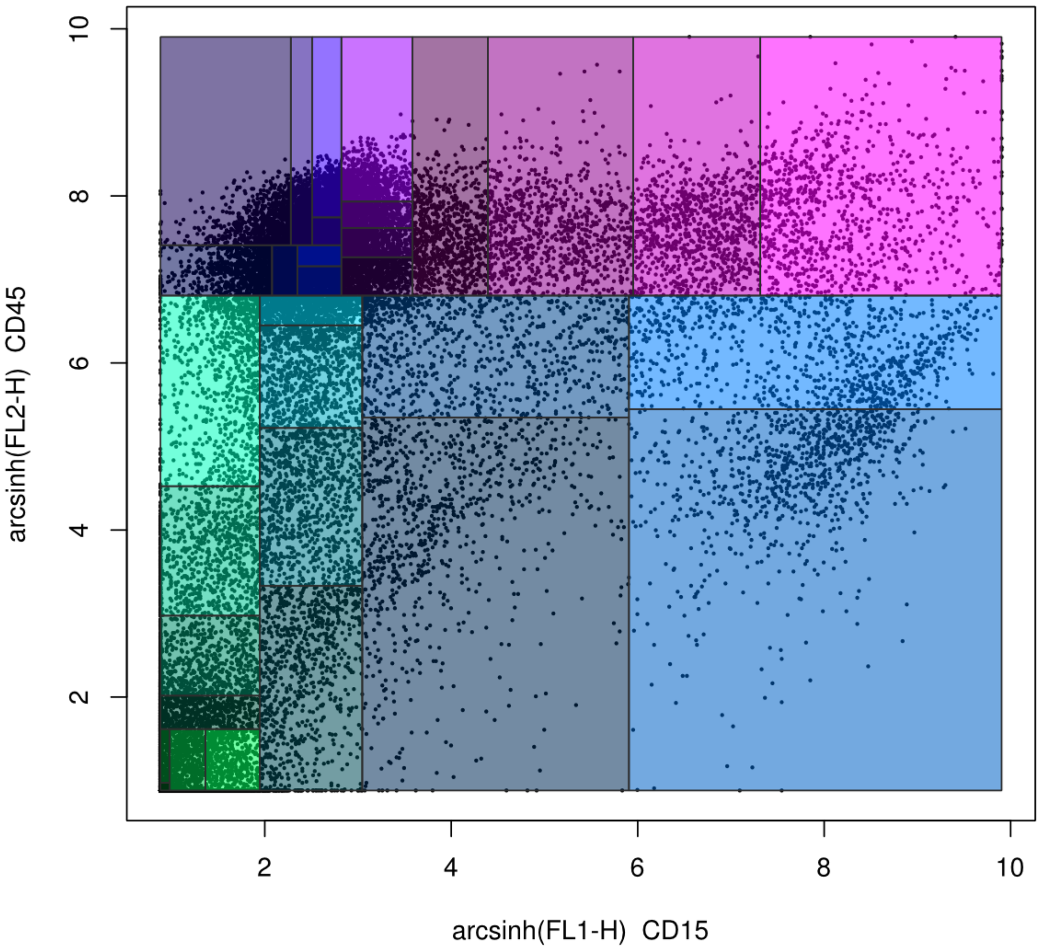

Figure 2.5: An example of frequency difference gating, created using the flowFP Bioconductor package. The dots represent individual events in an FCS file. The rectangles represent the bins.

bins can be used as gates and used for subsequent analysis similarly to automated gating methods.

Combinatorial Gating

High-dimensional clustering algorithms can perform well for identifying larger cell populations, but tend to perform more poorly at identifying rare cell types [5].The full complexity of high-dimensional data can be large – where there are M dimensions and each is divided into positive and negative, there may be as many as 2^M disjoint populations. Methods for deciding on the number of clusters tend to predict much smaller numbers, resulting tin the merging of many of the smaller clusters together. Matching these small cell populations across multiple samples is even more challenging, and has few solutions [47].

An alternative to high-dimensional clustering is to identify cell populations using one marker at a time and then combine them to produce higher dimensional clusters. This functionality was first implemented in the commercial software, FlowJo [48]. The flowType algorithm builds on this framework by allowing the exclusion of the markers [3] This enables the development of statistical tools (e.g., RchyOptimyx) that can investigate the importance of each marker and exclude high-dimensional redundancies. [4].

2.1.5 Diagnosis and Discovery

After identification of the cell population of interest, cross-sample analysis can identify phenotypical or functional variations that are correlated with an external variable (e.g., a clinical outcome). These studies can be partitioned into two main groups of Diagnosis and Discovery.

Diagnosis

In these studies, the goal usually is to diagnose a disease (or a sub-class of a disease) using variations in one or more cell populations. For example, one can use multidimensional

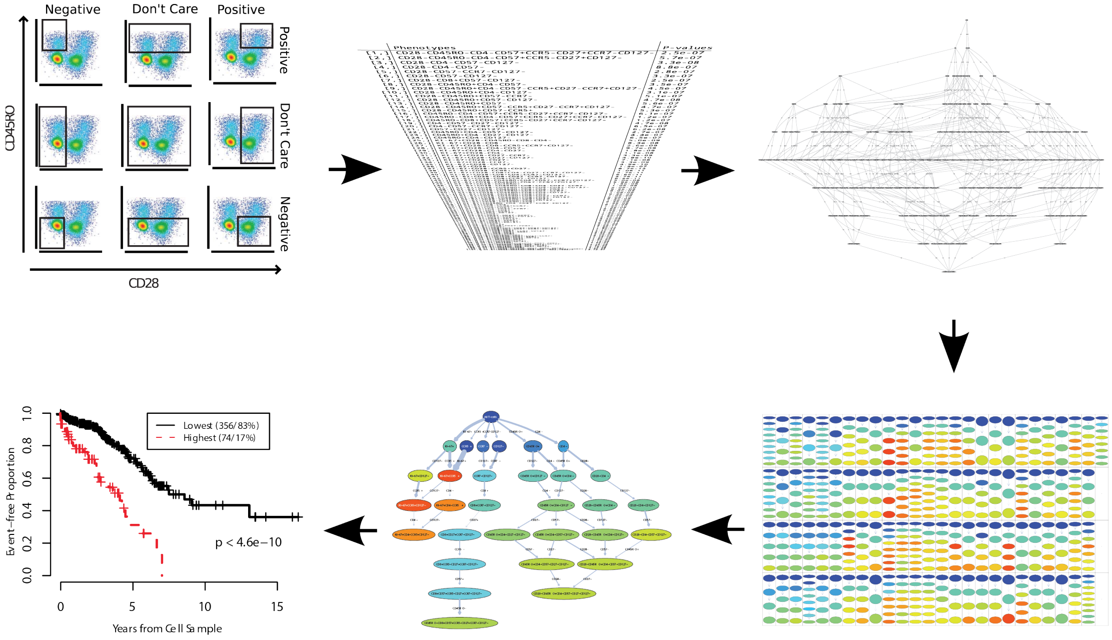

Figure 2.6: Overview of the flowType/RchyOptimyx pipeline for identification of correlates of protection against HIV: First, tens of thousands of cell populations are identified by combining one dimensional partitions (panel one). The cell populations are then analyzed using a statistical test (and bonferroni's method for multiple testing correction) to identify those correlated with the survival information. The third panel shows a complete gating hierarchy describing all possible strategies for gating that cell population. This graph can be mined to identify the "best" gating strategy (i.e., the one in which the most important markers appear earlier). These hierarchies for all selected phenotypes are demonstrated in panel 4. In panel 5, these hierarchies are merged into a single graph that summarized the entire dataset and demonstrates the trade-off between the number of markers involved in each phenotype and the significance of the correlation with the clinical outcome (e.g., as measured by the Kaplan–Meier estimator in panel 6). Figure reproduced in part from [3] and [4], both of which are in the public domain.

clustering to identify a set of clusters, match them across all samples, and then use supervised learning to construct a classifier for prediction of the classes of interest (e.g., this approach can be used to improve the accuracy of the classification of specific lymphoma subtypes [49]). Alternatively, all the cells from the entire cohort can be pooled into a single multidimensional space for clustering before classification.[50] This approach is particularly suitable for datasets with a high amount of biological variation (in which cross-sample matching is challenging) but requires technical variations to be carefully controlled [51].

Discovery

In a discovery setting, the goal is to identify and describe cell populations correlated with an external variable. Similar to the diagnosis use-case, cluster matching in high-dimensional space can be used for exploratory analysis. However, this approach produces cell populations which can be difficult to visualise due to their high dimensionality, and also difficult to characteristically immunophenotype (for example if their shape includes twists and folds in higher-dimensional space).[50, 52] Finally, combinatorial gating approaches have been particularly successful in exploratory analysis of FCM data. Simplified Presentation of Incredibly Complex Evaluations (SPICE) is a software package that can use the gating functionality of FlowJo to statistically evaluate a wide range of different cell populations and visualize those that are correlated with the external outcome. flowType and RchyOptimyx (as discussed above) expand this technique by adding the ability of exploring the impact of independent markers on the overall correlation with the external outcome. This enables the removal of unnecessary markers and provides a simple visualization of all identified cell types. In a recent analysis of a large (n=466) cohort of HIV+ patients, this pipeline identified three correlates of protection against HIV, only one of which had been previously identified through extensive manual analysis of the same dataset [5].

2.2 Acute Myeloid Leukemia

AML is a cancer of white blood cells involving the blood and bone marrow [53]. It is a highly heterogeneous disease, with several dozen recognised subtypes [54]. AML is relatively rare in the general population, with a prevalence of 3.8 cases per 100,000. However, it is more common among adults over 65 (17.9 per 100,000) [55], and has a very poor outlook, with median survival ranging from 7 – 12 months [55]. Standard therapies, including chemotherapy and allogeneic bone marrow transplantation, are harsh and often poorly tolerated due to the advanced age of AML sufferers.

Over the past decade, risk stratification has greatly improved, enabling greater support of higher-risk patients and more informed patient decision making regarding trade-offs between therapy risks and benefits. Much of this improvement has resulted from the recognition, in the late 1990s, that several karyotypic abnormalities are recurrent in AML, which confer either favourable or dismal prognoses [56]. Using cytogenetic risk factor, patients are now typically divided into three risk categories – favourable (5-year OS: 45%–85%), intermediate (5-year OS: 20%–40%) and adverse (5-year OS: 5% – 20%) [57]. Initially, these categories were based solely on cytogenetics, with all normal-karyotype patients being placed in the intermediate risk category. However, since the mid-2000s, molecular genetic markers have been added, helping to place some Normal karyotype (NK) patients in the favourable category [57, 58].

These refinements in risk stratification have slightly improved treatment outcomes, mainly through more targeted supportive care [57]. However, it is clear that stratification could be further improved. Furthermore, therapies could be enhanced, both in terms of their specificity for subtypes of AML and in their general ability to cure the disease [57]. Deep exploration of the biology of AML through high-throughput screens and accompanying bioinformatic data mining is underway towards this end.

Table 2.1: Clinical features and risk stratification of AML. Reproduced from [57].©2013 Elsevier, re-used by permission.

	**Favorable risk** (5 yr OS: 45%-80%)		**Intermediate risk** (5 yr OS: 20%-40%)		**Adverse risk** (5 yr OS: 5%-20%)	
	40%-45% of AML cases		25%-35% of AML cases		25%-30% of AML cases	
	Aberration	Frequency (%)	Aberration	Frequency (%)	Aberration	Frequency (%)
**Prognostic category**	• t(15:17)	7-12	• NK with *FLT3*-ITD	15-20	• 11q23,	3-5
	• t(8:21)	5-8	• NK with *NPM*^{WT} & no *FLT3*-ITD	10-17	• inv(3)/t(3;3)/*EVI-1*	~1
	• inv(16)	5-8	• t(9:11)	2-3	• t(6;9)/*DEK-NUP214*	~1
	• NK with *NPM*^{mut} & no *FLT3*-ITD	18-25	• Other cytogenetic abnormalities not included elsewhere	5-8	• -7/7q-	3-5
	• NK with biallelic *CEBPA*^{mut}	6-12			• -5/5q-	2-3
					• 17p deletions	~2
					• CK	10-12
**Current therapies**	Standard induction cytotoxic therapy ("3+7"), followed by postremission therapy with high dose cytarabine<br>ATRA as a differentiating agent with anthracycline based chemotherapy in t(15;17)		Standard induction cytotoxic therapy ("3+7"), followed by postremission consolidation<br>Allogeneic HSCT should be considered as main modality of consolidation, especially in patients with *FLT3*-ITD		Standard induction cytotoxic therapy ("3+7"), although dismal outcome with chemotherapy alone<br>Allogeneic HSCT should be offered in clinical remission 1<br>Consider use of investigational and novel agents	

2.2.1 Leukemia Subtypes and Heterogeneity

The first systematic classification of leukemias was the French-American-British (FAB) system [59], which classed acute leukemias based on their cellular morphology. The FAB system identified three classes of acute lymphoblastic leukemia and seven classes of AML [59]. Over the next two decades, immunophenotyping of surface and intracellular markers by immunohistochemistry and flow cytometry became a routine addition to morphological studies [60, 61]. Immunophenotyping could distinguish between AML and ALL in cases without morphological differentiation [60], with up to 98% accuracy [61] and could also provide insight into the process of hematopoiesis, which leukemias are believed to be a perturbation of [60, 61]. Purely immunological classifications were then proposed [62], and with the inclusion of cytogenetic information, the third World Health Organization (WHO) Classification of Tumours of Haematopoietic and Lymphoid tissues [63] was created. This was followed in 2008 by the fourth WHO classification [54], which additionally incorporated molecular genetic markers.

The two molecular genetic markers included in the WHO 2008 are mutations in the nucleophosmin (*NPM1*) and CCAAT/enhancer binding protein-alpha (*CEBPA*) genes,

Table 2.2: Recurrent non-karyotypic genetic aberrations in AML, including unmutated but overexpressed genes and miRNAs. Reproduced from [57].©2013 Elsevier, re-used by permission.

	Gene	Frequency (in NK-AML) and comments
Signalling	*FLT3*-ITD	20%-25% (28%-35%); High blast count; Poor prognosis especially in cases with high mutant to WT allelic ratio
	FLT3-TKD	5%-7% (10%-14%); Prognostic impact remains controversial
	NRAS	10% (9%-14%); Enriched in CBF AML; Prognosis unknown
	C-KIT	<5% (<5%); 25-30% in CBF leukaemia
	PTEN	<2% (2%); Prognosis unknown
Transcription factors (TF)	*NPM1*	25%-30% (40%-65%); M4 blast morphology lacks CD34 expression; Hox gene upregulation; Favorable prognosis in the presence of *FLT3*^{WT}; Female preponderance
	CEBPA	5%-10% (10-19%); Favorable prognosis if biallelic mutation
	RUNX1	5%-13% (6-25%); Enriched in trisomy 13 and FAB M0; Poor prognosis
	WT1	10% (10%-13%); Associated with M0 FAB type; Poor prognosis
	TP53	2%-4% (<2%); Predominantly in CK-AML; Very poor prognosis
Epigenetic modifiers	*DNMT3A*	20%-25% (32%-35%); Heterozygous R882 mutations account for 40%-60% of mutations; Poor prognosis in NK-AML
	IDH1/IDH2	12%-22% (25%-30%); Mutant *IDH1 & 2* are mutually exclusive; *IDH1* mutations enriched in patients with *NPM1*^{mut}; IDH1 is localized in cytoplasm and peroxisomes
	TET2	7%-15% (15%-23%); Mutually exclusive to *IDH1/2* mutations; More prevalent in secondary AML especially MPN
	ASXL1	3% (3%-5%); Poor prognosis
	EZH2	<2% (<1%); Enriched in MDS/MPN; Prognosis unknown
	MLL-PTD	<2% (2%-5%); Enriched in trisomy 11
Overexpressed	*EVI-1*	Deregulated in inv(3)(q21q26); Poor prognosis
	MN1	Poor response to chemotherapy; Correlated with *NPM*^{WT} and high BAALC expression
	BAALC	High expression in NK and +8; Poor prognosis
	ERG	Poor prognosis in CK and NK AML
	miR-181	Increased in FAB M1/M2, *CEBPA*^{mut}; Favorable prognosis

both of which confer favourable prognosis [54, 64–68]. However, additional markers are known, including internal tandem duplications in the Fms-like tyrosine kinase 3 (*FLT3*) gene, which are associated with poor prognosis [69]. Later studies have tended to show that *NPM1* alone is insufficient to predict a significant difference in prognosis, and that it is only the combination of *FLT3*-ITD with wild-type *NPM1* which has prognostic value [58]. Furthermore, the individual genetic markers found in these studies have typically been relatively frequently-occurring (see Table 2.2), yet have not fully accounted for the diversity of AML.

This has led more recently to high-throughput sequencing studies. The first AML genome was published in 2008 [70], and the first multi-patient sequencing effort was published a year later [71]. Since then, the cancer genome atlas (TCGA) sequencing effort has published a landscape paper, containing the genomes, transcriptomes, miRNA and DNA-methylation for a cohort of 200 AML patients [72]. Some of the results of these and other recent efforts are summarised in Table 2.2.

2.2.2 Flow Cytometry Immunophenotyping

AML is routinely diagnosed by the use of flow cytometry to determine the presence or absence of specific antigens present on single cells (their immunophenotype) [73]. Today, flow cytometry immunophenotyping is a critical step in the process of clinical decision making about leukemias [54, 73, 74]. It can be used to identify the lineage and maturity of cells, detect abnormal cells, document the phenotype of abnormal cell populations, diagnose a specific disease entity (or suggest further testing to do so), and provide information that may be of prognostic value [73] Although its use is somewhat restricted by cost [73], it is employed widely in most cases where other factors suggest a diagnosis of leukemia.[74] The ability of the technique to sensitively characterise leukemias may, in conjunction with omics methods, lead to personalized treatment regimens.[75] Table 2.3. lists some of the more important surface and intracellular proteins detected by flow cytometry, along with their specific utility. Figure 2.7 illustrates how commonly used

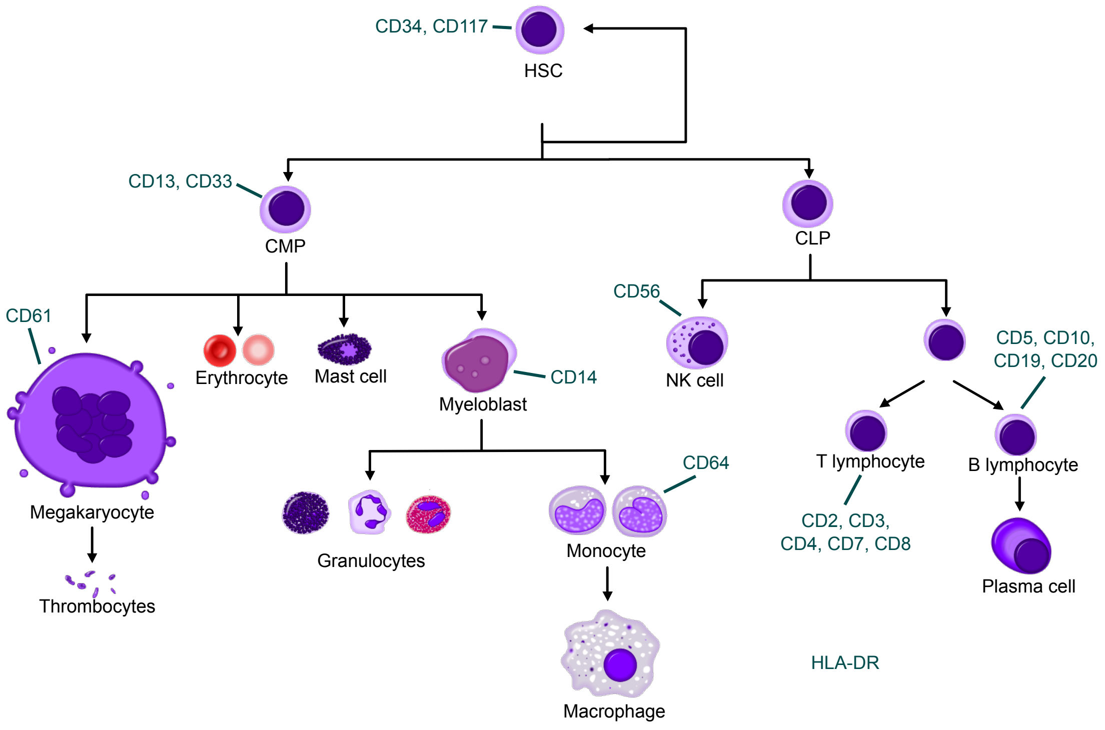

Figure 2.7: Hematopoeitic markers detected by flow cytometry for leukemia diagnosis. Figure adapted from work ©Mikael Häggström, re-used under the terms of the CC-BY-SA 3.0 license.

immunophenotypic markers correspond to normal hematopoeisis.

2.2.3 Multitube Flow Cytometry Data

The number of markers typically assayed in leukemia diagnosis exceeds the number of markers which commonly in-use instruments can measure. For example, one standard panel recommends assaying 32 markers on 8-colour instruments [76]. To achive this, standard practice is to aliquot samples into multiple tubes, with common markers in each [74, 76]. This is illustrated in Fig 2.8. In leukemia diagnosis, CD45 is typically included in each tube and used to identify leukemic blast cells manually in a scatterplot of CD45 vs side scattered light (SSC) [73, 77–79]. An example of the manual gating out of blast cells in the CD45 and SSC dimensions is illustrated in Fig 2.9.

In most cases leukemic blasts fall within the same region of the scatter plot ($SSC^{low}CD45^{dim}$), as shown in Fig 2.10a. However, there are many exceptions, as shown in Fig 2.10b.

Table 2.3: Markers commonly assayed in leukemia diagnosis significance, after [73]. All are measured on the cell surface unless otherwise indicated by the prefix "intra" for parameters measured intracellularly.

Parameter	Normal staining	Clinical utility
CD2	T and NK cells	Indicator of T or NK lineage, but sometimes expressed in AML
CD3	Mature T cells	Indicates T lineage, but may be aberrantly lost
CD4	T cell subset, monocytes	Classification of mature T neoplasms
CD7	T and NK cells	Indicates T lineage, but may be aberrantly lost
CD10	Immature T/B cells, subset of mature T/B, neutrophils	Frequently present in ALL
CD13	Myeloid and monocytic cells	May be aberrantly expressed in B-ALL, AML, MDS
CD14	Monocytes	Indicates monocytic differentiation, but not a sensitive marker of immature monocytes
CD19	All B cells	Indicates B lineage. Usually absent in plasma cell neoplasms.
CD20	Mature B cells, some T cells	Supports B cell lineage
CD33	Myeloid and monocytic cells	May be aberrantly expressed in B-ALL, AML, MDS
CD34	B, T precursors and myeloblasts (not mature cells)	Indicates leukemic blasts
CD56	NK and NK-like T cells	Indicator of NK differentiation, but may be present in AML
CD61	Megakaryocytes and platelets	Megakaryocyte differentiation
CD64	Monocytes and intermediate neutrophilic precursors	Identification of monocytic differentiation, but may be aberrantly expressed in AML
CD117	Immature neutrophils and mast cells	Identification of myeloblasts, but may be expressed by more mature cells
HLA-DR		
intraCD3	All mature and immature T cells	Indicator of T or NK lineage
intraCD22	Cytoplasmic in early B cells, surface in mature	Indicates B lineage; intensity often differs between subtypes of B neoplasms
intraCD79a		
intraTdT	B and T precursors	Indicates ALL, but also found in some AML
intraMPO	Neutrophilic and monocytic cells	Indicator of myeloid differentiation

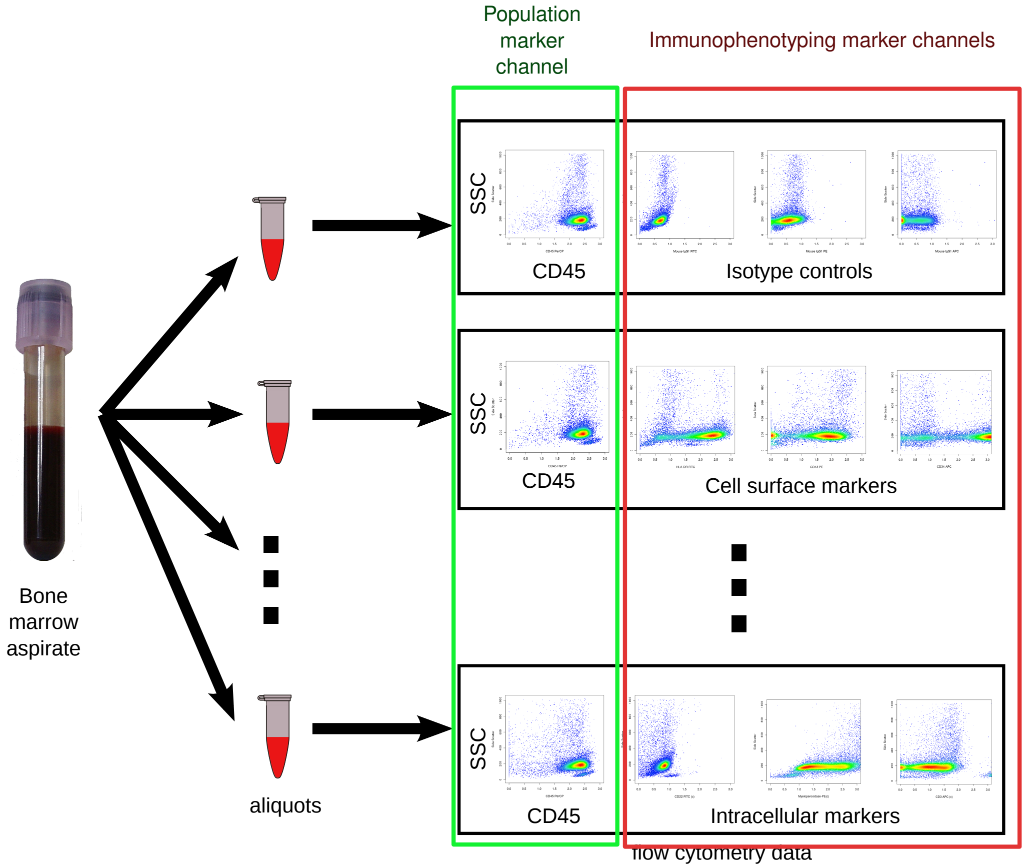

Figure 2.8: Multitube flow cytometry data on a four-colour flow cytometer. This figure shows how a sample is aliquoted into multiple tubes, each containing CD45 and a combination of three markers unique to each tube. In this example, the first tube contains negative controls, while subsequent tubes contain markers of interest.

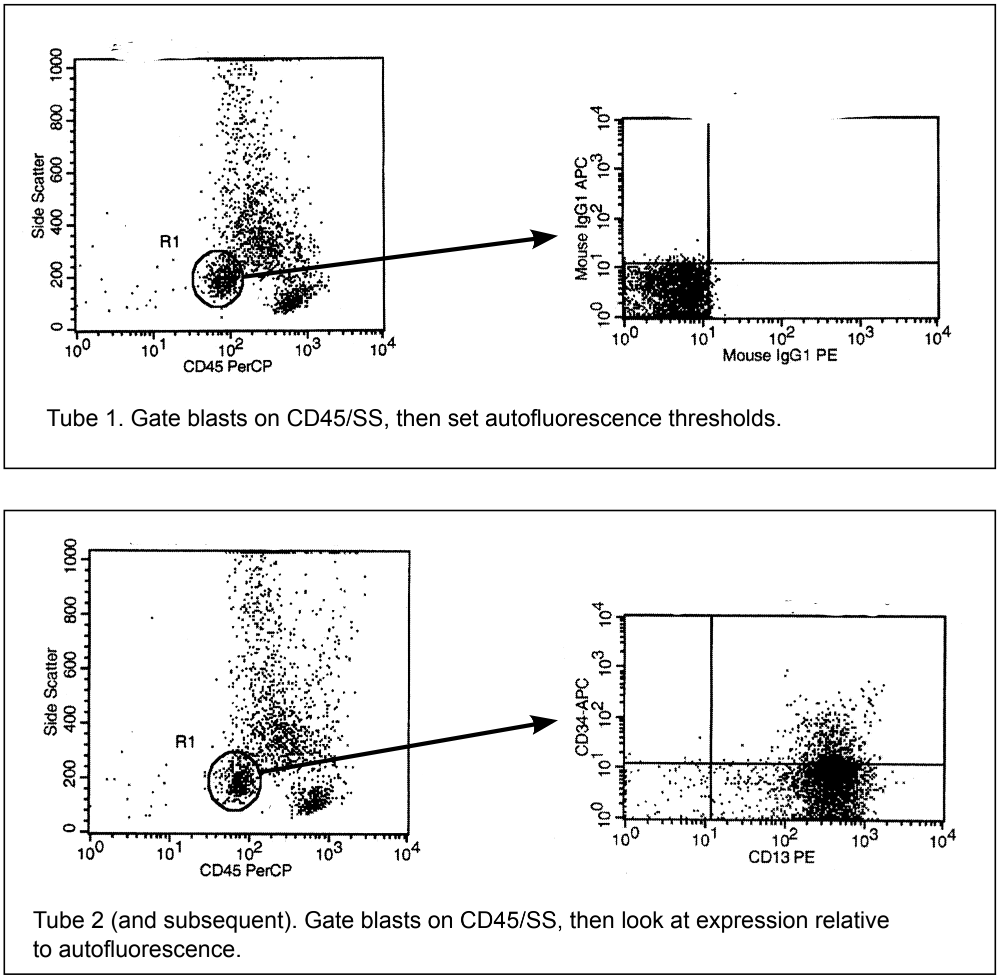

Figure 2.9: Manual analsysis of multitube flow data for leukemia immunophenotyping. Blast cells are identified by the pathologist based on their CD45 (typically dim) and side scatter (typically low). The first tube is usually stained with negative control markers in the channels besides CD45, and this staining is used to set a threshold distinguishing positive from negative marker expression. In the subsequent tubes, this threshold is used to compute the proportion of cells which express each marker. Figure from Dr. Bakul Dalal, VGH Hematopathology. Used by permission.

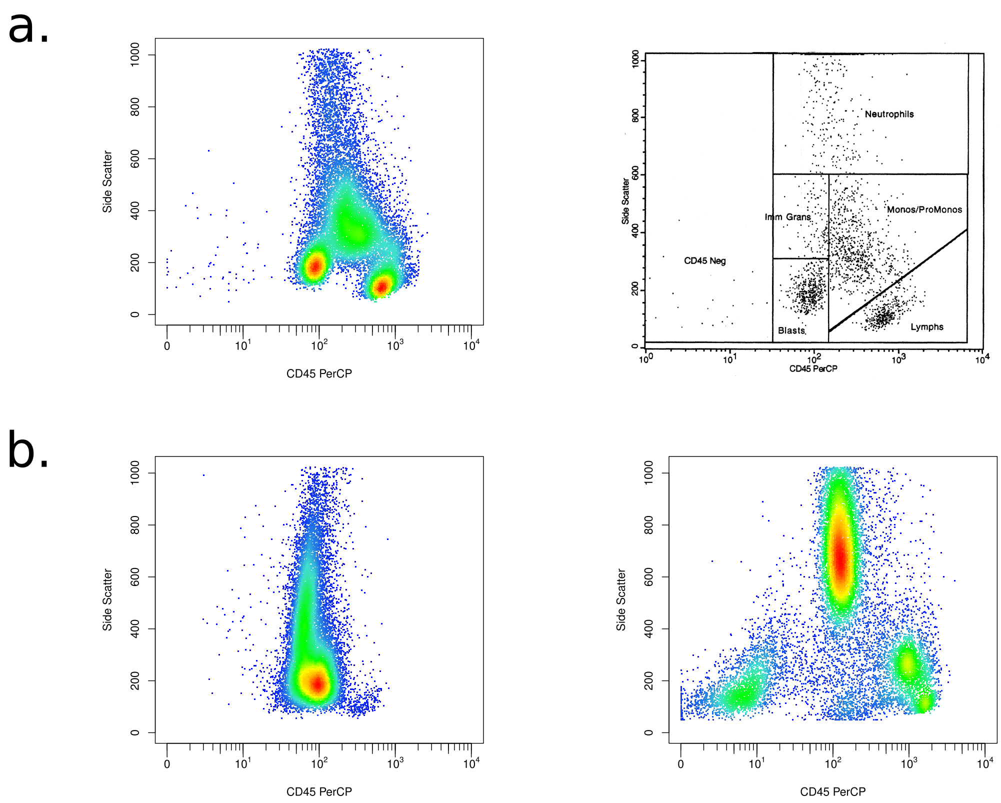

Figure 2.10: Gating leukemic blasts in CD45/SSC: ideal vs reality. For many patients, identifying blasts is relatively simple, and a generic gating net can be used (a). However, exceptions such as those shown in (b) are common. Gating blasts in these cases requires more elaborate analysis and deeper knowledge, and would be challenging to automate. Gating net from Dr. Bakul Dalal, VGH Hematopathology. Used by permission.

While automating blast identification and immunophenotyping would be desirable, the movement of both the blasts and other cell populations in some cases would confound attempts to do so. A more realistic approach to automated analysis of leukemia data would be to combine tubes from each patient together without knowledge of the cell types within, and then compare that data across patients.

2.2.4 Bioinformatic Approaches for Combining Multitube Data

To exploit multitube flow cytometry data computationally, a method has been developed for combining multiple aliquots based upon the location of cells in the common parameters. This uses K-nearest neighbours to match cells closest to each other in the common

parameters, and combine their expression in other dimensions [80]. However, as others have shown [81], and I show later in this thesis, populations defined in terms of population markers are frequently made up of a mixture of cell types, and NN consequently tends to produce spurious combinations of markers. This makes the merged output from NN poorly suited for applying the deep profiling techniques, such as flowType and SPADE, as it tends to skew the counts of cell types. One proposed solution to this is to constrain the nearest-neighbours mapping with clustering incorporating domain knowledge [81]. However, this latter method requires that all cell types expected to be present be pre-specified in order to parameterise the clustering step. So, although well suited to diagnostic pipelines where the goal is to quantify known cell types, it is poorly suited for discovery of new cell types. There remains a need for a multitube combination method for FCM data that produces conservative, non-imputed data suitable for deep profiling.

2.2.5 Surveying the Immunophenotypic Landscape of Leukemia

Despite several broad and comprehensive studies of AML in terms of its genome, transcriptome, miRNA, and methylation status, only one study has performed a comprehensive bioinformatic analysis of AML immunophenotypes, and no studies have incorporated this with other data. That single study examining cell subtypes in AML was carried out on a small cohort (ten normal and four leukemic), and only for one single four-colour flow cytometry panel (containing CD13, CD33, CD45 and CD34) [82]. The existence of a method for accurately analysing multitube flow cytometry data (as is often available both retrospectively and prospectively in AML diagnosis) would enable such surveying of the immunophenotypic landscape of AML. In Chapter 4, I present flowBin, a method I developed for combining multitube flow cytometry data, and in Chapter 5 I detail several analyses I performed using flowBin on retrospective multitube flow cytometry data from AML patients.

2.3 Strand-seq

Strand-seq is a method for directional, low-coverage sequencing of DNA template strands in single cells [83]. In strand-seq, cells are cultured for one cycle of cell division in the presence of bromodeoxyuridine (BrdU). BrdU is a synthetic nucleotide which subsitutes for thymidine in the newly synthesised DNA strands of the daughter cells. Single cells are then sorted into separate plate wells, where their DNA is extracted and fragmented by micrococcal nuclease digestion. Forked adapters are ligated to each double stranded fragment, enabling PCR replication from either direction. The adapters used for each cell contain a unique 6-nucleotide index sequence, allowing multiple cells to be sequenced together and later deconvoluted. Prior to PCR, the BrdU-incorporating strand is nicked at BrdU sites by exposure to ultraviolet light and Hoechst 33258. During PCR, only the unfragmented template strand is amplified for subsequent Illumina sequencing. The directionality of the short reads thus corresponds to the template strand inherited by the daughter cell.

The resulting sequence and directionality data can then be aligned back to the reference genome for the organism. The read counts in each direction can be visualised as a histogram along each chromosome (Fig 2.11d). For autosomes, since there are two physical chromosomes per reference sequence, the template strand state can only be inferred for both chromosomes at once, as being one of: WW, WC or CC. Visually inspecting the read count histograms can indicate this state (see Fig 2.11d).

2.3.1 Detection of Sister Chromatid Exchange Events

Strand-seq was developed to test the Silent Sister [85] and Immortal Strand Hypotheses [86], which propose that template strand segregation during mitosis may be asymmetrical in some cell types, for functional reasons. SSH proposed that this might be a determinant of stem cell fate, while ISH proposed that this might be for the purposes of maintaining a non-copied (and higher integrity) template strand in stem cells. As such, the goal of strand-seq was to test whether strand inheritance during cell division was random or

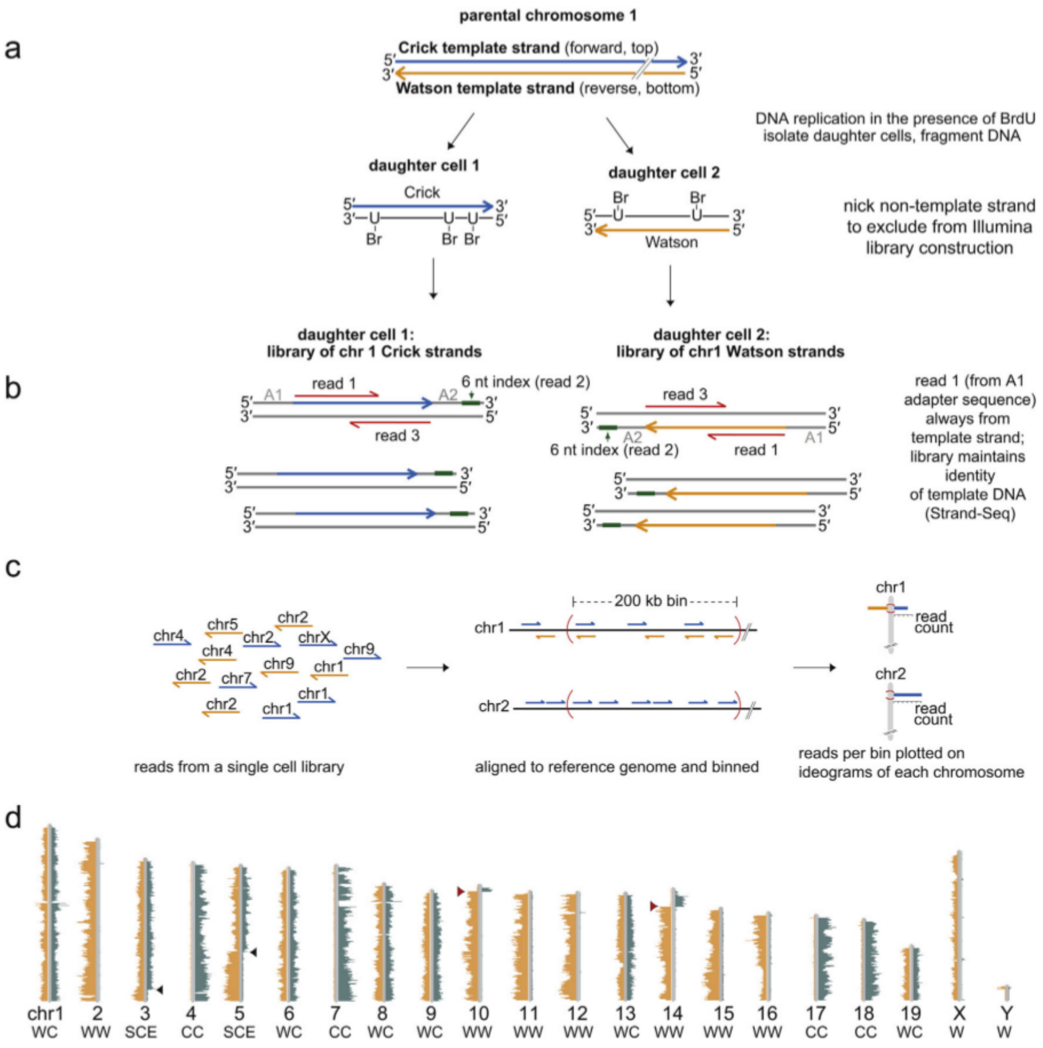

Figure 2.11: Overview of strand-seq protocol. Figure reproduced from [84](©2013 Elsevier), parts of which were reproduced from [83] (©2012 Nature Publishing Group). Re-used by permission of both copyright holders.

segregated [84].

While strand-seq has yet to confirm either hypothesis, early results indicated that it had other applications, most notably sensitive detection of sister chromatid exchanges (SCEs) [83]. SCEs are exchanges of genetic material between identical sister chromatids. They occur as a result of double stranded breaks being repaired during homologous recombination [87]. SCEs occur spontaneously at an average rate of approximately 10 per mitosis in normal human cells [87]. Elevated rates of SCE is an indicator of genomic instability, and is an important diagnostic test for Bloom's Syndrome [88].

SCEs show up in strand-seq data as marked discontinuities in the strand inheritance state for a particular chromosome pair, allowing them to be detected at a resolution approaching the read depth of the strand-seq (Fig 2.11d) [83]. Detecting SCEs in this way can reach a resolution of the order of kilobases, several orders of magnitude finer than previous techniques [83].

2.3.2 Applications of Strand-seq to Leukemia

Genomic instability is heavily interrelated with cancer [89], and has been shown to be a useful prognostic marker in some cancers [90–92]. However, detection of genomic instability depends on either gene expression signatures [92] or breakpoint detection [90, 91]. Gene expression signatures are an indirect measurement, while breakpoints are late indicators, occurring only after DNA repair mechanisms have been overwhelmed. By contrast, rates of sister chromatid exchange are a direct measure of genomic instability, before misrepaired breaks occur. As such, strand-seq, as a low-cost, direct measure of genomic instability, could be useful for the diagnosis and staging of cancers generally, and leukemia more specifically.

Furthermore, although it has been established for more than two decades that tumours are primarily monoclonal in origin [93], interest has arisen in recent years in unravelling the subsequent clonal diversification and evolution [94, 95]. Understanding cancer clonal evolution is likely to shed light on clinically catastrophic clonally-driven

processes like metastasis and chemotherapy resistance, and lead to better therapies to overcome these [96]. One facet of this evolutionary process is the acquisition and loss of genomic rearrangements. These are currently detected by fluorescence in-situ hybridisation (FISH), using cDNA probes specific for known rearrangements [97]. While FISH is able to detect rearrangements in single cells, results require visual inspection of sample micrographs by expert pathologists, and are thus labour-intensive and potentially subjective. Strand-seq has the potential to enable the rapid and non-subjective identification of genomic rearrangements, including those not yet characterised, on a single-cell basis [83]. This could be a powerful, low-cost tool for elucidating the clonal structure of cancers generally, and leukemias specifically.

2.3.3 Automated Analysis of Strand-seq Data

In the original strand-seq study, all analysis was performed manually by visual inspection of read count histograms [83]. This was a limiting factor in the usefulness of the technology Chapter 6 described my efforts, in collaboration with Mark Hills, to develop software tools to automate these processes, as well as later work we undertook to develop tools to use strand-seq for assembly of early build genomes.

Chapter 3

Enhanced FlowType for High-Dimensional Single Cell Data

3.1 Introduction

Flow cytometry has undergone a "chromatic explosion" over the past decade and can now measure 17 markers at once for each of hundreds of thousands of individual cells [15]. Since then, mass cytometry has enabled measurement of 30–45 markers per cell [98], while single-cell multiplexed RT-qPCR can measure 50–96 mRNAs per cell [17]. The growth in high-throughput single-cell data continues to outpace development of corresponding bioinformatics techniques [15]. To answer this challenge, flowType [3] and RchyOptimyx [4] were developed. FlowType uses partitioning of cells, either manually or by clustering, into positive or negative for each marker to enumerate all cell types in a sample. RchyOptimyx measures the importance of these cell types by correlating their abundance to external outcomes, such as disease state or patient survival, and distills the identified phenotypes to their simplest possible form. These packages have been used to identify several novel cell populations correlated with HIV outcome [3]. More recently, this pipeline has been used to evaluate standardised immunological panels [99], to optimise lymphoma diagnosis [100], and to analyse a range of other clinical data (unpublished).

However, the higher dimensionality of data produced by mass cytometry generates

up to $3^{45} \approx 10^{21}$ possible cell types, with an even greater number (up to $3^{96} \approx 10^{45}$) for single-cell qPCR; these magnitudes are beyond the capabilities of flowType and RchyOptimyx. Furthermore, flowType and RchyOptimyx have thus far only treated cells as being either positive or negative for a marker. In practice, many biomarkers can have a range of expression levels such as "dim" and "bright". In this chapter, I detail architectural improvements I and my co-authors undertook to flowType and RchyOptimyx to overcome these limitations.

3.2 Methodology

Our primary challenge was to enable flowType to generate a number of cell types tractable on most common workstations as well as nodes in compute clusters (e.g., those with 4–12GB of RAM). To this end, we developed a new version of flowType, which I hereafter denote as flowType dynamic programming (flowType-DP), to distinguish it from the old version, which I denote as flowType brute force (flowType-BF).

3.2.1 Dynamic Programming Implementation

To improve computation time, flowType-DP uses a dynamic programming approach, which exploits the fact that cell types can be arranged into a hierarchy, and membership of any given cell type over n markers is equal to the intersection of one of its parent types (over $n-1$ markers) with a single-marker cell type. FlowType-DP first enumerates all cell types involving only 1 marker by simple partitioning and then iterates over $2, ..., k$ markers, computing all cell types for each level n by set intersections between corresponding cell types in levels $n-1$ and 1.

For example, membership of the cell type $CD45^{++}CD117^{+}CD34^{-}$ is computed as

follows:

$$\{\text{CD45}^{++}\text{CD117}^{+}\text{CD34}^{-}\}$$
$$=\{\text{CD45}^{++}\text{CD117}^{+}\}\cap\{\text{CD34}^{-}\}$$
$$=\{\text{CD45}^{++}\}\cap\{\text{CD117}^{+}\}\cap\{\text{CD34}^{-}\}$$

For added speed, the core flowType-DP functionality was implemented in C++. To optimise the calculation of set intersection, the set of cells belonging to each cell type was represented using a bit string, with one bit per cell. The Boost library dynamic_bitset class was used to store these bit strings. This allowed set intersection to be computed using a bitwise AND operator, which translates to a single machine instruction.

3.2.2 Breadth-First Strategy

FlowType-BF completely enumerates all cell types over all $[1, ..., m]$ markers. As noted in the introduction, this can easily result in an intractable number of cell types. Furthermore, it is common with flowType that the most significant cell types are made up of far fewer markers than the full number available. For example, in the original flowType paper, despite 11 markers having been used, the cell types most significantly correlated with survival were defined over 2, 3 and 7 markers, respectively. FlowType-DP thus enables a user to use a breadth-first strategy of enumerating all cell types defined over a subset of $k \leq m$ markers only.

FlowType-DP provides a memory use estimation function, to assist users in finding a k that fits within the limits of their hardware. The number of cell types N for a given cutoff k can be computed using a dynamic programming algorithm. Where N_i denotes

the number of cell types involving i markers, in a dataset with m total markers:

$$N_1 = m \tag{3.1}$$

$$N_2 = N_1 \times N_1 \tag{3.2}$$

$$N_i = N_{i-1} \times N_{i-2} \tag{3.3}$$

For a given k, flowType recursively computes $N_{1 \cdots k}$, and then computes N and the total memory required M_P (in bytes):

$$N = \sum_{k}^{i=1} N_i \tag{3.4}$$

$$M_P = m \times N \tag{3.5}$$

To count the memory needed to store the bit string representation (M_B), $max(N_i)$ is found, and multiplied by the size of one bit string, as follows (where n is the number of cells):

$$M_B = \frac{max(N_i) \times n}{8} \tag{3.6}$$

The final memory estimate in bytes is taken as the sum of these two numbers $(M_P + M_B)$.

3.2.3 Partitioning into Multiple Expression Levels

To allow partitioning into levels other than positive and negative, a string representation is used for cell types. The string has one integer character for every marker, denoting the partition, or zero if the marker is not used. Values $1, ..., n$ denote partitions 1 to n. For example, if the set of markers were {CD3, CD45, CD13, CD117, CD34} the cell type $CD45^{++}CD117^{+}CD34^{-}$ would be represented by `03021`. RchyOptimyx uses a dynamic programing algorithm for efficiently constructing k-shortest paths [101]. RchyOptimyx'

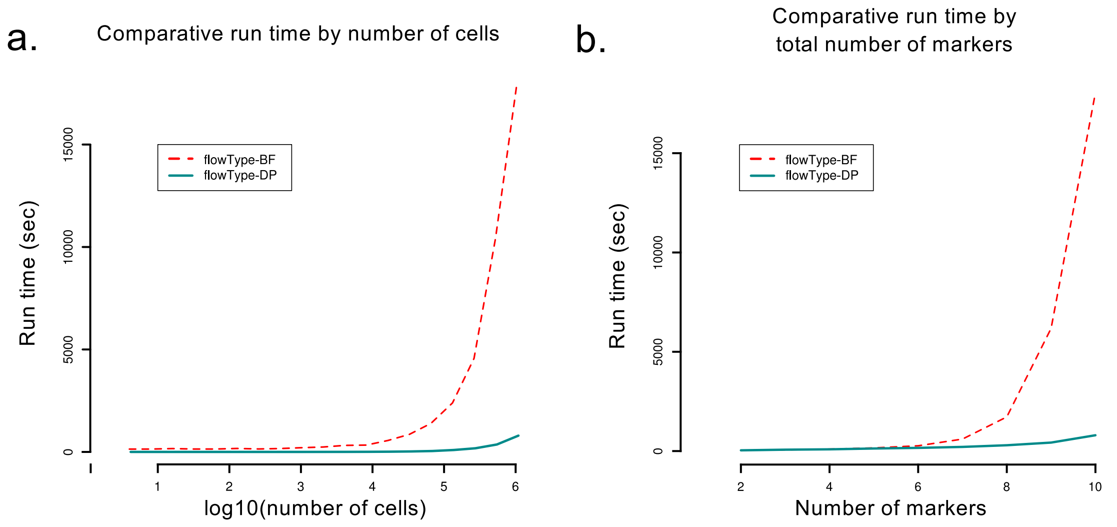

Figure 3.1: Run time comparison of flowType-DP to flowType-BF in terms of number of cells (a) and number of markers (b). Figure is my own work,[2] ©2014 Oxford University Press, used by permission.

graph construction component was modified to be able to handle more than one partition per marker.

3.3 Evaluation

3.3.1 Performance

FlowType-DP was evaluated against flowType-BF on a 10-marker dataset available from Flow Repository (ID FR-FCM-ZZZK) [3]. FlowType-DP showed a substantial speedup over flowType-BF, which increases exponentially with the number of cells and markers. For example, at 10^6 cells and 10 markers, flowType-DP is 14 times faster (see Fig. 3.1). Comparison on larger datasets was not possible, due to the limitations of flowType-BF.

3.3.2 Memory Limits

I also computed the limits for k on a hypothetical machine with 12GB of RAM for samples representative of mass cytometry (Fig. 3.2a) and polychromatic flow cytometry (Fig. 3.2b), both of which would be intractable for flowType-BF. FlowType and RchyOptimyx are now able, within the memory of a common workstation (12GB), to analyze 34-marker

data.

3.3.3 Example Case: Multiple Expression Levels

Finally, to demonstrate the importance of several partitions per marker, I applied flowType and RchyOptimyx to an acute myeloid leukemia sample from Flow Repository (ID FR-FCM-ZZYA) (Fig. 3.3). CD34 is a stem-cell marker typically expressed on AML blast cells. These blasts are also known to have dimly positive CD45 expression and low SSC [102]. By partitioning CD45 and SSC into four and three partitions, and naively running flowType and RchyOptimyx to search for CD34-enriched cell types, I was able to find that the $SSC^{low}CD45^{dim}$ cell type had a high proportion of $CD34^{+}$ cells, as expected. This would not have been possible with only two partitions for each of CD45 and SSC.

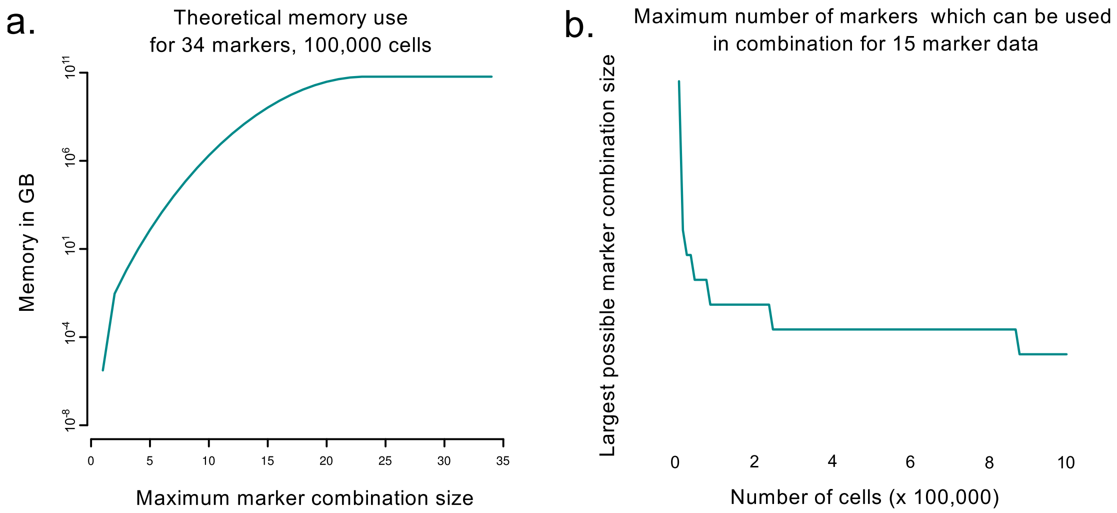

Figure 3.2: Possible thresholds for marker combinations using flowType-DP for typical mass cytometry data (a) and polychromatic flow cytometry data (b). Figure is my own work,[2] ©2014 Oxford University Press, used by permission.

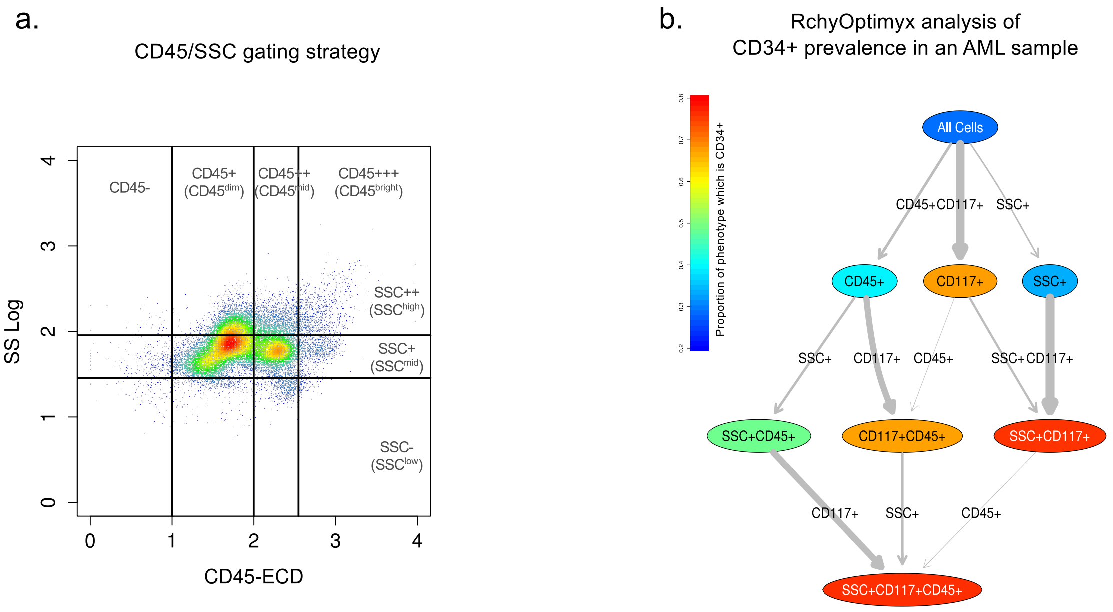

Figure 3.3: Three/four-partition flowType-generated, RchyOptimyx-visualized cell type hierarchy on a bone marrow sample from a patient with AML. Cell population identification strategy used for SSC and CD45, with the CD34-enriched subset highlighted (a). RchyOptimyx analysis showing CD34 enrichment (b). Figure is my own work,[2] ©2014 Oxford University Press, used by permission.

3.4 Discussion

In this chapter I have presented a new, enhanced version of flowType, capable of processing very high dimensional data available from current methods. This new flowType is also substantially faster than the previous version due to being implemented in C++ and optimised using a dynamic programming algorithm. Most importantly, flowType-DP allowed me to use flowType in conjunction with flowBin, the method I describe in Chapter 4. In Chapter 5, I detail two studies in which I applied flowBin and flowType-DP, where flowType-BF would not have been feasible.

One minor disadvantage compared to the flowType-BF is a slight increase in memory usage by the dynamic programming algorithm for storage of the phenotypes. However, this is only an issue on lower-dimensional data, since flowType-BF is incapable of handling higher-dimensional data at all. Another potential but necessary disadvantage of flowType-DP is that it no longer finds all possible cell types on larger data sets (since this would be intractable).

Chapter 4

FlowBin: Deep Profiling of Multitube Flow Cytometry Data

4.1 Introduction

Often in flow cytometry immunophenotyping, the number of proteins needing to be assayed exceeds the number that the cytometer available can measure in a single run. Furthermore, it is often desirable to use negative controls (either unstained or isotype) to counteract technical variation across samples [34]. As a solution to this problem, standard practice is to aliquot a sample into multiple tubes, each of which is run with a disjoint subset of the total panel of proteins. Typically, some of the channels in each tube are taken up by stains which are kept the same across tubes, in order to aid in identifying cell types of interest. This process is common for modern clinical diagnostic flow cytometry data, especially when immunophenotyping leukemias where the standard method is to include the pan-leukocyte marker (CD45) in each tube, and use this in combination with right-angle scattered light (SSC) to identify leukemic blasts in each tube separately [79]. For example, the current standard for leukemia diagnosis established by the EuroFlow consortium recommends an eight-colour multitube panel of overlapping reagents [76].

Without some means of combining tubes together, existing techniques for deep profiling can only be applied serially to each tube in a multitube flow cytometry assay, which results in a substantial loss in depth. This can be illustrated with an example: consider an assay with six tubes containing six markers each, with two of those markers

overlapping (being present) in every tube. The complete number of distinct markers will be $2 + 4 \times 6 = 26$. When examining a binary division of each marker into positive and negative expression, the total number of possible cell types present is $3^{26} \approx 2.5 \times 10^{12}$ [3]. However, working one tube at a time, only 3^6 cell types can be elucidated in each tube, for a total of $3^6 \times 6 = 4374$. For this example, serial analysis can only explore approximately one hundred millionth of the complexity of the phenotype space.

Per-cell nearest-neighbour (NN) merging of tubes attempts to address this. This method is founded on making the assumption that a cell in one tube is identical to its nearest neighbour in another tube in terms of the common population markers [80]. The expression vectors of all the nearest neighbours across tubes are merged, creating a single, high-colour matrix of cellular expression. NN merging has proven effective as part of classification pipelines [38, 76, 103].

However, as others have shown [81], and I show later in this chapter, populations defined in terms of population markers are frequently made up of a mixture of cell types, and NN consequently tends to produce spurious combinations of markers. This makes the merged output from NN poorly suited for applying the deep profiling techniques, such as flowType and SPADE, as it tends to skew the counts of cell types. One proposed solution to this is to constrain the nearest-neighbours mapping with clustering incorporating domain knowledge [81]. However, this latter method requires that all cell types expected to be present be pre-specified in order to parameterize the clustering step. So, although well suited to diagnostic pipelines where the goal is to quantify known cell types, it is poorly suited for discovery of new cell types. There remains a need for a multitube combination method for flow cytometry data that produces conservative, non-imputed data suitable for deep profiling. In this chapter, I describe flowBin, a free, open source R/Bioconductor package that I developed to fulfil this need.

4.2 Design and Implementation

FlowBin is designed to accept multiple FCM assays from the same multitube assay and combine these into a complete matrix of measurements for all the markers. To this end, flowBin consists of four stages: 1) normalization, 2) binning, 3) bin matching across tubes, and 4) expression measurement. These are illustrated in Figure 4.1, and in detail hereafter.

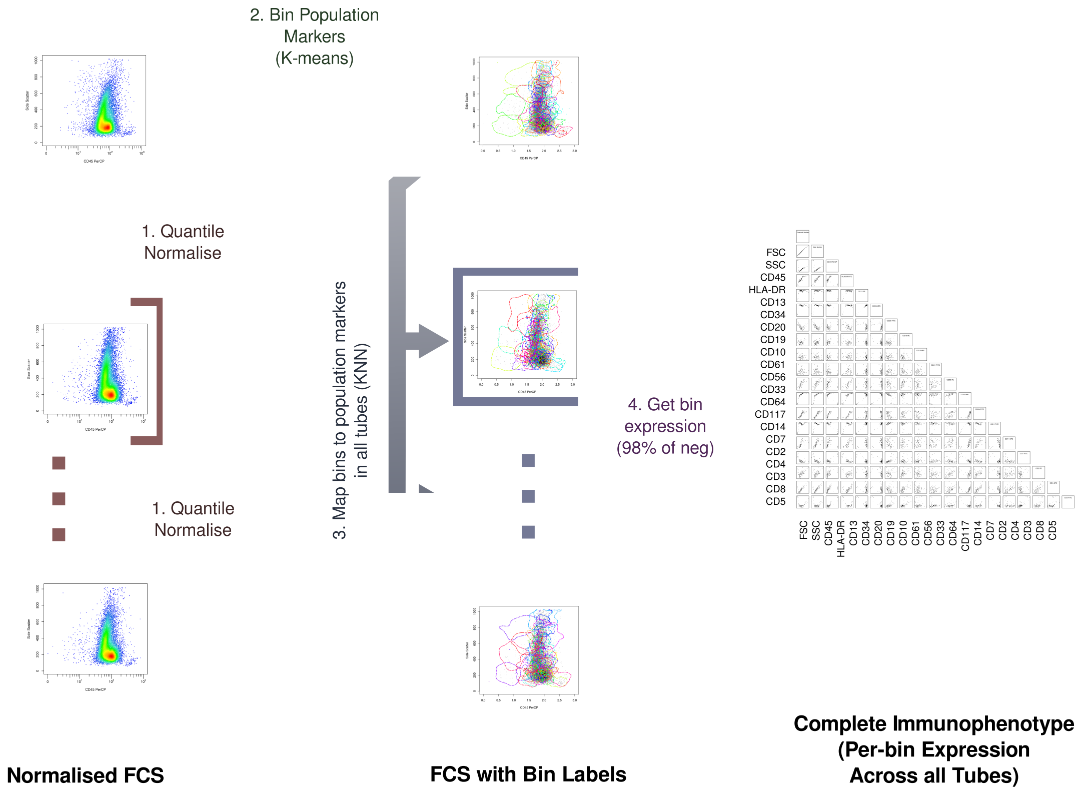

Figure 4.1: Overview of the flowBin pipeline, applied to one multitube sample. 1) Flow cytometry data from individual aliquot tubes is quantile normalized in terms of the common population markers present in every tube. 2) The tubes are then binned in terms of these population markers, using either K-means or flowFP. 3) The bins from the first tube are mapped to the other tubes (by nearest-neighbour mapping for K-means bins, or directly for flowFP bins). 4) The expression of each bin in terms of each phenotyping marker (those markers differing across tubes) is measured. This may be done by taking median fluorescent intensity, normalized median fluorescent intensity, or proportion of cells exceeding the 98th percentile of a negative control. The final result is a high-dimensional matrix containing expression levels for each bin in terms of each unique marker.

4.2.1 Population Marker Normalization

A consideration in combining multitube flow cytometry is that variations between staining patterns across the aliquots need to be minimized [80]. In opposition to this are a host of sources of technical variation, ranging from slight differences in sample handling and preparation to instrument drift between runs. Although great pains are taken by operators to reduce these, small variations may still exist. To counteract this, I included a feature in flowBin to quantile normalize population markers.

Since tubes contain physical samples drawn from a common population, their true distributions in terms of population markers are expected to be identical, and any deviations to represent technical variation. Quantile normalization transforms two samples so that they have identical distributions, and has been used extensively in gene expression analysis [104]. FlowBin uses quantile normalization to bring similar cells into good registration, using the quantile normalization implementation from the Limma Bioconductor package [105]. The Limma implementation is capable of normalising in the presence of missing values, and hence can normalize data where the number of cells per tube varies.

4.2.2 Binning of Population Markers

In order to bin cells in terms of the overlapping markers present in all tubes, flowBin provides two methods: K-means clustering and probability binning. K-means clustering with a high value for K and nearest-neighbour joining has been used successfully in the past for identifying cell populations in flow cytometry data [43]. Probability binning is a binary space partitioning method for flow cytometry data in which each partitioning step maintains equal probability density within both partitions created [45]. Probability binning has been developed for use as a “micro-gating” algorithm in the form of the flowFP Bioconductor package [29]. Either method is typically used to partition the cells in a sample into bins containing enough cells to be able to extract average expression values; in our examples we chose this to be around 200 cells per bin.

4.2.3 Bin Matching Across Tubes

To enable expression to be combined accurately across tubes, the bins must be spatially as close to identical as possible in each tube. This is easier for flowFP, since the partitions have linear edges, which can easily be mapped directly to individual tubes. K-means, by contrast, produces approximately spherical clusters, with more difficult to describe boundaries. Rather than attempt to extract the boundaries of K-means clusters, flowBin draws on the idea of nearest-neighbour mapping, except that bin labels are mapped, rather than cellular identity. FlowBin K-means clusters the first tube in the set, and then for each subsequent tube, labels each cell according to the label of the closest cell in the first tube. Once all the cells in each tube have been assigned to cross-tube bins, flowBin moves on to calculating the expression of each bin in terms of the tube-specific expression markers.

4.2.4 Expression Measurement

FlowBin provides two methods for determining expression in each bin, modelled on common practice by flow cytometry analysts. Firstly, normalized median fluorescent intensity (MFI) can be computed (by subtracting the untransformed MFI in terms of negative control from that of the expression marker). Secondly, a threshold may be set at the 98th percentile of the negative control, and the proportion of cells exceeding that threshold in the expression marker channel reported, as is common practice in many studies [106–108].

4.2.5 Downstream Analysis

The final output of flowBin is a high-dimensional matrix of expression values for each bin (Fig. 4.1). This can provide a useful overview of the makeup of a sample, for example by plotting a heatmap of bin expression values. However, far greater utility comes in the downstream analysis methods that this enables. FlowBin output can be treated as though it were FCM data with a relatively low number of events but a high number of

markers. Then, methods for deep profiling, such as flowType and RchyOptimyx, can be applied.

4.3 Methods

4.3.1 Validation of Quantile Normalization

To validate flowBin's quantile normalization, I used an acute myeloid leukemia (AML) data set (Flow Repository:FR-FCM-ZZYA) [5]. This data set consists of 359 samples, with 8 tubes each, with 6 markers assayed in each tube. Every tube had an assay for CD45 as well as forward- and side-scattered light. flowBin's normalization was evaluated by applying it to each of these markers.

4.3.2 Comparison of Binning Methods

To aid users in choosing between the two binning methods flowBin provides, I compared them on a representative sample from the same AML data set as for the quantile normalization (Flow Repository:FR-FCM-ZZY). Figure 4.3 shows both the general form of the bins, and the distribution of cells across bins for comparison.

4.3.3 Comparison to Per-cell Nearest-neighbours

To compare flowBin to the per-cell nearest-neighbour merging of Pedreira et al., I created a small, synthetic example using real data containing peripheral blood mononuclear cells stained for CD3, CD4 and CD8. For the source data, I used data from the United States Military HIV Natural History Study (FlowRepository:FR-FCM-ZZZK). I first removed doublets, debris, dead cells and monocytes, as per [3], leaving only CD14$^-$ live cells. I then created two artificial tubes by randomly sampling two sets of 5,000 cells from the original sample.

4.3.4 Validation on Simulated Multitube Data from Polychromatic Flow Cytometry

To assess the abilities and limitations of flowBin, I again took data from the United States Military HIV Natural History Study (FlowRepository:FR-FCM-ZZZK). I preprocessed the data as per [3], screening out debris, doublets and non-viable cells, then finally gating for $CD3^+$ cells (T cells). Patients with fewer than 3,000 events remaining were removed, leaving 426 patients, with 12 fluorescent and two scatter channels.

To create simulated tubes, I chose CD3, CD4 and CD8 to use as common markers, then divided the remaining nine among three tubes. I divided the events for each patient randomly into three, and discarded all the markers for each that were not to be included in that tube. A summary of all the markers present in each tube is shown in Table 4.1.

I then ran flowBin on each patient's three tubes, using FSC, SSC, CD3, CD4 and CD8 as binning markers, with 128 bins and flowFP as the binning method. I ran flowType on the flowBin output (excluding CD3), and carried out survival analysis (Cox-PH and the logrank test) on the the flowType data as per [3]. I also ran flowType and the subsequent survival analysis on the original, full-colour flow cytometry data, again as per [3].

I compared the cell counts of individual cell types between the true counts from the flowType run on the original high-colour data, and the flowType run on the flowBin data, in terms of their Pearson correlation. I also compared the P-values of the logrank test for each cell type.

4.4 Results

4.4.1 Validation of Quantile Normalization

Figure 4.2 shows the results of applying quantile normalization to a single sample, in one, two and three dimensions. In a single dimension, all tubes are made to have identical cumulative distribution functions. This is as expected for quantile normalization. To ensure that the quantile normalization was improving the higher-dimensional registration

Table 4.1: Markers in simulated multitube data. The data was split into three tubes, each containing CD3, CD4 and CD8 in addition to FSC and SSC. The remaining nine markers were distributed across the tubes, three per tube.

**Marker Type**	**Tube 1**	**Tube 2**	**Tube 3**
Common (scatter)	FSC	FSC	FSC
Common (scatter)	SSC	SSC	SSC
Common (fluorescent)	CD3	CD3	CD3
Common (fluorescent)	CD4	CD4	CD4
Common (fluorescent)	CD8	CD8	CD8
Phenotyping (fluorescent)	KI67	CD57	CD27
Phenotyping (fluorescent)	CD28	CCR5	CCR7
Phenotyping (fluorescent)	CD45RO	CD19	CD127

of cells across tubes, and not introducing artefacts, two-dimensional scatter plots were qualitatively assessed, as shown in Figure 4.2. But to achieve a more objective, n-dimensional assessment of registration, I used cytometric fingerprinting [29]. This involved probability binning on all tubes pooled together, then measuring the degree to which the bin density in individual tubes varies from that of the pooled sample. In our example case, there was substantial deviation across tubes before normalization, which was almost completely removed after normalization.

To measure this objectively over the whole data set, I applied flowFP to measure the standard deviation before and after normalization for each tube of all 359 samples. Of a total 2,513 tubes, 2,207 (88%) showed improvement, 39 (1.6%) showed no change, and 267 (11%) showed a wider (worse) standard deviation following normalization.

4.4.2 Comparison of Binning Methods

In terms of form, K-means produces spheroid bins that are more likely to follow the contours of the underlying data. FlowFP, by contrast, produces bins with strictly horizontal-vertical borders, which are grid-like and less likely to follow the underlying contours of the data.

In terms of cluster density, flowFP (by the nature of its algorithm) produces bins with very even densities (SD of means = 0.07). By contrast, K-means produces a wide

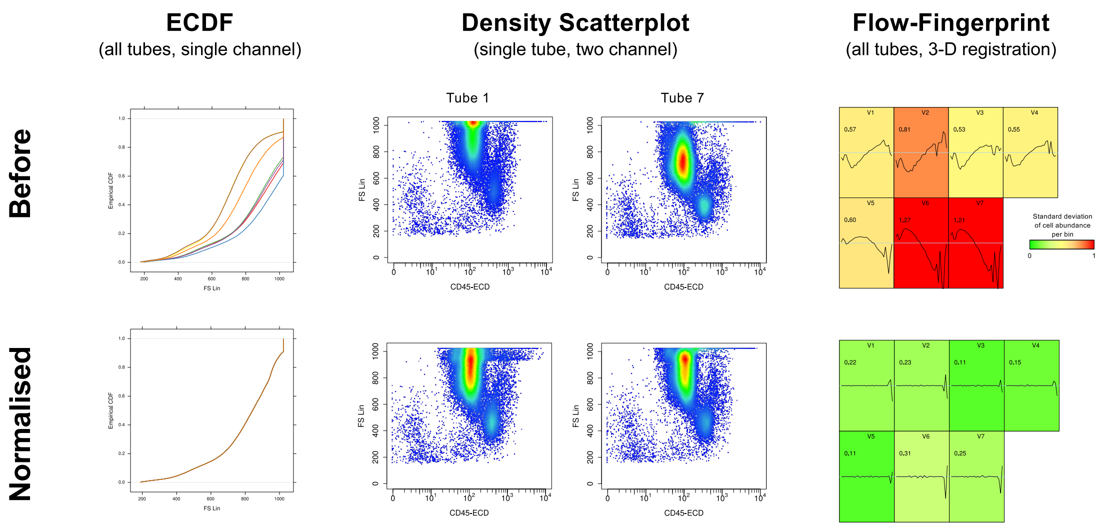

Figure 4.2: One, two and three-dimensional representations of quantile normalization of population markers. Empirical cumulative density function (ECDF) plots are shown for all tubes and for forward scatter (FS), the most variant marker. Following normalization, the ECDF for all tubes is identical, as is expected from quantile normalization. Two-dimensional scatter plots for representative tubes show visually the improvement in two-dimensional registration. Lastly, flowFP plots show the improvement in three-dimensional registration, measured by the standard deviation of the number of cells falling within each bin, after bins have been fitted to the consensus of all tubes.

range of bin densities (SD of means = 255), with the most dense bins containing as much as 20-fold as many cells as the least dense.

4.4.3 Comparison to Per-cell Nearest-neighbours

The results are presented in Figure 4.4, alongside the original data they were drawn from. The nearest-neighbours approach produced an artefactual CD4+CD8+ population, which is absent from the original data, and occurs only rarely in nature [109]. FlowBin, by contrast, creates a spectrum of values in a hyperbolic curve between the two “true” populations.

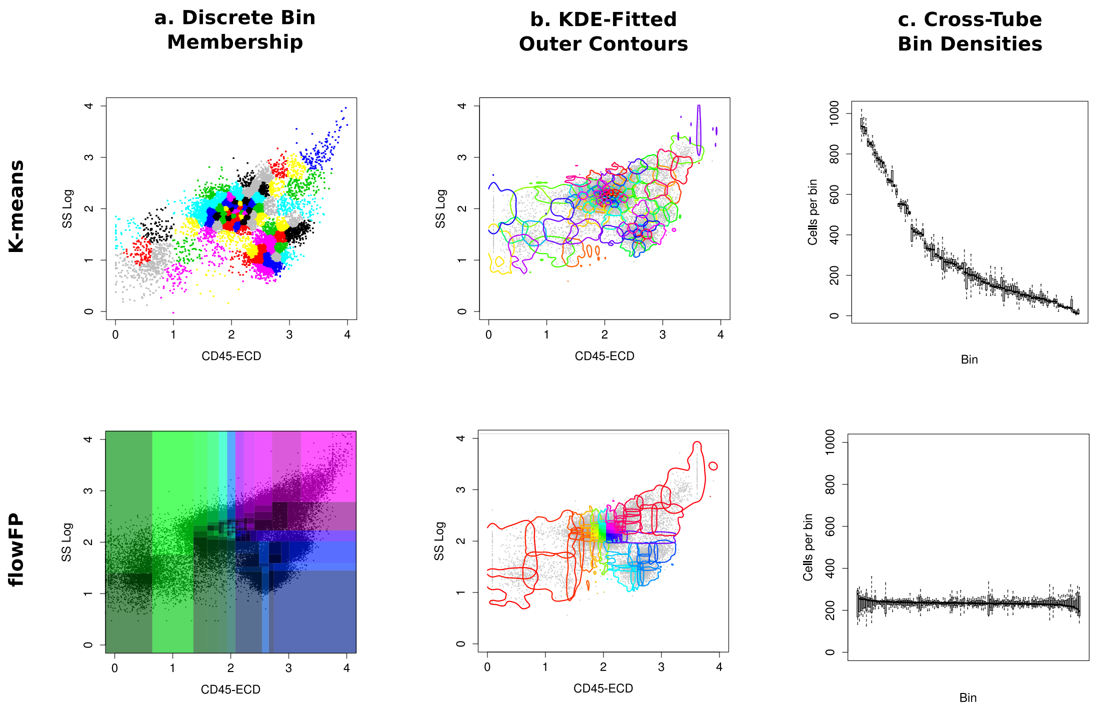

Figure 4.3: The two options for binning within flowBin: k-means and flowFP, as applied to a 7-tube sample. a. and b. show comparisons between the bin labels themselves. K-means creates roughly spherical bins, which conform around the location of cell populations. FlowFP creates grid-like bins,which may not conform to the true underlying shape of cell populations. c. shows the number of cells per bin across all tubes, for every bin. flowFP has approximately the same mean distribution of bin density across tubes as K-means (mean SD: 24.6 vs 28.5). However, flowFP has a much closer to constant number of cells per bin across bins (SD of means: 0.07 vs 255).

4.4.4 Validation on Simulated Multitube Data from Polychromatic Flow Cytometry

Running flowType with 11 markers and two partitions per marker gives a total of $3^{11} = 177,147$ possible cell types. I excluded those with 0% abundance across all patients, leaving $119,479$. Examining the three characteristic survival-associated cell types found in [3], more abundant cell types (especially KI-67$^+$CD127$^-$) appear to have better correlation, while rarer cell types (especially CD45RO$^+$CD8$^+$CCR5$^-$CD27$^+$CCR7$^-$CD127$^-$) have much poorer correlations (see Figure 4.5a). Importantly, the flowBin results for KI-67$^+$CD127$^-$ show a strong correlation with the true data, despite KI-67 and CD127 being in separate tubes. Looking across all cell types displays this same pattern (see

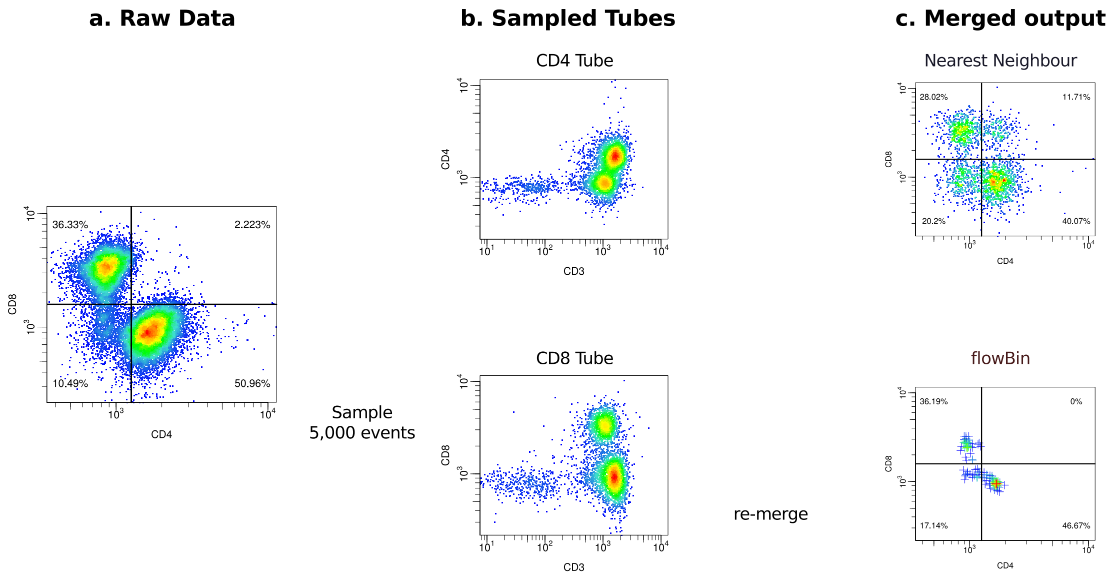

Figure 4.4: Comparison between nearest-neighbours merging and flowBin for two tubes computationally sampled from a real data set. a. Raw data (compensated, transformed and filtered for debris), gated for $CD3^+$ cells, and showing the true CD4 and CD8 distribution. b. The two sampled tubes, one containing CD4 and the other CD8. The $CD4^+$ population has slightly higher average CD3 than the $CD8^+$, but both have substantially overlapping CD3 distributions. c. Results of merging by nearest neighbours and by flowBin, including proportion of resulting "cells" falling within each quadrant. The nearest-neighbours merging created a substantial $CD4^+CD8^+$ population not present in the original sample. Both nearest neighbours and flowBin slightly overestimate the $CD4^-CD8^-$ population. FlowBin is more accurate at reproducing the $CD4^+CD8^-$ and $CD4^-CD8^+$ populations than nearest neighbours.

Figure 4.5b). Although some low-abundance cell types show strong correlations, it is likely that this was by chance, due to their having very low values in all patients. Since the flowBin results for the majority of cell types with a median abundance of 10% or more had a strong Pearson correlation with the true data, I chose to only do further analysis on those, leaving 1,896 cell types.

Comparing P-values, these showed a relatively good correlation ($R^2 = 0.65$), with the P-values resulting from flowBin being slightly higher than the true P-values (Figure 4.5c). Following Bonferroni correction, the cell types that were called as significant were matched between the true high-colour analysis and flowBin. Of the 592 cell types that would be called significant in the true data, only 58 were called significant by flowBin (9.8%). However, those 58 represent the majority of the 66 cell types that flowBin called

significant (88%).

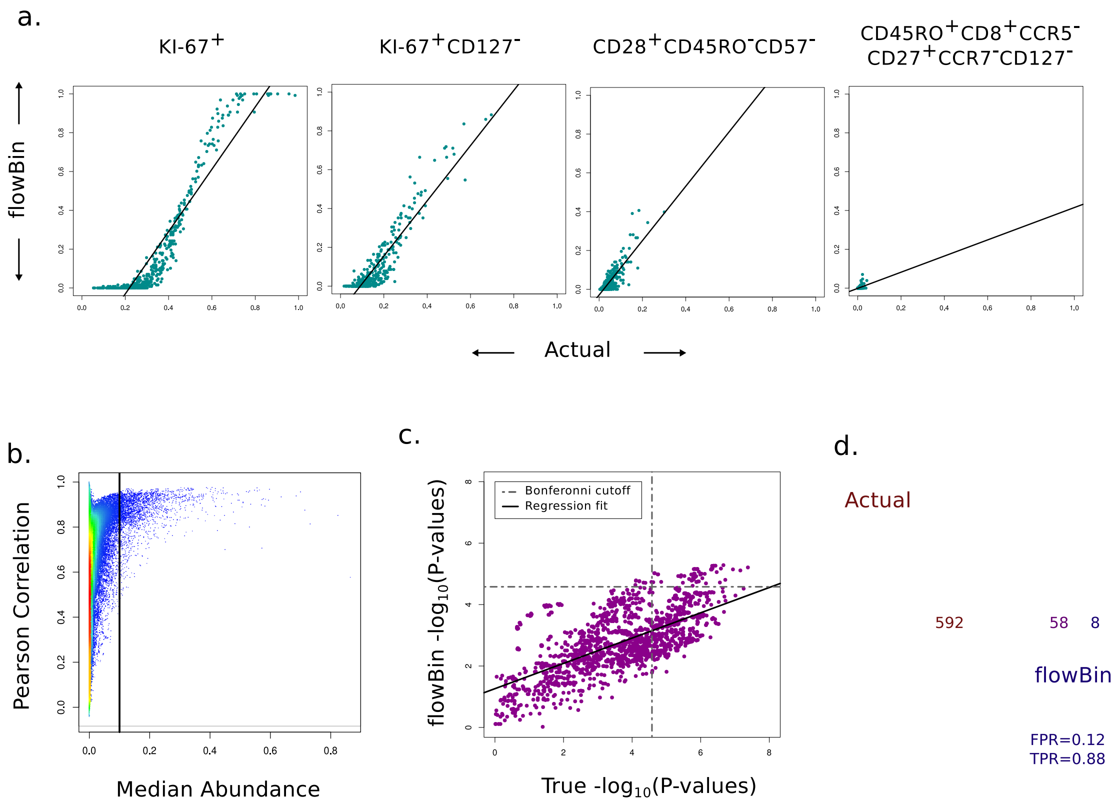

Figure 4.5: Performance on flowBin in reproducing a high-colour flow cytometry analysis on simulated flow cytometry data. a. Comparison of counts of selected phenotypes between actual data and simulated multitube data recombined by flowBin, with linear regression fit lines. Ki-67$^+$ was selected as being representative of a phenotype with the full range of abundance across patients. The remaining three phenotypes are the representative phenotypes of the three classes found in the original study [5]. More abundant phenotypes show a good, though imperfect fit. Less abundant phenotypes show a poorer fit. b. Pearson correlations for all phenotypes between actual values and flowBin-recombined values, vs median abundance of the phenotype. Below an abundance of approximately 0.1 (10% of all cells in the sample), the correlation becomes decoupled from abundance. c. Comparison of P-values between actual and flowBin-recombined data, for only those phenotypes with greater than 10% abundance. The P-values show a good correlation ($R^2 = 0.65$). d. Cell types called as being significant on flowBin-recombined simulated multitube data (blue) and the raw data (purple). Just less than 10% of the phenotypes that were called as significant in the actual data were also called as such in the flowBin-processed data (high type II error). Eight phenotypes were inappropriately called as significant (very low type I error).

4.5 Discussion

4.5.1 Validation of Quantile Normalization

The results of the comparison between non-normalized and normalized data suggest that quantile normalization can improve the registration of cells in terms of their population markers. This suggests that it should be considered before applying tube combination methods, including flowBin.

4.5.2 Comparison of Binning Methods

For binning, flowFP gives much less numerically dispersed results than K-means, which is essential for accurate flowType counts (Fig. 4.3). FlowFP binning may thus be a better choice for downstream applications that depend on accurate cell counts. For example, if flowFP is used for binning, the assumption can be made that each data point in the flowBin results has the same number of cells contained within it. If flowType is then applied to that data, the counts of cell types which flowType produces can be considered to be relatively accurate representations of the true counts.

K-means, by contrast, gives better fitted bins, but with greater variation. K-means binning thus may be a more attractive choice if later back-gating of interesting populations is desired. K-means may also be the only feasible choice in cases where there are very many population markers, as flowFP's binary space partitioning runs into combinatorial difficulties.

4.5.3 Comparison to Nearest Neighbours

The comparison (Fig. 4.4) showed that flowBin produced a distribution across quadrants and overall that more closely approximated the true distribution, while nearest-neighbours produced an artificial $CD4^{+}CD8^{+}$ population. The reasons for this spurious population are most likely as follows: Both CD4+ (helper) and CD8+ (cytotoxic) T cells express the T cell receptor, CD3. In other words, the CD3+ population contains a mixture of the two, randomly distributed within it. Consequently, when the nearest-neighbours approach

matches a CD3+ cell in the first tube to the cell with the nearest CD3 expression in the second, it is in effect sampling randomly from a mixed population. Since the ratio of CD4+ to CD8+ cells is roughly equal, about 50% of the time a helper T cell will be correctly matched with a helper T cell, and a cytotoxic with a cytotoxic. But for the other 50%, a helper T cell will be matched with a cytotoxic T cell, resulting in the combined cell being reported as either CD4-CD8- or CD4+CD8+, producing the spurious populations.

By contrast, flowBin samples groups of cells, and reports their average expression, in this case MFI. Thus most bins will contain a mixture of CD4+ and CD8+ cells, with the ratio varying in a Gaussian manner around the mean. Since it is extremely unlikely that flowBin will sample 200 cells of opposing types from each tube in the same bin, flowBin is much more robust against producing these kinds of spurious results.

This suggests that flowBin is a better choice for situations where it is desirable to recover the underlying cell types accurately, such as cell type discovery. Nearest-neighbours merging, due to its tendency to produce spurious combinations of marker expression, is poorly suited to techniques which require precise counts of particular cell types, such as flowType, or manual analysis. However, nearest-neighbours has proven extremely effective when used as part of a classification pipeline [38, 76], whereas flowBin loses some information as a result of averaging. As such, the two methods can be complementary to each other.

4.5.4 Validation on Simulated Multitube Data from Polychromatic Flow Cytometry

When compared with simulated multitube data in a complete analysis pipeline with flowType, flowBin reproduced the true underlying trends in the data, but with lowered statistical power and lowered sensitivity. The individual correlations (Fig 4.5a and b) suggest that cell type counts based on flowBin recombination of tubes reproduces the true counts for most cell types of greater than 10% abundance. This suggests that while

flowBin may not be suitable for analysis efforts examining rare cell populations (such as minimal residual disease in leukemia or T cell subsets), it is a useful tool for examining more abundant cell types (for example the gross heterogeneity among tumour types).

For reproducing the results over the entire pipeline, flowBin performed well once cell types of less than 10% average abundance were filtered out. P-values of the survival analysis were close to those of the pipeline run on the true data, but slightly raised. This resulted in increased type II error, but minimal type I error. This suggests that while flowBin introduces some noise, the final effect is only to lower the statistical sensitivity of analyses performed on flowBin expression data, but not to produce false positives.

Interestingly, KI-67$^+$ showed a slightly sigmoidal distribution in Fig 4.5a. The likely reason for this is because flowBin produces averages, while flowType uses a threshold (see Fig 4.4c). Thus, bins with fewer positive cells will tend to have an average (flowBin) expression which falls below the flowType threshold, This would also depend on the distribution of the marker across cells in the common markers. A marker that was completely uniformly distributed in every patient would tend to have a curve approaching a step function. In that situation, for a sample with 49% expression of that marker, the expression of every bin would fall just short of the flowType threshold and the sample would erroneously appear to have 0% expression.

It may be an avenue for future investigation to attempt to mitigate this, for example by applying a logit transformation to flowBin expression data before passing it to flowType. However, fitting logit transforms would be challenging for most cell types, as their distributions would be a mixture of the sigmoidal distributions of their component markers. Indeed, for KI-67$^+$CD127$^-$ in Fig 4.5a, the sigmoidal curve is partially cancelled out, most likely by such mixing.

4.5.5 Conclusions

FlowBin is a complete pipeline for combining multitube flow cytometry data via markers shared across tubes. Quantile normalization of those markers to reduce technical variation

is included. Of the two forms of binning included, flowFP is most suitable for later flowType analysis, while K-means fits the contours of the data better. Compared with nearest-neighbours merging of tubes, flowBin produces cleaner data, with far fewer false double-positive marker combinations. FlowBin with flowType can reproduce true data for more abundant cell types, and this data is suitable for statistical testing, albeit with lowered statistical power (increased type II error).

Chapter 5

Applications of FlowBin to AML

5.1 Introduction

As mentioned in Chapter 2, multitube flow cytometry data is common in leukemia diagnosis. FlowBin is thus ideally placed to be used for high-throughput deep profiling of AML data. In this chapter, I detail several AML datasets to which I applied flowBin.

5.1.1 Separation of AML and Normal Cells

It is frequently desirable to isolate dysplased cells from healthy tissue in order to characterize the dysplasia; I demonstrate here how flowBin can be used to achieve this. Once again I used the multitube AML data set from FlowCap-II (FR-FCM-ZZYA). This data set contains FCS files for 359 patients (normal=316, AML=43). Theoretically, the samples from the AML patients should contain a mixture of normal and leukemic blast cells, while the healthy patient samples should only contain normal cells. The problem of separating abnormal from normal cells is thus one of novelty detection, for which techniques, such as single-class support vector machines, are available [110].

5.1.2 FlowCAP2

Flow Cytometry: Critical Assessment of Population Identification Methods (FlowCAP) is a competition in which automated flow cytometry analysis methods are compared. The second FlowCAP challenge compared classification pipelines, and one data set included multitube data for AML.[5] I entered a pipeline using flowBin for feature extraction along with a voting classifier method.

5.1.3 flowBin with flowType and RchyOptimyx to find cell types in AML correlated with *NPM1* mutation

To further demonstrate the utility of flowBin, I present its application to a novel data set. The data in questions consists of 129 *de novo* AML cases. Each of these cases had multitube flow cytometry data available from the time of diagnosis. In addition, each had been genotyped for clinically relevant frameshift mutations in the twelfth exon of the *NPM1* gene. These mutations (hereafter referred to as *NPM1-mt*) indicate a good prognosis, and have a marked correlation with the absence of CD34 on the AML blast cells [64, 65, 67, 111] and, in certain cases, HLA-DR [112].

While high-level, single marker studies have been performed to find other recurrent immunophenotypic characteristics of *NPM1-mt* AML [112, 113], deep profiling of the condition's immunophenotypic landscape has yet to be undertaken. I performed such an analysis using flowBin and flowType, with the hypothesis that within the immunophenotypic landscape of AML, there may be additional cell types that are more strongly correlated with *NPM1* mutation than CD34+/- alone.

5.1.4 Surveying the Immunophenotypic and Genomic Landscape of AML

As detailed in Chapter 2, AML genomes, transcriptomes and various other sequence-based data has been published, but a comprehensive bioinformatic survey of the immunophenotypic profiles of AML based on flow cytometry had not until the work I described in the previous section. Furthermore, a study integrating the full depth of flow cytometry data with sequence-based data has not been undertaken at all. In this section I describe such work.

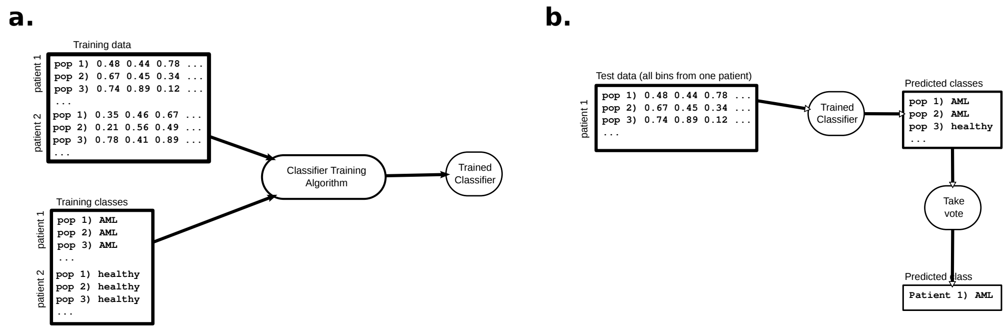

Figure 5.1: Schema for a voting classifier using flowBin output. a. Training. Every bin from every patient is treated as an individual measurement, labelled with the class of the patient. A classifier is then trained on the entire dataset at once (all bins from all patients). **b. Prediction** To predict the class of a new patient, a prediction is made by the trained classifier for every one of the bins from the patient. The majority vote from these predictions is then taken as the overall prediction for the patient.

5.2 Methods

5.2.1 Separation of AML and Normal Cells

I applied flowBin to both the normal and AML patients using K-means binning. I pooled all bins from the normal samples, and trained a single-class support vector machine (SVM) on these using the kernlab CRAN package [114]. I then applied the trained SVM to the bins from the AML patients to predict which were normal and which dysplased.

5.2.2 FlowCAP2

Binning within patients raised the problem of linking features across patients for classification. To solve this, I took each bin from each sample as a separate training instance, labelled with the sample label, and then trained an SVM classifier. For class prediction, I took the majority vote of the predicted labels for a given sample's bins. Classification with parameter optimization and three-fold cross-validation was implemented using the ksvm R package, but could in theory be made to work with any modern classification method. This method is illustrated in Fig 5.1.

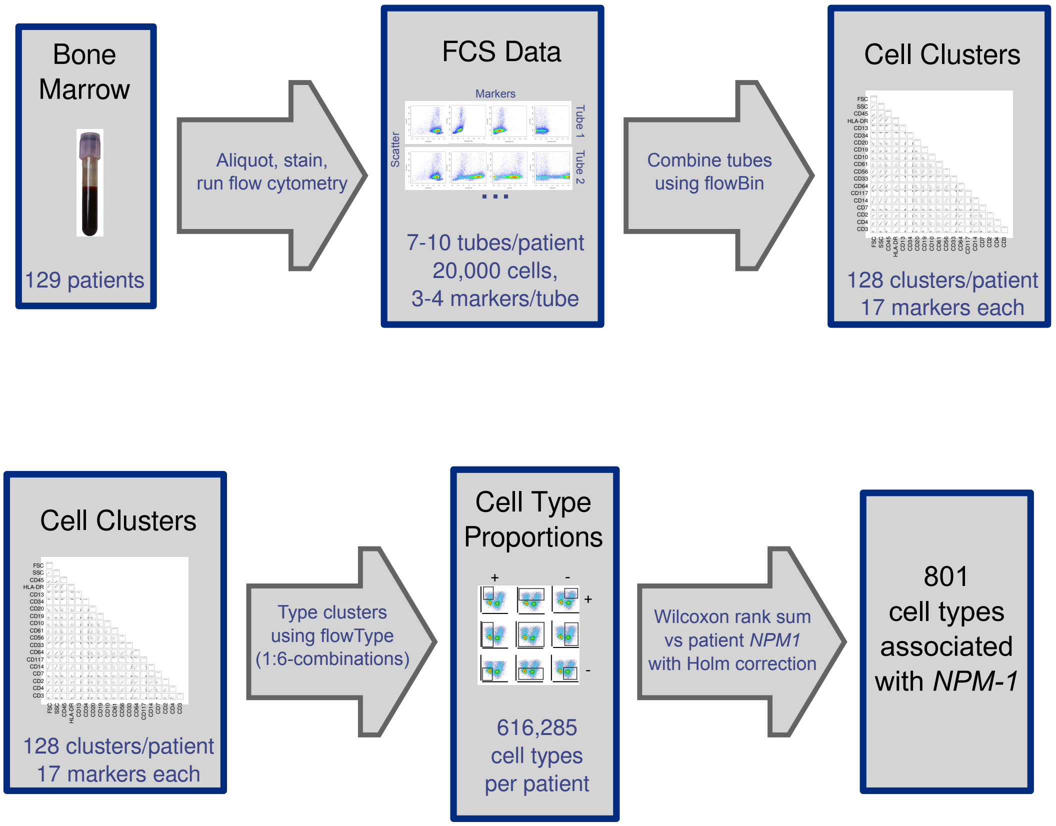

Figure 5.2: Pipeline used to determine *NPM1*-associated immunophenotypes in AML. Steps taken are denoted by arrows, while the data consumed/produced is indicated in boxes. Flow cytometry was performed in the clinic historically; all other steps were computational. The end result was a list of 801 cell types which showed a significant difference in abundance between *NPM1* mutated and wild-type patients.

5.2.3 FlowBin with FlowType and RchyOptimyx to Find Cell Types in AML Correlated with *NPM1* Mutation

I first used flowBin to combine tubes for each sample and measure the expression of the mapped bins. I then used flowType to delineate and count all cell types present, defined over all combinations of up to six markers. I filtered out all cell types not present in any patient, leaving 616,285. I then tested for differences in abundance of each of these cell types between *NPM1-mt* and wild-type patients using the Mann-Whitney U test with Bonferroni-Holm correction for multiple testing. These steps are illustrated in Fig 5.2.

I then performed exploratory analysis using RchyOptimyx to visualize the hierarchies

within the 801 significant cell types. I found that adding CD19-, CD20- and CD10- had little to no effect on the P-value of a cell type (see Fig 5.3). This is likely due to these (B-lymphoid) markers being extremely rare in AML [54], and hence not being expressed on the important cell types at all, so that a negative gate for any of them did not change which cells had that cell type. I consequently excluded all cell types involving CD19-, CD20- or CD10-, bringing the total cell types to 272.

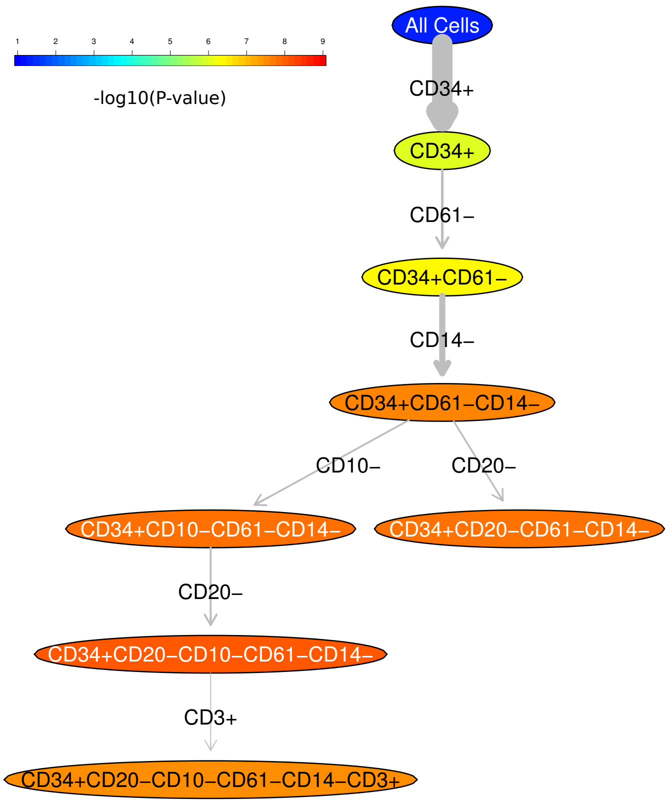

Figure 5.3: An example of RchyOptimyx analysis of one cluster of cell types. As 801 cell types are too many to visualize meaningfully with RchyOptimyx, I clustered the cell types and visualized each in turn. In this example, the addition of CD10- or CD20- make little difference to the P-value of the cell type $CD34^{+}CD61^{-}CD14^{-}$. As this was a general trend and in line with reported AML biology, I chose to exclude cell types defined over these markers from further analysis.

5.2.4 Surveying the Immunophenotypic and Genomic Landscape of AML

List mode data from clinical flow cytometry performed at diagnosis of 93 patients was obtained retrospectively. This data contained a common panel of 16 markers spanned across multiple tubes, with each tube containing CD45 to delineate leukemic and white blood cell populations. I combined tubes using flowBin, with the flowFP method for binning, 128 bins, and percentage positive as the measure of per-bin expression. I then used flowType [2, 3] to extract and count all phenotypes defined over combinations of up to four markers, with three levels of expression (negative: $< 20\%$ markers expression; partially positive: between 20% and 80% marker expression; fully positive: $> 80\%$ marker expression). Of the 58,742 phenotypes, I excluded those with a mean cell count of $< 5\%$ of all cells and a standard deviation across cell counts of $< 10\%$ of all cells (based on examination of the distribution of these values), leaving 4,938.

I then clustered the remaining cell types using non-negative matrix factorisation, as implemented in the NMF bioconductor package [115], in a manner similar that used for mRNA and miRNA in [72]. I used the default Brunet algorithm, with 50 and 200 iterations respectively for the rank survey and clustering runs. The number of clusters was chosen based on cophenetic score and silhouette width. I selected the top ten cell types from each cluster, and plotted the cluster results on a heatmap of these cell types against genotype and disease subtype.

To determine which mutations were significantly correlated with immunophenotypic clusters, I used a two-tailed Fisher's exact test between cluster membership and presence of the mutation, followed by the Bonferonni-Holm correction.

5.3 Results

5.3.1 Separation of AML and Normal Cells

Heatmaps of the two predicted classes of bins are shown in Figure 5.4. The bins predicted to be normal fall mainly into well-clustered populations showing expression patterns typical of healthy myeloid (monocytes, promonocytes and granulocytes), lymphoid and erythroid cells respectively. By contrast, the bins predicted to be abnormal show many more clusters, and very few bins which fit normal expression patterns. Most of the bins show patterns of expression more typical of AML, such as CD34 and CD117 expression, and co-expression of the CD4 and CD7 with myeloid markers.

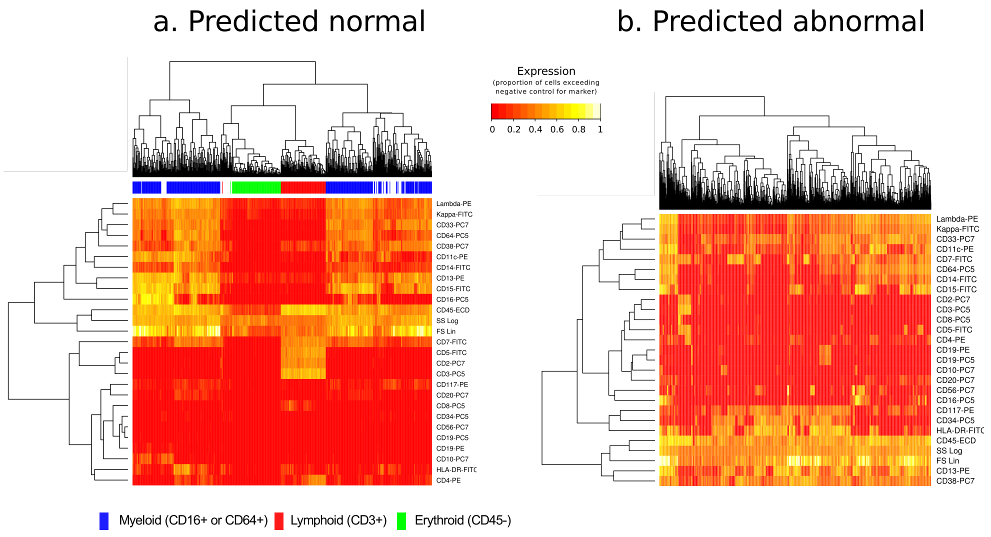

Figure 5.4: nu-SVM separation of normal and abnormal cell populations in AML samples. a. Heatmap of all populations within the AML samples that were predicted to be normal. Most can readily be identified as having the properties of common blood and bone marrow cell populations: myeloid cells expressing CD16 and/or CD64, lymphoid cells (dominated by CD3-expressing T-lymphocytes/precursors, and erythroid cells not expressing any of the markers in the panel, including CD45. b. Heatmap of all populations predicted to be abnormal. In contrast to the cells predicted to be normal, many of these express CD34 and CD117, primitive markers typical of stem cells and of AML.

5.3.2 FlowCAP2

This pipeline performed poorly in comparison with other algorithms, with an F-measure of 0.46.[5] Although the competition included an opportunity for participants to improve their scores, an error in the score-calculating code initially showed my pipeline as having a much higher F-measure during that phase, and so I did not have a chance to improve the pipeline until after publication. In dissecting this performance, it emerged that the classifier was vulnerable to class imbalance (only 43 of the 359 patients had AML). To address this issue, I added bagging with down-sampling to the classifier [116]. This improved classifier, illustrated in Fig 5.5, produced an F-measure of 0.96 when evaluated using the same methodology as was used in FlowCAP.

5.3.3 FlowBin with FlowType and RchyOptimyx to Find Cell Types in AML Correlated with *NPM1* Mutation

The final set of 272 *NPM1*-associated cell types, after removing those defined over uninvolved markers, are illustrated on a heatmap showing their abundance in Figure 5.6. The cell types clustered into two main groups, roughly corresponding to the two patient classes (*NPM1-wt* and *NPM1-mt*). Relative abundance of cell types with stronger P-values, chosen by exploration using RchyOptimyx, are shown in Figure 5.7.

5.3.4 Surveying the Immunophenotypic and Genomic Landscape of AML

The results of the NMF clustering are summarized in Fig 5.8. The only mutations showing significant correlations with the immunophenotypic clusters were those in *NPM1* (P=0.0006) and its closely-associated mutations in *DNMT3A* (P=0.0014) [117].

However, certain combinations of other class II mutations (those which block differentiation [118, 119]) clearly account for some clusters. In clusters 4 and 8, most patients have *NPM1* mutations, but those which do not have t(9;11) karyotype or mutations in *TET2*. In cluster 3, the two patients which do not have *NPM1* mutations

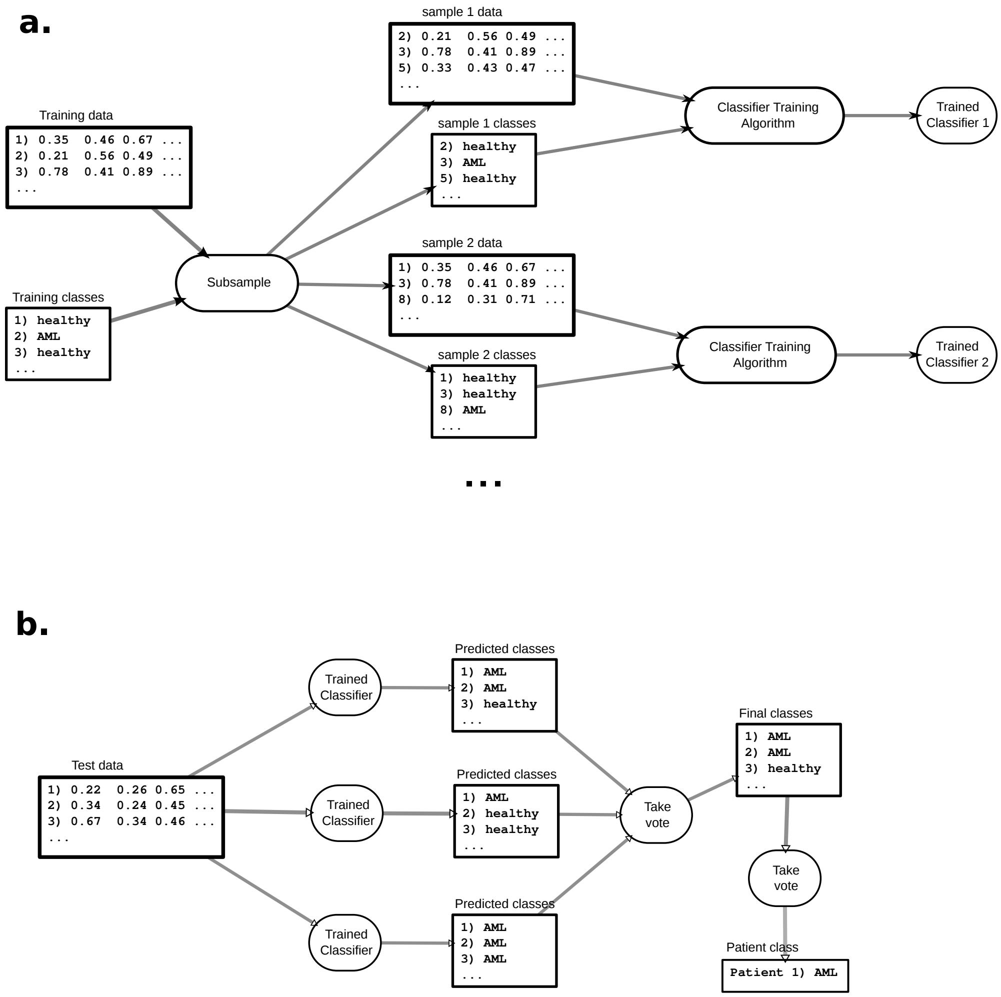

Figure 5.5: Schema for a voting classifier for flowBin output incorporating balanced bagging. a. Training. This is similar to the base classifier shown in Fig 5.1, except that multiple classifiers are trained, each on a bootstrap subsample of patients. Each bootstrap sample is set to contain equal numbers of patients from each class. **b. Prediction** To predict the class of a new patient, predictions for each bin from that patient are made by each of the trained classifiers. Final per-bin predictions are taken by majority vote of those predictions. Then, the prediction for the patient is made based on a majority vote of the per-bin predictions.

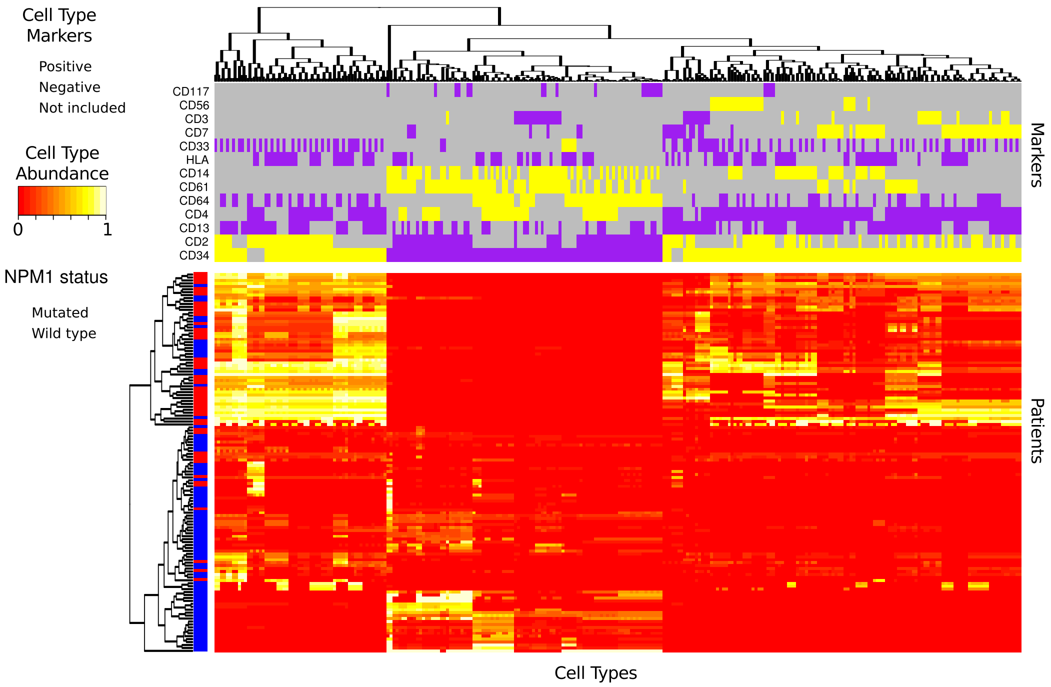

Figure 5.6: Overview of cell types which showed significant differences in abundance between *NPM1-mt* and *NPM1-wt*. The cell types cluster into two main groups, roughly corresponding to the two patient classes (*NPM1-wt* and *NPM1-mt*).

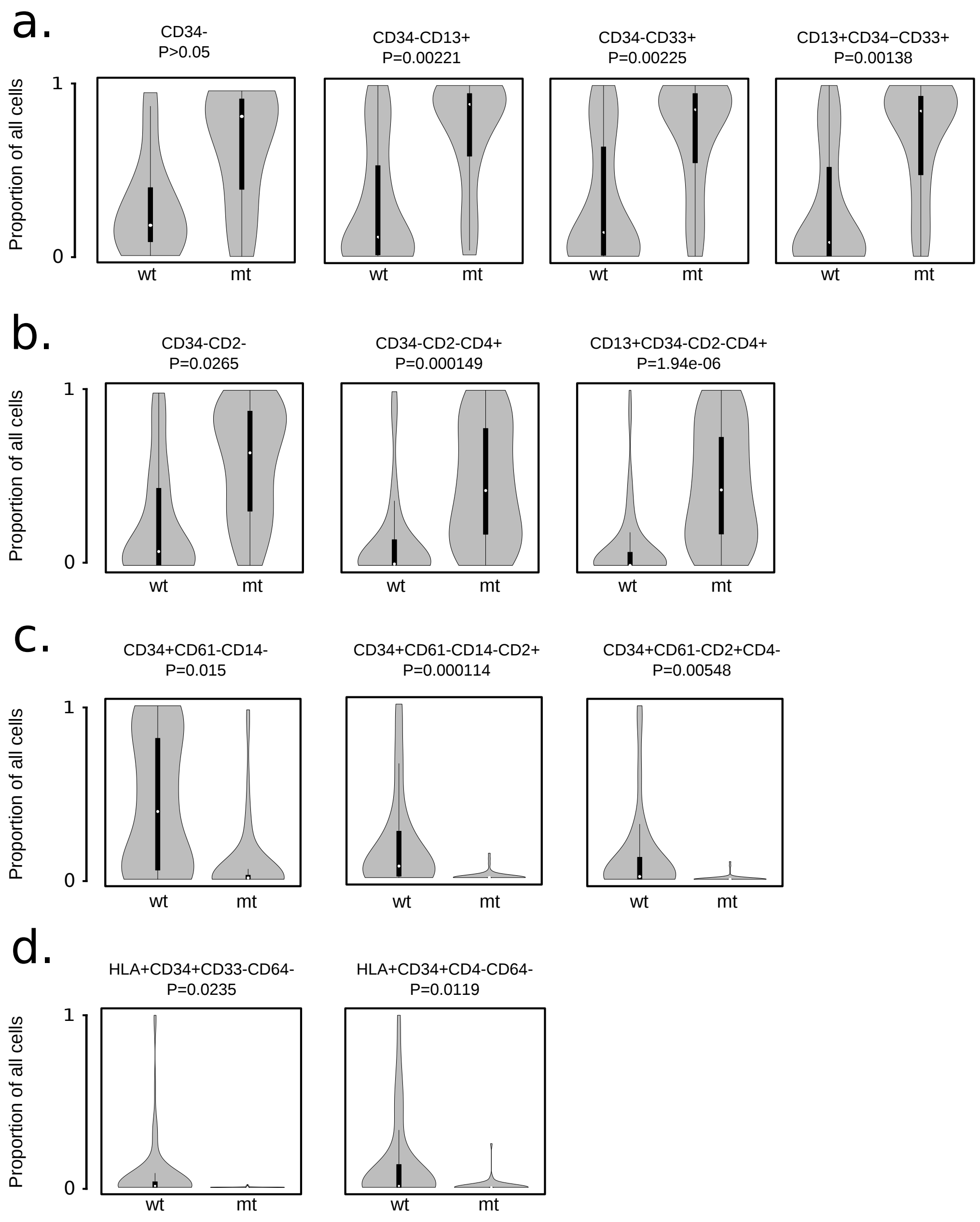

Figure 5.7: Selected classes of cell types showing significant differences in abundance between *NPM1-mt* and *NPM1-wt*. P-values are given after Holm correction. a. Gating for the presence of myeloid lineage markers CD13 and CD33 within the CD34- compartment yields much stronger differences in abundance between *NPM1-wt* and *NPM1-mt* than CD34- alone. b. Gating for CD2- within the CD34- compartment yields a slightly better separation than CD34- alone, but gating down further to CD4- and CD13+ is a cell type that, while present in most *NPM1-mt*, is absent or below 20% abundance in nearly all *NPM1-wt*. c. Gating for CD61- and CD14- within the CD34+ compartment leads to a cell type which is common in *NPM1-wt* but almost entirely absent in *NPM1-mt*. d. Gating for HLA-DR+ and CD64- within the CD34+ compartment leads to a cell type that occurs in a subset of *NPM1-wt* but is entirely absent in *NPM1-mt*.

Table 5.1: Mutations and mutation groups correlated with immunophenotype clusters by Fisher's exact test. Note that the cell types column only shows the cell type most strongly associated with the cluster(s) in question, and not the entire spectrum of cell types present or absent. For the full spectrum of cell types per cluster, see Figure 5.8

Mutation(s)	Cluster(s)	P	Cell Types
NPM1	3, 4, 8	0.0006	$CD34^{-}CD33^{hi}$
NPM1|*TET2*|t(9;11)|*PML/RARA*	3, 4, 5, 8	8.7×10^{-10}	$CD34^{-}CD33^{hi}$
DNMT3A	4	0.0014	$CD33^{hi}CD64^{hi}$
RUNXIT1/RUNX|*MYH11/CBFB*	6	0.0001	$CD34^{hi}CD117^{hi}$

have the *PML/RARA* fusion gene. Cluster 6 contains mainly patients with either the *RUNXIT1/RUNX* fusion gene, or the *MYH11/CBFB* fusion, and the unadjusted P-value for these two genes combined is 0.0001, as compared to 0.04 for *RUNXIT1/RUNX* alone. This cluster is characterized by having a high proportion of cells expressing CD34, CD117 and HLA-DR. When *TET2*, t(9;11) and *PML/RARA* are added to *NPM1*, the (unadjusted) P-value of the Fisher's exact test is 8.7×10^{-10}, as opposed to an unadjusted P-value of 3×10^{-5} for *NPM1* alone. Although *TET2* mutations are not confirmed to be class II mutations in AML, deletion of Tet2 in mice has shown to result in increased hematopoeitic stem cell proliferation, suggesting that they may be [120]. These clusters are defined by an absence of CD34 expression and a high proportion of cells expressing CD13.

5.4 Discussion

5.4.1 Separation of AML and Normal Cells

I have shown how flowBin in combination with one-class SVM can be useful for separating normal from aberrant cells where a good training set of normal cells is available. This can have applications for later AML studies where the ratio of normal cells to leukemic is a confounding factor.

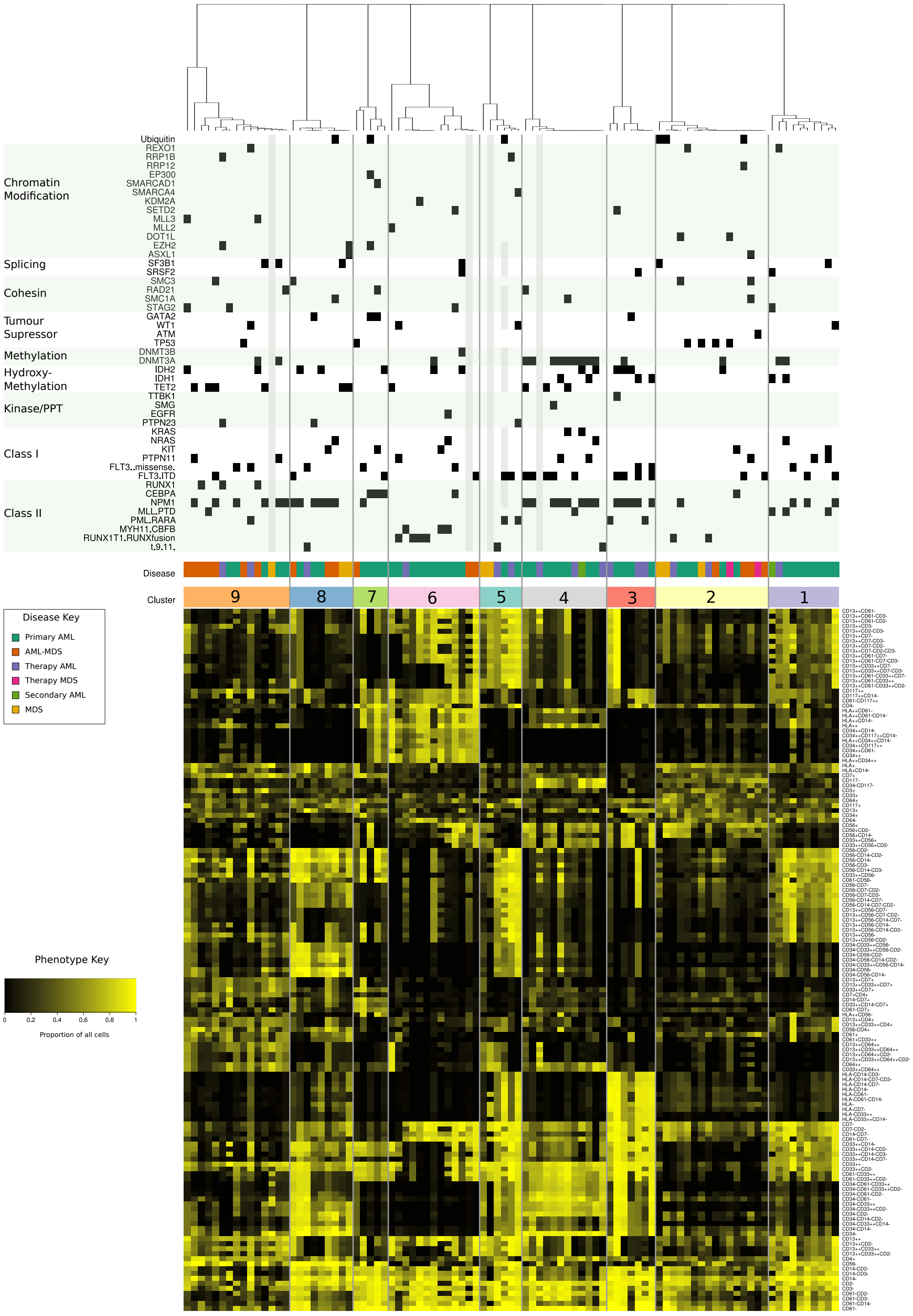

Figure 5.8: Top phenotypes and the results of NMF clustering. Phenotypes are indicated on the y axis of the heatmap; '-' indicates cell populations negative for the marker, '+' indicates partially positive, and '++' indicates fully positive. Patients, clustered by nonnegative matrix factorization, are indicated on the Y axis. Greyed out genotypes are those for which sequencing failed.

5.4.2 Identifying AML Patients (FlowCAP 2)

I have shown that, despite poor performance in the initial FlowCAP2 competition, a voting classifier used in conjunction with flowBin can separate AML from normal cells, when balanced bagging is added. On the AML data set from FlowCAP, balanced bagging improved performance substantially, with the F-measure increasing from 0.46 to 0.96, a number roughly in the middle of the field compared with the other pipelines entered. This could be improved further by incorporating other machine learning best practices, such as feature selection. However, there are two levels of recursion already: the voting classifier and the bagging. Adding another layer of recursion would likely increase the computational complexity prohibitively. Instead, it would be better to recommend that flowBin be used in conjunction with the best-performing techniques from FlowCAP, most notably flowType and SPADE. [5]

5.4.3 FlowBin with FlowType and RchyOptimyx to Find Cell Types in AML Correlated with *NPM1* Mutation

The overall pattern of association between CD34 expression and *NPM1* mutation shown in Figure 5.6 fits with previous reports.[65, 66, 111] However, flowBin with flowType was able to find a multitude of immunophenotypes within those classes (CD34$^+$ and CD34$^-$).

Referring to the groups examined in more detail in Figure 5.7, the first group, CD34-CD13+CD33+, fits with observations that blasts often express CD13 and CD33 in *NPM1-mt* AML [54]. The second and third groups, involving CD34-CD2- and CD34+CD2+, are both cell types which have been reported before in Acute Promyelocytic Leukemia [121], but not associated with *NPM1*. For the cell types in the second and third groups, CD4 has been recently reported to be associated with *NPM1-mt*, along with t(9;11) and monocytoid AML [76]. In the third group, CD61, a marker of megakaryoblasts, may fit with the observation of dysmegakaryopoeisis in *NPM1-mt* AML [122].

In conclusion, I have found a series of cell types associated with *NPM1* mutation

in AML. Although many of these cell types fit with previously reported trends, most represent new, previously unreported cell types associated with *NPM1* mutation. Importantly, I have also demonstrated that flowBin can be used to produce high-dimensional data from low-dimensional multitube flow, suitable for downstream analysis in tools such as flowType and RchyOptimyx the identify significant cell populations.

5.4.4 Surveying the Immunophenotypic and Genomic Landscape of AML

I have shown how flowBin and flowType can be used, with NMF clustering, to find AML patients with similar sets of cellular immunophenotypes. I have further shown how this can be used as an alternative method to find genes (or combinations of genes) with characteristic immunophenotypes. Interestingly, many of the mutation types showing distinctive immunophenotypes are those with more favourable outcomes, while the known mutations with less favourable outcomes tended to be less associated with a single immunophenotype.

Chapter 6

BAIT: Automated Analysis of strand-seq Data for Detection of Sister Chromatid Exchanges and Genome Finishing

6.1 Background

As described in Chapter 2, strand-seq is a powerful technique which enables the detection of SCEs. However, the method as published in [83] involved visualising binned read counts on chromosome ideograms, then manually identifying change points between WW, WC and CC states. Like most visually based manual analysis, this is both time-consuming and prone to bias. Automating this analysis was thus a desirable goal, and in this chapter I describe a method for doing so, which I developed in collaboration with Mark Hills.

Furthermore, once SCEs can be systematically identified in large numbers of cells from the same organism or species, they can be used for a range of purposes. One such is finding and correcting misoriented contigs flanked by gaps within late-build genomes [83]. This can be extended to placing unbridged contigs in a late-build genome by their strand similarity to chromosomal regions across libraries. While this could be performed by manual inspection, that would again be tedious and potentially subjective, and an automated method was desirable. Later in this chapter, I describe a method developed

by Mark and myself to place unbridged contigs based on strand similarity. This method depends upon the accurate detection of SCEs. Both methods have been incorporated into a package called bioinformatic analysis of inherited templates (BAIT), which is available on Sourceforge.

6.2 Methodology

6.2.1 Automatically Detecting Sister Chromatid Exchanges

The sister chromatid exchange detection in BAIT accepts the binned read depths as produced for the visualisation. Although the overall read depth is unchanged across an SCE, the proportion of directional reads will change from two copies in the homozygous state to one in the heterozygous state (see Figure 2.11 in Chapter 2). BAIT exploits the similarity of the change in template copy number to copy number variation (CNV) analysis in order to locate and characterize all SCE events.

To create a single track of data for CNV, and to normalise for read depth differences, the ratio of Watson and Crick reads within each bin is computed, using $[(W-C)/(W+C)]$. This gives a value of 1 when all reads map to the Watson strand (WW strand inheritance), -1 when all reads map to the Crick strand (CC), and 0 for an equal number of both (WC) (Fig 6.1a). A change in this ratio along the length of a chromosome corresponds to the location of an SCE event (Fig 6.1a), which is first localized to neighboring bins. For example, using the default bin size of 200 kb, a switch from a CC template-strand state in one bin (ratio = -1) to a WC template-strand state in a neighboring bin (ratio = 0) indicates that an SCE event occurred somewhere within the 400 kb interval encompassing those two bins (Fig 6.1a).

A bin size of 200 kb was selected to be large enough that, even in low read depth strand-seq data, such as that from an Illumina MiSeq, the read depth over that interval should, in our experience, be adequate to localise an SCE within the interval. BAIT first makes gross event calls by utilizing the circular binary segmentation algorithm [123]

implemented in the CNV Bioconductor package DNAcopy [124] to locate the SCE event to the two-bin interval. It then recalculates the template-strand ratio by segmenting this interval into five new bins (80 kb each, using default bin size), further narrowing the location of the SCE interval . BAIT applies this binning-based DNA copy detection method iteratively, decreasing the bin size by a factor of five each time (Fig 6.1b), until the read density is no longer sufficient to make accurate calls (determined from experience to be when an interval has less than 50 reads, or when DNAcopy can no longer predict a single event (Fig 6.1c). In order to identify SCE events on the boundary of bins, BAIT pads each interval with one-half of the interval length in each direction (Fig 6.1b, c; red arrows).

BAIT then refines the gross interval by incorporating a simple walker algorithm that analyzes reads starting from the homozygous state, and reports the first read on the opposite template that represents a switch to a heterozygous state (Fig 6.1, red box). From this refined interval, the walker checks that the 10 preceding reads map to the homozygous state, and that at least four of the 20 following reads map to the opposite template state (Fig 6.1c). If these criteria are not met, as may be the case where the background is high, BAIT continues to analyze across the interval until they are met. These checks improved the localization of SCE events (see Fig 6.2), and varying these thresholds did little to change the data. Through this two-step process, BAIT automatically detects and localizes SCEs with a high degree of confidence, plots them on ideograms, and creates a UCSC-formatted BED file of all SCE event intervals.

6.2.2 Placing Contigs in Late-build Genomes Using Strand Inheritance and Sister Chromatid Exchange Information

Using scaffold-level and chromosome-level assemblies to generate functional reference assemblies is valuable, but it is important to note that 'completed' assemblies also contain a large number of contigs that remain unmapped. Assigning locations for these orphan scaffolds in a chromosome context is a high-priority endeavor for sequencing

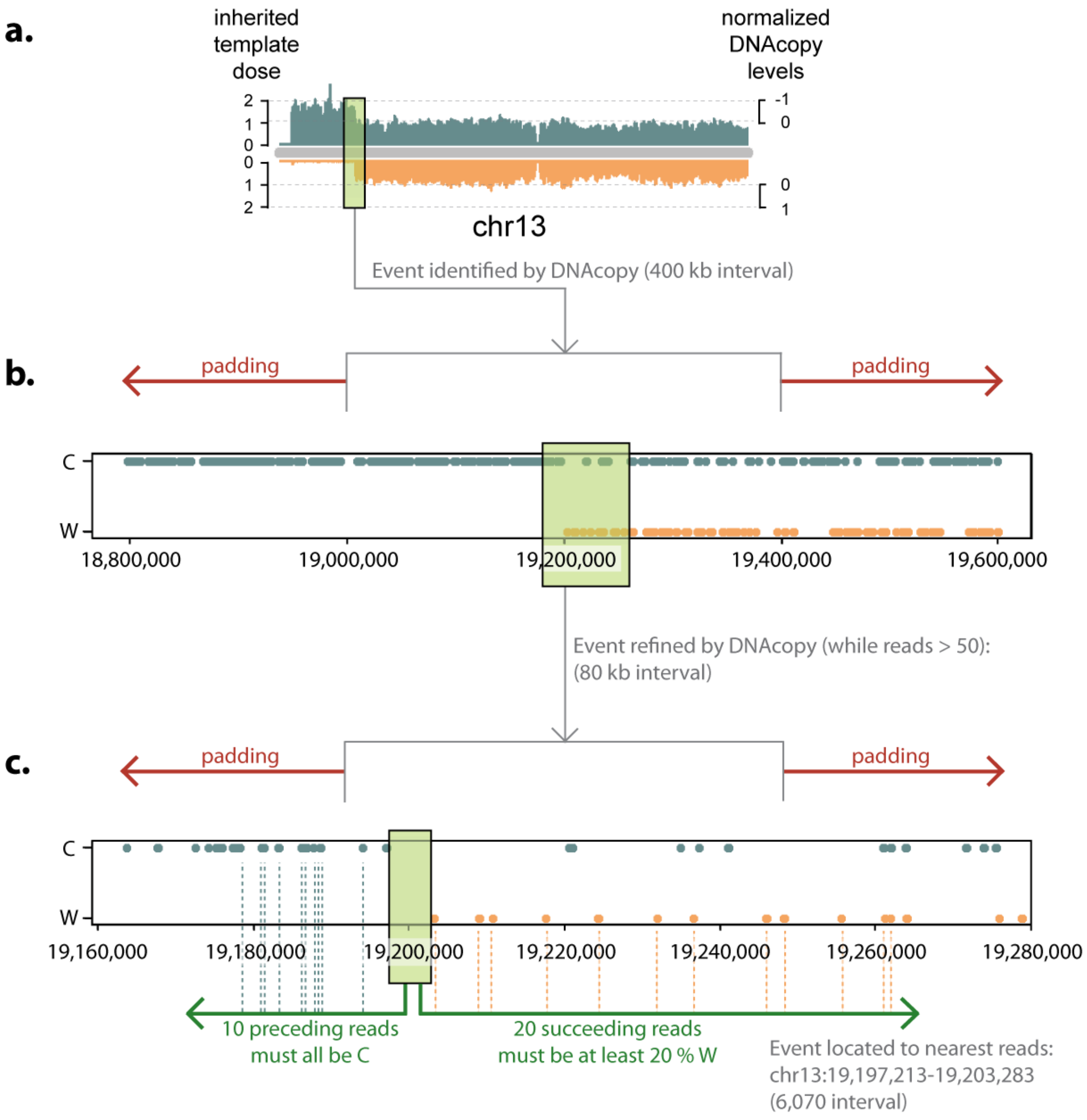

Figure 6.1: Automated identification of sister chromatid exchange (SCE) from strand-seq data. (a) Gross directional mapping data are thresholded to remove bins with unexpectedly high or low read numbers, and analyzed using DNAcopy. Inherited template numbers are converted to a value between 1 and -1 for DNAcopy to make only one of three calls: WW, WC, or CC. DNAcopy defines an interval across two bins, so with a bin size set to 200 kb, the SCE event will be located to within 400 kb. **(b)** Localization is then iterated by subdividing the identified region into bins one-fifth of the original size (80 kb on first iteration), and re-running DNAcopy. A single bin size is used as padding to aid detection of SCE events at bin boundaries. The iterations of re-running DNAcopy continue until less than 50 reads remain within the interval. **(c)** A second algorithm identifies the first read to map in a different direction (W read at chr13:19,203,283), then performs a check that the 10 preceding reads are all in the expected direction (10 C reads), and at least 20% of succeeding reads are in the other direction. The interval is refined to a distance between two reads. Abbreviations: C, Crick; W, Watson.

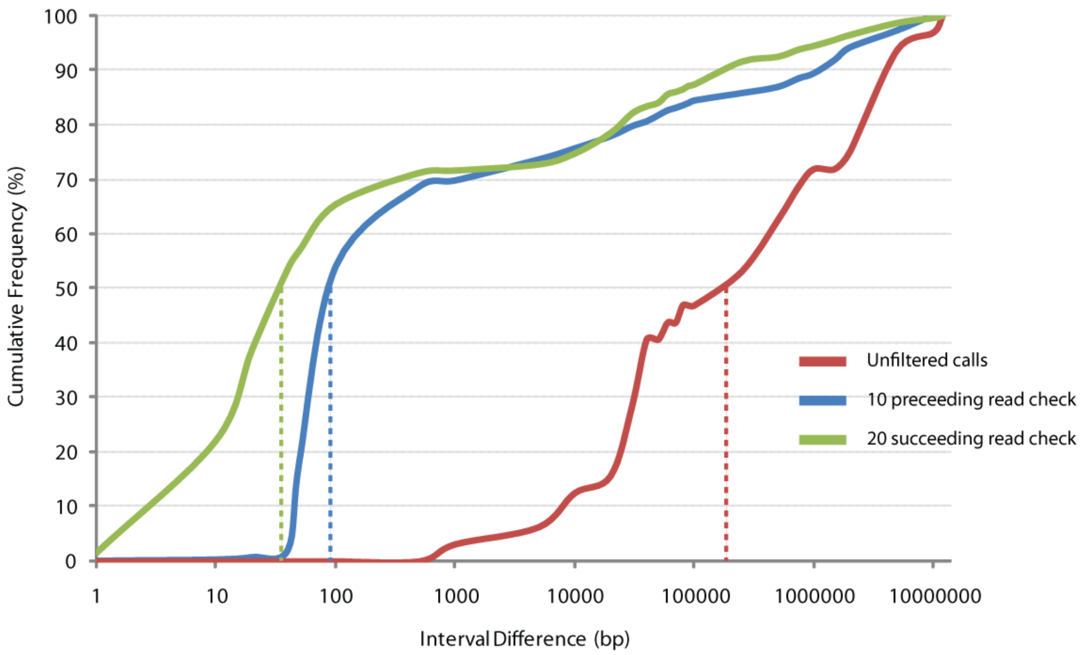

Figure 6.2: Optimization of the sister chromatid exchange (SCE) interval detector function. After DNAcopy has identified the smallest region in which SCE events have occurred, Bioinformatic Analysis of Inherited Templates (BAIT) executes a function that scans the region from the homozygous template direction until it identifies the first read mapping to the opposite strand. These "unfiltered calls" were similar to the manual calls, but were subject to low level background reads interfering with accurate localization (red line). To circumvent this, we added to the function that checks the 10 preceding reads to ensure they are all the same state, which yielded more accurate calls (blue line). Finally, we added a further check to ensure that the succeeding 20 reads (which are supposed to be Watson and Crick (WC)) mapped to the opposite strand at least 20% of the time (green line). The dashed lines indicate the median interval difference for each level of stringency.

centers, and there are very few techniques that are available for this task [125]. However, provided that the orphan scaffold has sufficient read coverage, strand-seq can be used to determine the strand-inheritance pattern, which will be the same as the chromosome on which it is present. For example, an orphan scaffold inheriting WC template strands must locate to a WC chromosome in that particular library. If an orphan scaffold inherits WW template strands, it will locate to a WW chromosome if both sequences are in the same orientation, or to a CC chromosome if it is misoriented with respect to the chromosome. On average, using just a single library, half of the chromosomes can be excluded as possible locations for these orphan scaffolds (Fig. 6.3).

By comparing these locations across a batch of libraries, BAIT localizes these scaffolds to particular chromosomes. For each orphan scaffold with sufficient reads, BAIT assigns a template state, compares this against the template state of each chromosome within a particular library, and then iterates this process to compute the concordance across all libraries. Concordance is never 100% in practice, owing to libraries with high background, orphan scaffolds with too few reads to accurately call strands, SCE events within gaps between the scaffolds, and the 5 to 10% error rate of BAIT in SCE detection. Nevertheless, BAIT is still able to achieve high-quality predictions of scaffold location by taking the highest-concordance chromosome. Chromosomes are further split based on SCE locations, allowing for localization of orphan scaffolds to particular chromosomal regions (Fig 6.3). Because orphan scaffolds are likely to be located within gap regions rather than within contiguous sequence, BAIT can use a provided BED-format gap file to cross-reference all mapped orphan scaffold locations to gaps within the same interval. BAIT outputs in a BED file both the best predicted region for each fragment and any candidate gaps within that region.

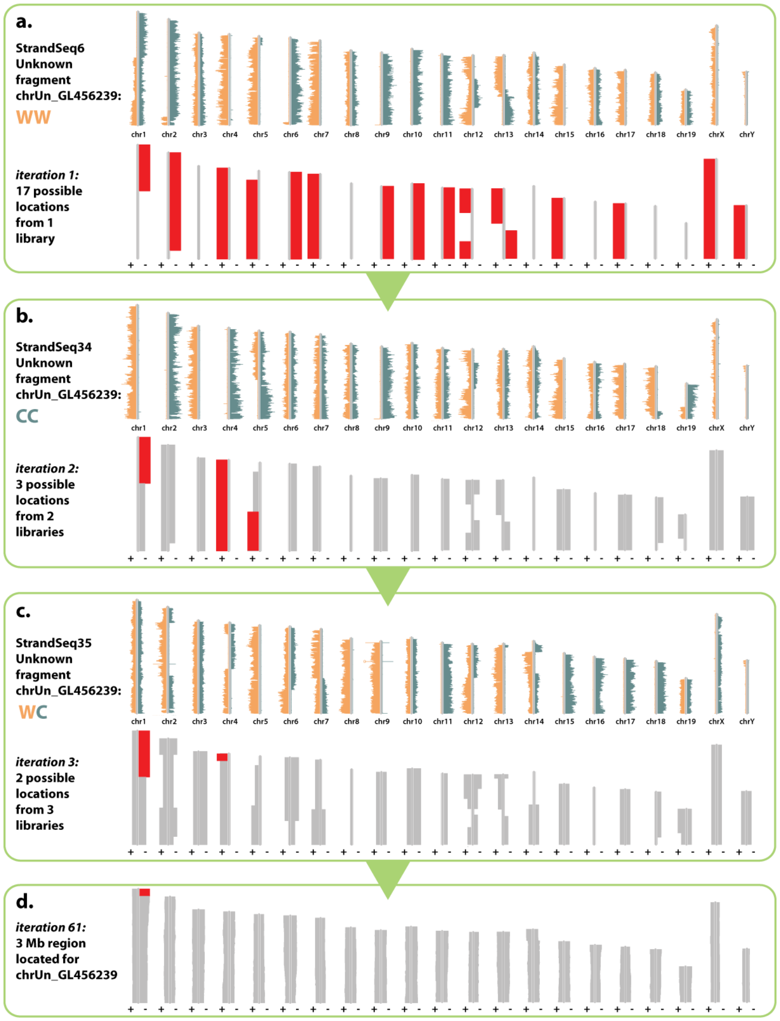

Figure 6.3: BAIT localizes unplaced scaffolds in late-version assemblies. Orphan scaffolds can be correctly oriented and localized relative to the rest of the genome by comparing template-strand inheritance. The orientation of an orphan scaffold is arbitrary, because it is not anchored to the rest of the genome, so it can be correctly oriented with respect its located chromosome, or misoriented. (a) For a single library where the unplaced scaffold GL456239.1 is WW, BAIT maps its potential location (shown in red) to both WW genomic regions (correctly oriented), and CC genomic regions (misoriented). If only one library is analyzed, all locations map with 100% concordance. Note that a WW scaffold will not locate to a WC chromosome, so chr8, chr14, chr16, chr18, and chr19 are 0% concordant. (b) BAIT iterates over a second library where GL456239.1 is CC. The results of the two libraries combined reduce the number of potential mapping locations from 17 to only 3 that map with 100% concordance. Because chr8, chr14, and chr16 are WC in this library also, these chromosomes map with 0% concordance. (c) BAIT iterates over a third library where GL456239.1 is WC, and thus maps to all chromosomes that are WC. The result of the three combined libraries reduces the number of potential mapping locations to 2: the centromeric tips of chr1 and chr4. (d) The combined results after iteration of all 62 libraries refine the location of GL456239.1 to the first 10 Mb of chr1 in the reverse orientation (with a concordance of 91%). The fragment was further refined to an unbridged gap occupying the first 3 Mb of chr1. Abbreviations: C, Crick; chr, chromosome; W, Watson.

6.3 Results

6.3.1 Accurate Localization and Mapping of SCEs

To assess the ability to computationally identify SCE events, BAIT predictions were compared with 528 SCE events from 62 murine embryonic stem cell strand-seq libraries that had previously been identified manually [83]. Manual processing of SCE events involved uploading BED-formatted strand-seq data into the UCSC genome browser [126], and identifying the interval at which the templates switch.

To allow the user to choose between favouring false positives or false negatives, BAIT SCE calling incorporates a filtering step, which excludes any bins that deviate more than a set multiple of standard deviations from the average red depth. By comparing the BAIT SCE calling to the manually processed SCEs, the optimal threshold for these data was determined. This was to exclude bins with read counts of ± 0.2 standard deviations from the mean, which gave a sensitivity of 0.93 (10.9% false positives), and a specificity of 0.89 (7.2% false negatives) (Fig 6.4a). When only those libraries with a low background metric ($< 5\%$) were included, the specificity improved to 0.94, while the sensitivity remained almost the same at 0.92 (Fig 6.4b). Of the false-negative calls, 72.9% were SCEs within 5 Mb of the start or end of the chromosome, indicating that terminal regions of chromosomes are under-represented by BAIT's SCE localization. In addition, three of the SCE events predicted by BAIT but absent in the manual analysis were determined to be correct upon further analysis. One event was less than 2 Mb from the distal telomere of chromosome 1, while the remaining two events were 5 Mb from each other on chromosome 13. These SCE events were difficult to detect by eye from a BAIT ideogram output of strand-seq data. Furthermore, because BAIT identifies SCE locations directly on ideograms with an arrowhead, both false-positive and false-negative SCEs can be rapidly scanned and validated from the ideogram output files.

Of the correctly identified SCE events, a comparison of the location of the SCE interval between automated and manual calls showed a median difference of just 34 bp (see Fig 6.2). Almost two-thirds (65.8%) of the predictions were within 100 bp

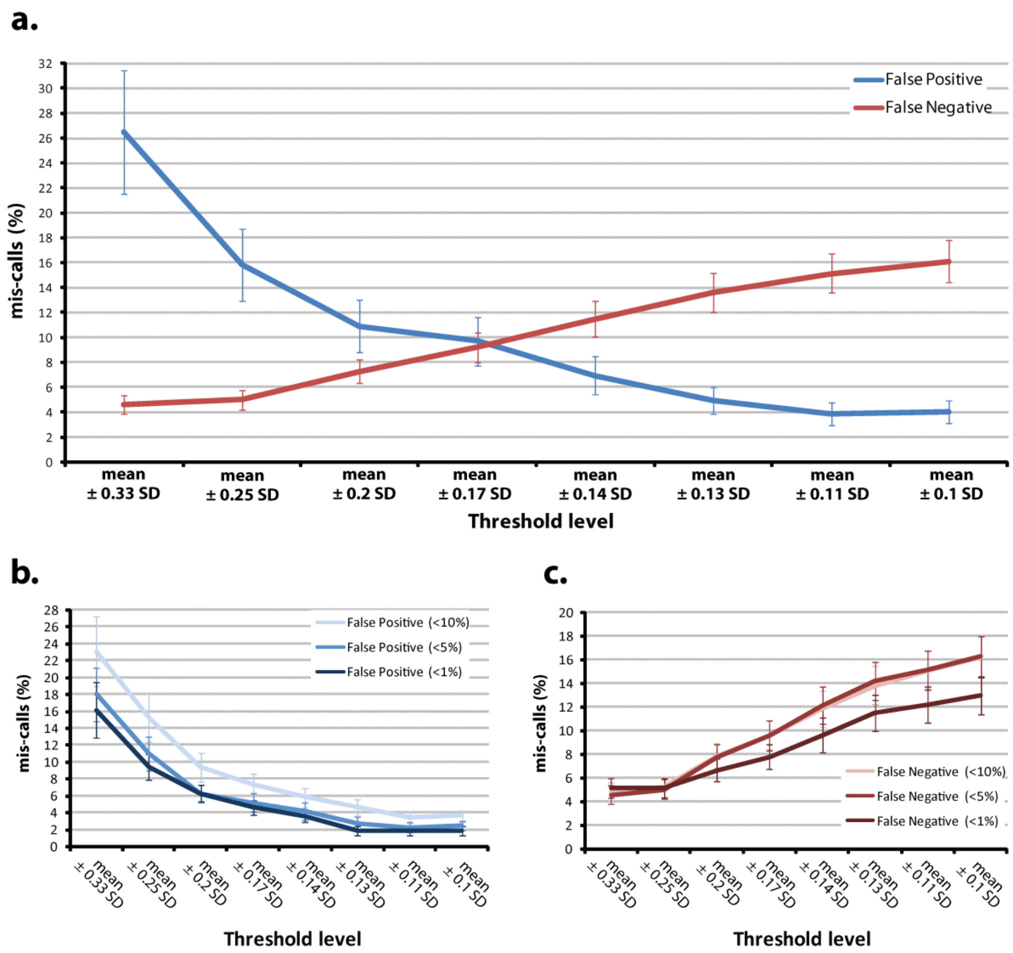

Figure 6.4: Accuracy of automated sister chromatid exchange (SCE) detection by Bioinformatic Analysis of Inherited Templates (BAIT). (a) By comparing the number of SCE events identified by BAIT to those determined manually, we calculated the percentage of computational calls that were incorrect (false positives) or not detected (false negatives). Filtering the data by only including bins that deviated minimally from the mean changed the results, with highly conservative filtering increasing the level of false negatives, and very broad filtering increasing the level of false positives. **(b)** The frequency of (left) false positives and (right) false negatives with respect to library background. Cleaner, high-quality libraries with $< 1\%$ of reads mapping incorrectly had a lower false-positive rate than libraries with medium background ($< 5\%$ incorrectly mapped reads), and an even lower rate than libraries with high background ($< 10\%$ incorrectly mapped reads). Error bars are $\pm$ standard deviation.

of the manual calls, with 74.7% of predictions within 10 kb. A summary of SCE distribution across all libraries was plotted, together with a histogram reporting the distance between events, helping to identify significant clustering of SCEs (see Additional file 2: Supplemental Data File 1). The accurate identification of SCEs is also important for the functions of BAIT which assemble and refine reference genomes (see sections below).

6.3.2 Placing Contigs in Late-build Genomes Using Strand Inheritance and Sister Chromatid Exchange Information

Previous work using strand-seq has shown that over 20 Mb of the MGSCv37/mm9 Mus musculus reference assembly is misoriented, involving 17 regions flanked by unbridged gaps [83]. In the more recent GRCm38/mm10 build of the genome, 35% (7,079.49 kb) of these identified misorientations were subsequently corrected, validating strand-seq with other approaches to correct orientation issues. In order to identify misorientations in the newest GRCm38/mm10 assembly, these analyses were repeated using the automated function of BAIT, identifying a total of 15 misoriented regions and five autosomal misorientations, with the remaining ten located to the X chromosome (see Additional File Table S1). Because the X chromosome only exists as one copy (monosomy) in the male embryonic stem cells (ESCs) of our dataset, misorientations appear indistinguishable from SCEs, and were identified by the intersection of events occurring over the same region across all libraries (see Additional file 2: Supplemental Data File 1). In this way, using just a single lane of sequencing, the majority of contigs (those larger than 10 kb with minimal segmental duplications) were oriented with respect to flanking contigs. Thus, using strand-seq and BAIT with relatively low-coverage sequencing, the relative orientation of all reference contigs can be determined, effectively bridging all gaps in an assembly.

Table 6.1: Identification of all misoriented fragments in GRCm38/mm10. The genomic regions that were incorrectly oriented in the latest assembly version of the mouse genome were calculated by Bioinformatic Analysis of Inherited Templates (BAIT). The location and lengths of these regions, which should all be present in the reverse complement in the reference assembly, are shown. Misorientations were identified in every informative library.

Fragment	Location	Size
JH584273.1	chr4:130,516,309-130,579,475	63167
JH584273.1	chr4:146,708,412-146,752,334	43922
GL456141.2	chr8:20,257,550-20,443,136	185586
GL456225.2	chr9:124,248,836-124,476,930	228094
GL456168.2	chr14:3,000,000-19,419,705	16419705
GL456186.2	chrX:3,182,394-3,556,356	373962
GL456186.2	chrX:4,742,210-4,960,182	217972
GL456190.1	chrX:27,205,755-27,499,671	293916
GL456192.2	chrX:27,549,670-29,212,945	1663275
GL456194.2	chrX:30,544,569-31,013,339	468770
GL456195.2	chrX:31,919,956-32,327,544	407588
GL456195.2	chrX:34,129,361-34,339,520	210159
GL456202.2	chrX:125,068,866-125,141,139	72273
JH584278.1	chrX:170,672,643-170,678,055	5412
GL456208.2	chrY:3,289,416-3,429,742	140326
**Total**		**20785352**

To validate the ability of BAIT to map scaffolds that have yet to be localized to regions on reference assemblies, we used it to predict the localization of all orphan scaffolds in an earlier assembly of the mouse reference (MGSCv37/mm9), and compared those predictions with the actual known locations in the current assembly (GRCm38/mm10). MGSCv37/mm9 has 60 useable orphan scaffolds that can be lifted to a single specific coordinate on GRCm38/mm10 [127]. Of these, 57 were located by BAIT to an interval coincident with the correct location on GRCm38/mm10 (Fig efamalgaBAITRes). From the three fragments that could not be correctly placed, two had fewer than ten libraries with sufficient read counts to analyze, and the remaining fragment mapped with a low concordance (57.1%). These data suggest reasonable thresholds for BAIT to map orphan scaffolds: more than ten libraries and greater than 60% concordance. More importantly, they confirm that using data from the same single lane of sequencing as used for contig orientation, BAIT and strand-seq can correctly map a large proportion of orphan scaffolds in a late assembly version.

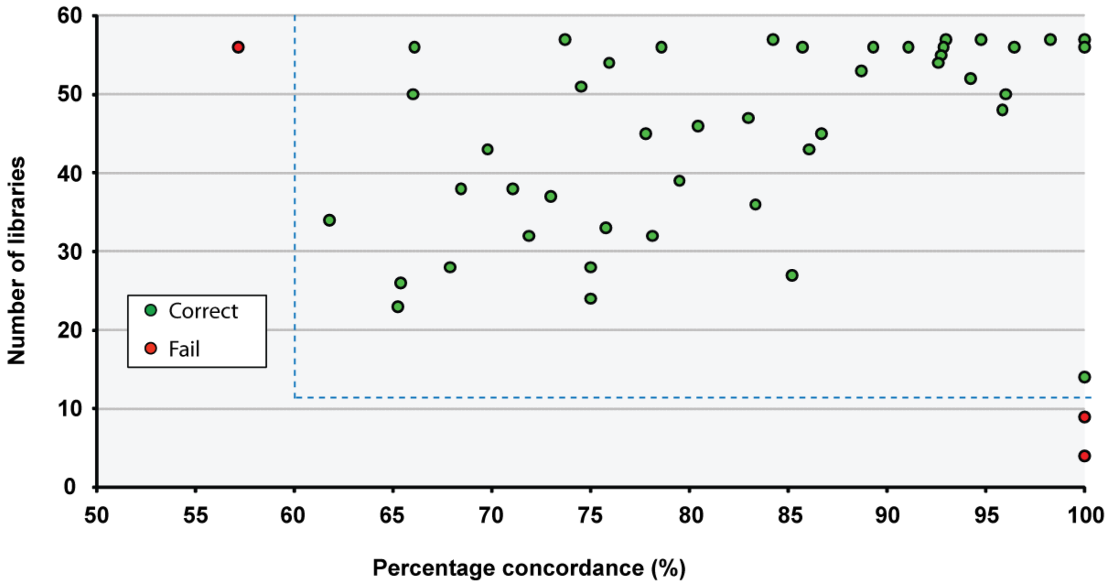

Figure 6.5: Validation of using strand-seq to map unplaced scaffolds to built genomes To confirm that Bioinformatic Analysis of Inherited Templates (BAIT) can successfully locate orphan scaffolds, the reads were aligned to MGSCv37/mm9, which has 202 orphan scaffolds, of which 60 can be mapped to a specific location in GRCm38/mm10. We used BAIT to locate these scaffolds in MGSCv37/mm9, and then cross-referenced these locations to the actual location in the GRCm38/mm10 assembly version. BAIT correctly located all regions in which there were more than ten libraries to analyze, and where the percentage concordance was above 68%. Green points indicate correctly mapped fragments, and red points indicate incorrectly mapped fragments. Dashed lines show the minimum number of libraries and minimal concordance needed to make confident calls.

There remain 44 orphan scaffolds in GRCm38/mm10, accounting for 5,334,105 bp, and containing 41 known genes. Of these, 23 contained sufficient reads to analyze, and we were able to subsequently place all of them to their matching chromosomes to within narrow intervals (Table 6.1; see Additional file 7: Supplemental Data File 2). By intersecting these locations to gaps in the contiguous genome build, BAIT further refined the scaffold locations (Table 6.1). Fragments were assumed to locate within either unbridged gaps or to bridged gaps in which gap size exceeded the fragment size, Analyzing 62 mouse libraries, 54.5% of these orphan scaffolds could be mapped to a particular chromosome, of which 54.2% could be mapped to a single contig gap (Table 6.1). BAIT also correctly oriented these fragments with respect to the chromosome to which they were mapped. BAIT includes a utility to create a new FASTA reference genome by reverse complementing misoriented regions and incorporating orphan scaffolds that map to a defined gap.

6.4 Discussion

In this chapter, I have described my contributions to BAIT, a software toolkit for automated analysis of strand-seq data. BAIT facilitates SCE analyses by rapidly counting and locating events, presenting a pipeline that can be incorporated into high-throughput strategies. BAIT accurately refines the interval between reads in which the template switch occurs, allowing regions with a high propensity to undergo SCE to be identified (for example, fragile sites [128] or sites of recurrent DNA damage). Accurate interval identification is also important in looking for genomic rearrangements such as translocations, and BAIT is able to detect these and assign a frequency of the rearrangement within the pool of libraries, requiring a far lower read depth than conventional split-pair read sequencing. [129] A caveat to these analyses is that SCEs and genomic rearrangements are more difficult to detect on chromosomes that have more than two copies within a cell, potentially limiting its use in highly polyploid cancer cells. Taken together, these results show that BAIT is very accurate and efficient at

predicting SCE intervals, and will be indispensable for future high-throughput analysis of strand-seq data.

BAIT is also capable of mapping orphan contigs in late-build genomes with a high level of accuracy. Furthermore, this accuracy can be increased to potentially 100% when disconcordant contigs (likely caused by either poor mappability or the presence of misorientations within the contig) and low-coverage contigs (short or those with poor mappability) are filtered out. For established and well-studied genomes, finishing builds by additional sequencing yields diminishing returns, and novel, targeted and highly sequence-efficient methodologies such as strand-seq and BAIT can play a crucial role in completing these genomes [130]. Indeed, I, along with Mark, have presented likely locations for 23 of the 44 remaining orphaned contigs in the current build of the mouse genome (GRCm38/mm10).

This work led on to Chapter 7, in which I describe methods for *de novo* assembly of early-build genomes made up entirely of unbridged contigs.

Chapter 7

ContiBAIT: Assembling Genomes Using strand-seq

7.1 Background

Early-build genome assemblies assembled by second-generation shotgun sequencing consist of many non-overlapping contigs, which are unanchored and unordered. However, performing strand-seq on cells derived from organisms with early assemblies yields directional strand information for each contig. Contigs residing on the same chromosome inherit the same templates across many libraries, while those from different chromosomes inherit template strands independently. Not accounting for sister chromatid exchanges, the probability that two contigs C_1 and C_2 from different chromosomes will have the same template state will be:

$$
\begin{aligned}
\text{P}(C_1 = C_2) &= \text{P}(C_1 = \text{WC}; C_2 = \text{WC}) + \text{P}(C_1 = \text{WW}; C_2 = \text{WW}) + \text{P}(C_1 = \text{CC}; C_2 = \text{CC}) &(7.1)\\
&= \text{P}(C_1 = \text{WC}).\text{P}(C_2 = \text{WC}) + \text{P}(C_1 = \text{WW}).\text{P}(C_2 = \text{WW}) + \text{P}(C_1 = \text{CC}).\text{P}(C_2 = \text{CC}) &(7.2)\\
&= 0.5 \times 0.5 + 0.25 \times 0.25 + 0.25 \times 0.25 &(7.3)\\
&= 0.375 &(7.4)
\end{aligned}
$$

Conversely, contigs from the same chromosome will inherit the same template strands across all libraries (although this will be confounded slightly by sister chromatid exchanges). This similarity to genetic linkage [131] makes concordance of strand inheritance a good metric for clustering contigs into putative chromosomes or "linkage groups".

Some early-build genome assemblies have anchored contigs to chromosomes, but have not placed those contigs in order within each chromosome, due to the lack of overlapping information for assembly. Furthermore, after anchoring contigs to chromosomes using strand-seq, it would be desirable to also order them. In this case, sister chromatid exchanges can be used. For anchoring contigs, SCEs are an undesirable source of noise, causing deviation from the ideal case in which every contig from the same chromosome has the same strand template inheritance pattern in every library. But in every library in which an SCE has occurred, this provides ordering information for the chromosome it occurred in. Contigs upstream of the SCE will have the same strand inheritance as each other in that library, and the same for those downstream. Similarly to how meiotic recombination is used to create a genetic linkage map between loci [132], SCE events along the chromosome can be used to determine a genetic distance between contigs on the same chromosome, allowing them to be arranged and ordered. This is illustrated in Fig 7.4a.

In this chapter, I present contiBAIT, an R package for using strand-seq to both anchor and order unbridged contigs in early-build genomes.

7.2 Methodology

7.2.1 Preprocessing and Calling Template Strand State

Before attempting anchoring and ordering, ContiBAIT performs filtering of libraries and contigs. ContiBAIT excludes libraries where every contig has inherited WC templates, as these suggest a failure of the strand-seq protocol. Contigs that have inherited WC templates in all libraries are also excluded, as these likely contain degenerate sequence.

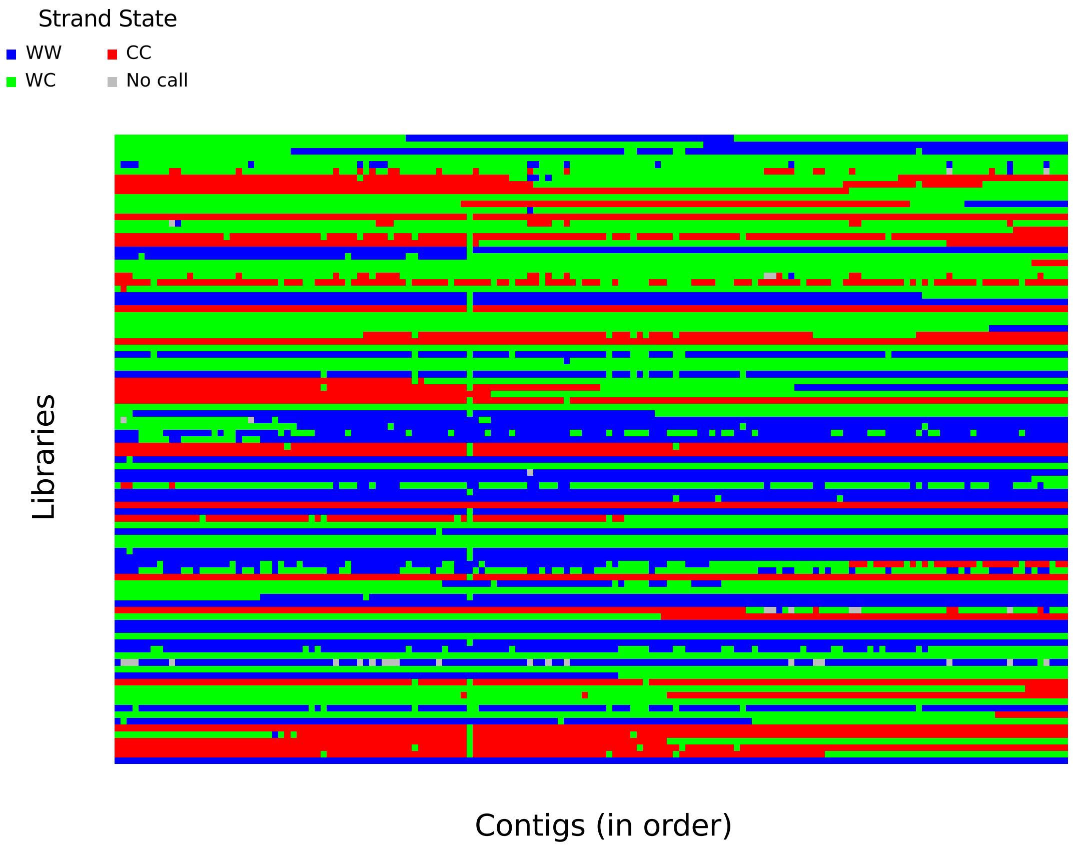

Figure 7.1: True ordering of semisynthetic mouse data for chromosome 3. This plot was generated using 1 Mb artificial contigs created based on the mm10 genomic assembly. The contigs for a single linkage group, consisting mainly of chromosome 3, were used. This illustrates some of the difficulties with the data – while some libraries are called cleanly, and show a consistent state either throughout or until there is an SCE, others are extremely noisy, with apparent state changes every few contigs.

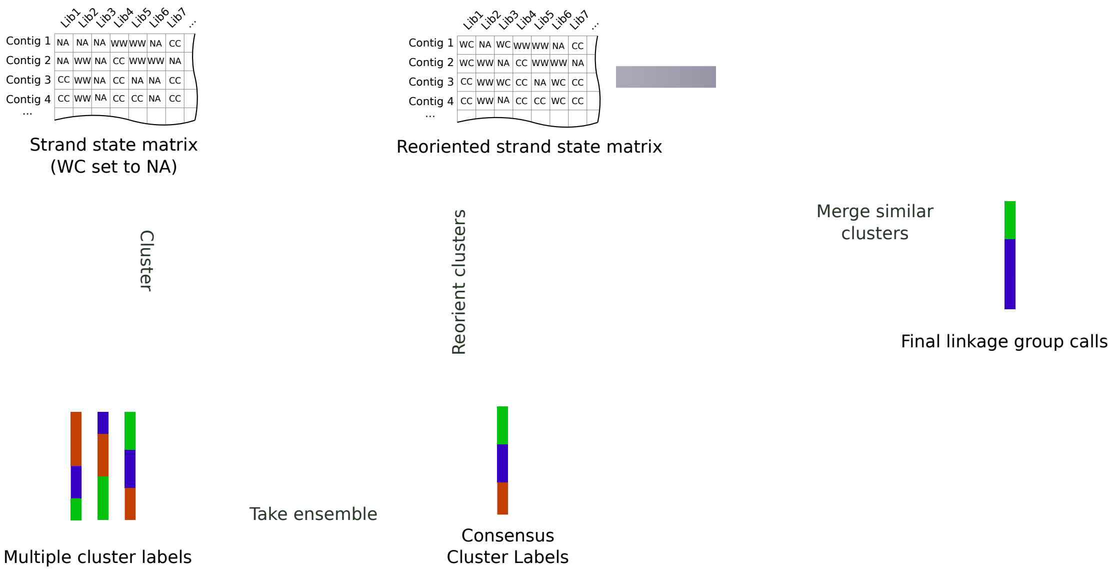

Figure 7.2: Overall ContiBAIT Linkage Group Assignment Pipeline. A matrix of strand states for each contig in each library (cell sequenced) is taken as input. For clustering, only the WW and CC states are used; WC are set to NA. Several clusterings runs are performed, and combined to form a consensus clustering. The clusters most dissimilar to other clusters are reoriented until this does not improve the global cluster score; the strand matrix is then updated with the reoriented strand states. Clusters that are similar by the reoriented strand matrix, including the WC calls, are merged, producing the final call for linkage group membership.

7.2.2 Anchoring Unbridged Contigs into Chromosomes *de novo* in Early-build Genomes

Using the template strand calls matrix from the previous step, contiBAIT assembles contigs into groups with similar strand inheritance patterns (linkage groups), representing putative chromosomes. The complete contiBAIT pipeline for contig anchoring is overviewed in Fig 7.2. In the first step, only CC and WW states are used to cluster the contigs; this is to keep contigs from the same chromosome but which are in reverse orientation separate. Due to the non-deterministic nature of the clustering algorithm used, an ensemble of multiple clusterings is used to determine the best cluster labels. Whole clusters are then reoriented until the global cluster distance no longer improves, and the strand matrix is updated with the reoriented strand template states. This complete matrix is used to determine distances between clusters, and similar clusters are merged into the final linkage groups.

Three-Star Chinese Restaurant Clustering

A major challenge which arose in [6] was the great number of contigs which had to be clustered into putative chromosomes. In the mm2 data set, the number of contigs which had sufficient strand-seq data to cluster was approximately 50,000. This made distance-based clustering algorithms infeasible, as this would require a distance matrix containing 2.5×10^9, stretching the limits of all but highly specialised hardware to store in RAM.

A method does exist for memory-efficient clustering of categorical data – k-modes [133]. However, this algorithm in its original form does not provide for a method of model selection (choosing the number of clusters k). Furthermore, k-modes is not designed to handle missing data, which is a necessary feature of strand state calls. I thus concluded that developing a novel clustering algorithm would be the best solution to this problem. To this end, I created an algorithm very loosely modelled on a Chinese restaurant process.

A Chinese restaurant process (CRP) posits a restaurant with infinitely many tables,

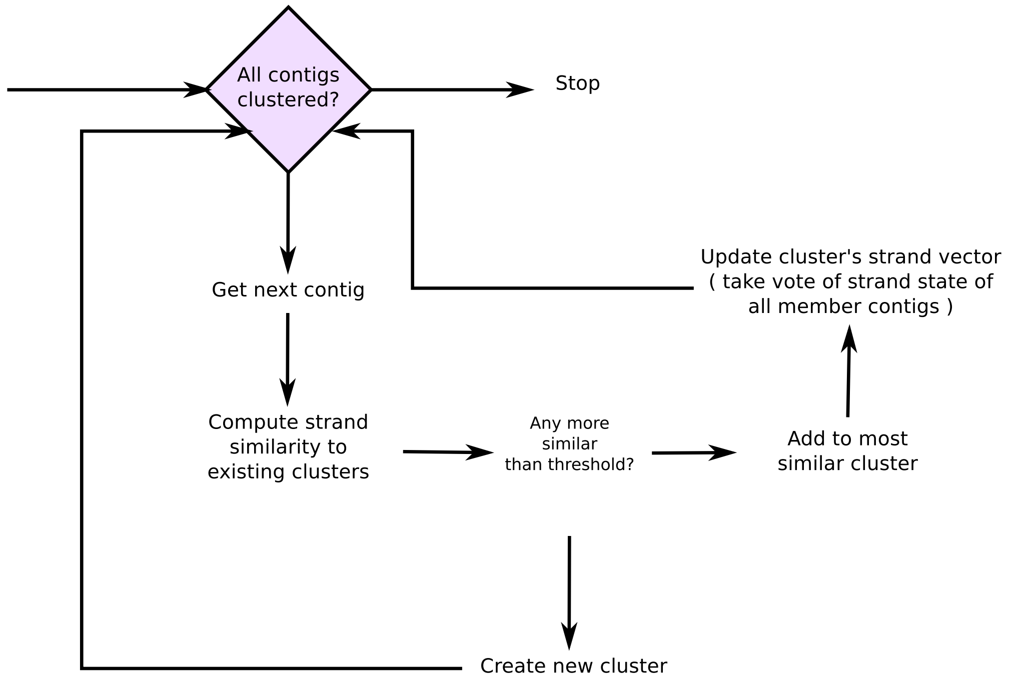

Figure 7.3: Flow diagram illustrating three-star Chinese restaurant clustering. For each contig, the similarity in strand state between the contig and the existing clusters (if any) is computed. If this similarity exceeds a pre-defined threshold for any of the existing clusters, the contig is assigned to the most similar cluster. If no clusters are similar enough, a new cluster is created. For the purposes of computing similarity, a consensus strand state is computed for each cluster by a simple vote of the strand states of its members.

each having infinitely many chairs. As each new customer is seated, they choose, with uniform probability, to sit at an already-occupied table, or to sit at a new table. Indeed, a model-based clustering method for numerical gene expression data has been developed from CRPs [134].

My clustering method was considerably simpler. In each iteration, a new contig is considered. The contig may either be assigned to a new cluster, or join an existing cluster. To decide this, each existing cluster's per-library strand state is computed as the majority vote of its member contigs for that library. Then, the distance between the new contig and all the existing cluster is computed. If the contig is sufficiently similar to an existing cluster (as determined by a pre-set threshold similarity value), then it is added to that cluster. If not, then it forms a new cluster.

In a deliberate effort to stretch a bad metaphor to the breaking point, I have named this method “Three-Star Chinese Restaurant Clustering”. In Vancouver, the city where this work was conducted, there are many Chinese restaurants, serving a great range of Chinese cuisines. As a one to five star rating system is common for restaurant reviews, I have selected “three-star” to refer to an “average” restaurant, as an allusion to the averaging of existing cluster values when choosing cluster assignment for a new data point.

High-Speed Bitstring Implementation of Contig Similarity Calculation

In order to enhance the performance of TSCRC for this particular task, I optimised the distance computation for speed. Since the strand state vector for each contig is defined over only two values – WW and CC, it could be encoded as a bit string ($V1_S$ and $V2_S$), using the BitString class from the boost library [135]. The presence of missing values (NAs) in each vector is encoded as a separate bitstring ($V1_{NA}$ and $V2_{NA}$). Then, using bitstring arithmetic, the similarity between two strand state vectors, in the presence of missing values, can be computed:

$$\text{common bits } C = \sim (V1_{NA} | V2_{NA}) \tag{7.5}$$

$$\text{equal bits } E = \sim (V1_S \oplus V2_S) \tag{7.6}$$

$$\text{non-NA overlap } O = E \& C \tag{7.7}$$

$$\text{similarity } S(\in \mathbb{R}) = \text{bitcount}(O)/\text{bitcount}(C) \tag{7.8}$$

Bitwise AND, OR, XOR and NOT are part of the core Intel x86 instruction set. Counting of set bits in a bitstring is implemented as the machine-level instruction POPCNT since the SSE4 extension to the basic Intel instruction set [136]. As such, this implementation can be translated by the C++ compiler down to high-speed machine-level instructions on most modern computers.

Ordering Clustering by Quality

One problem with TSCRC is its sensitivity to the order in which contigs are presented to it. Lower-information contigs are more likely to associate incorrectly, and if these form the seeds of clusters at an early stage in the clustering, they could result in clusters made up erroneously of contigs from separate chromosomes. Indeed, this was the case in the initial validation work shown in Fig 7.5. A solution to this problem is to feed in contigs in order of quality. For this purpose, I used the proportion of non-NA values for each contig, and sorted the contigs in descending order.

Ensemble Clustering

To further overcome TSCRC's sensitivity to ordering of its input, contiBAIT performs several clustering runs and combines these using ensemble clustering. In order to keep the advantages of best-first ordering while still randomising the clusterings to be combined in the ensemble, contiBAIT uses quality-weighted sampling of contigs to select the order in which they are passed to TSCRC for each clustering run. A consensus clustering is then produced using the CLUE R package with the default fixed-point algorithm [31].

Reorientation and Merging of Linkage Groups

To distinguish contig orientation, contiBAIT generates an initial contig dissimilarity matrix using only chromosomes that have inherited homozygous WW and CC templates (but excluding WC), in such a way that misoriented linkage groups derived from the same chromosome are highly dissimilar (Fig 7.5a, left panel). BAIT then uses a greedy algorithm to reorient the misoriented linkage groups: iteratively inverting the most dissimilar, and recomputing the distance matrix until a reorientation causes no increase in the summed concordance of all groups (Fig 7.5a, right panel; see Fig 7.6). Linkage groups with high similarity are merged in the recomputed data, and contiBAIT has functionality to visualize this as a distance-matrix heat plot of linkage group concordance (Fig 7.5a, right panel; see Fig 7.6).

The final output of contiBAIT's anchoring process is a linkage group label for each contig, corresponding to its chromosome membership. As a side effect of the reorientation process, contiBAIT also produces orientation information for all contigs.

7.2.3 Ordering of Unbridged Contigs within Chromosomes

To determine the order of contigs within chromosomes, contiBAIT uses template-strand inheritance and SCE localization to build an inter-contig distance matrix for each linkage group. This is then used for ordering by formulating the ordering problem as a travelling salesman problem.

If this distance matrix is viewed as a complete graph, then the goal is to find a path that visits every vertex (a Hamiltonian path), while minimising the weight of the edges included (the lowest-weight Hamiltonian path). However, given the far greater availability of techniques for finding the lowest-weight Hamiltonian Cycle (the travelling salesman problem), it is desirable to reformulate the problem as such. This can be achieved by adding a dummy vertex with edges of weight zero to all other vertices. From there, existing methods for solving the travelling salesman problem may be used to find a (hopefully optimal) low-weight path through the graph and infer the relative order of contigs within a linkage group. This process is illustrated on a toy example in Fig 7.4.

Before computing this distance matrix, additional pre-filtering is carried out. To reduce noise, contiBAIT re-examines the ratios R of W to C reads used to call the strand state for each contig in each library. These calls are filtered out (set to NA) when $0.2 < abs(R) < 0.8$. Since no ordering information can be found for contigs which are so close that their strand state is identical across all libraries (see Figure 7.4), these cases are detected and combined into meta-contigs before ordering.

Following filtering, contiBAIT computes distances between contigs using the daisy function from the cluster package in R [137]. It then passes these distances to the TSP package to be solved. Unfortunately Concorde [138], widely considered to be the best TSP solver, cannot be included with the TSP package due to licensing issues. So, at

present, only the 2-opt method [139] is provided.

7.3 Results and Validation

7.3.1 Contig Anchoring

To test the ability of BAIT to build genomes *de novo*, the read libraries were realigned to the first build of the mouse genome (MGSCv3). Of the 224,713 contigs in this assembly version, the 77,258 that were over 10 kb were included, representing 2,006 Mb of DNA (81.0% of total assembly). After remerging and reorienting similar clusters, BAIT assigned 54,832 contigs, representing 1,742 Mb (64.9%) of the assembly, into 20 primary LGs (Fig 7.5a). Allosomes in these male-derived ESCs are effectively monosome, and so contigs derived from the sex chromosomes can be separately identified, as they only inherit a single W or C template strand, never both. After cross-referencing the locations of MGSCv3 contigs to GRCm38/mm10 coordinates, the majority of LGs clustered to only one chromosome (see Fig 7.6), and the majority of chromosomes consisted of only one linkage group (Fig 7.5b). When more than one chromosome was attributed to the same linkage group, these groups could be split into two subclusters (see Figure 7.6).

Similar results were seen when we simulated an early-stage reference by splitting the GRCm38/mm10 genome into a scaffold of the 403 chromosomal Giemsa bands (based on coordinates from the UCSC genome browser [127]), and realigned our libraries to this new reference version (see Additional file 5: Figure S4). Using disrupted concordance from SCEs as a genetic distance indicator, it was further possible to infer the relative orders of the contigs present in each linkage group.

The accuracy of ordering fragments is dependent on the frequency of SCEs, the number of libraries used in the analysis, and the level of library background (high-background libraries are more likely to have incorrect template calls). If the template strands of contigs are identical in all libraries (because no SCE events have occurred between them) their relative order remains unknown.

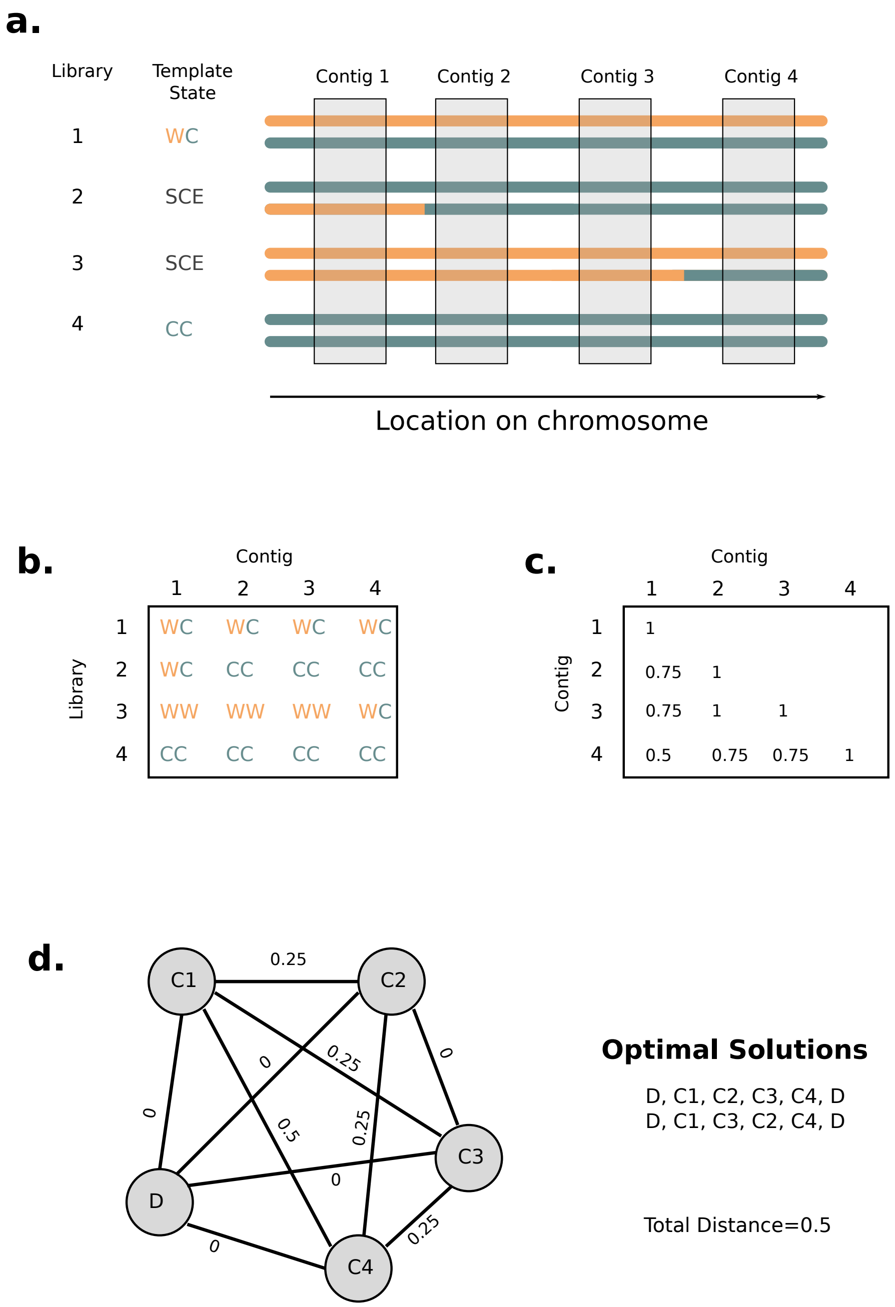

Figure 7.4: Using SCEs to order contigs within a chromosome. **a.** A very simplified and idealised example, showing only four contigs and four libraries. **b.** The template state matrix for each contig in each library. **c.** The concordance matrix between contigs, corresponding to the template matrix in b. **d.** The concordance matrix formulated as a distance graph, with an added dummy node D, for solving as a travelling salesman problem. Two shortest Hamiltonian Cycles are possible, and once the dummy node is removed, one of these corresponds to the correct ordering of the contigs along the chromosome. Note that, since C2 and C3 have identical template state in all libraries, it is impossible to distinguish their ordering.

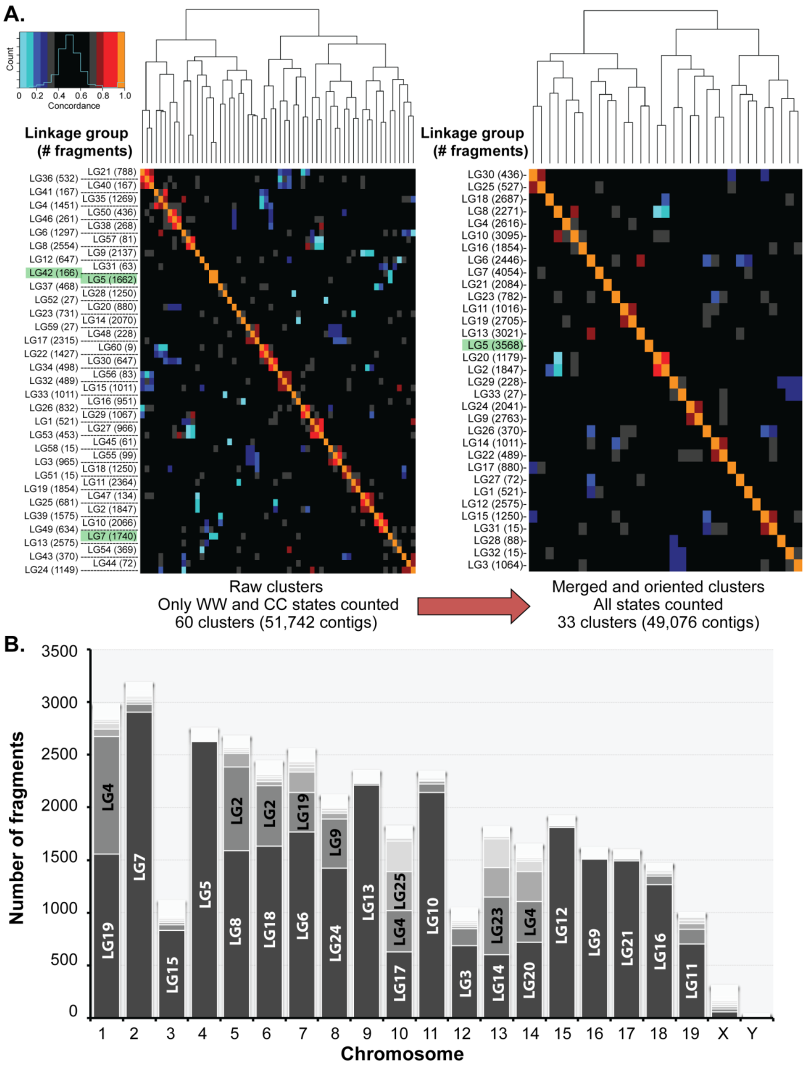

Figure 7.5: Clustering contigs into linkage groups for early-assembly genomes. Using template strand directionality as a unique signature, all contigs in the early mouse assembly MGSCv3 were compared with each other across all 62 strand-seq libraries. All contigs with similar (> 85%) template inheritance patterns were stratified into linkage groups (LGs). **(a)** Heat plots of all BAIT-called LGs show limited similarity between groups. Through analysis of homozygous template states only (WW and CC, left panel) 57,581 contigs cluster into 33 LGs, with the association between linkage groups appearing as yellow points if groups are in the same orientation, or blue points if the groups are in opposite orientations. The LGs are then reanalyzed after merging and reorientation of associated clusters, resulting in only 20 linkage groups consisting of 54,832 contigs. **(b)** Histogram of the number of fragments within a linkage group that map to a particular chromosome. The LG with the largest number of contigs are shown at the bottom in dark gray, with groups that contain the next largest numbers of contigs shown in progressively lighter grays. Most LGs contain contigs that belong to the same chromosome (see Fig 7.6), and in general, most chromosomes are represented by one or two linkage groups. Note: contigs derived from sex chromosomes in male libraries can be distinguished as they are haploid, and are not computed as an initial heat plot. Any contigs derived from haploid chromosomes are separated and clustered independently. Almost all contigs clustered into this linkage group mapped to the X chromosome (right histogram). Abbreviations: C, Crick; W, Watson.

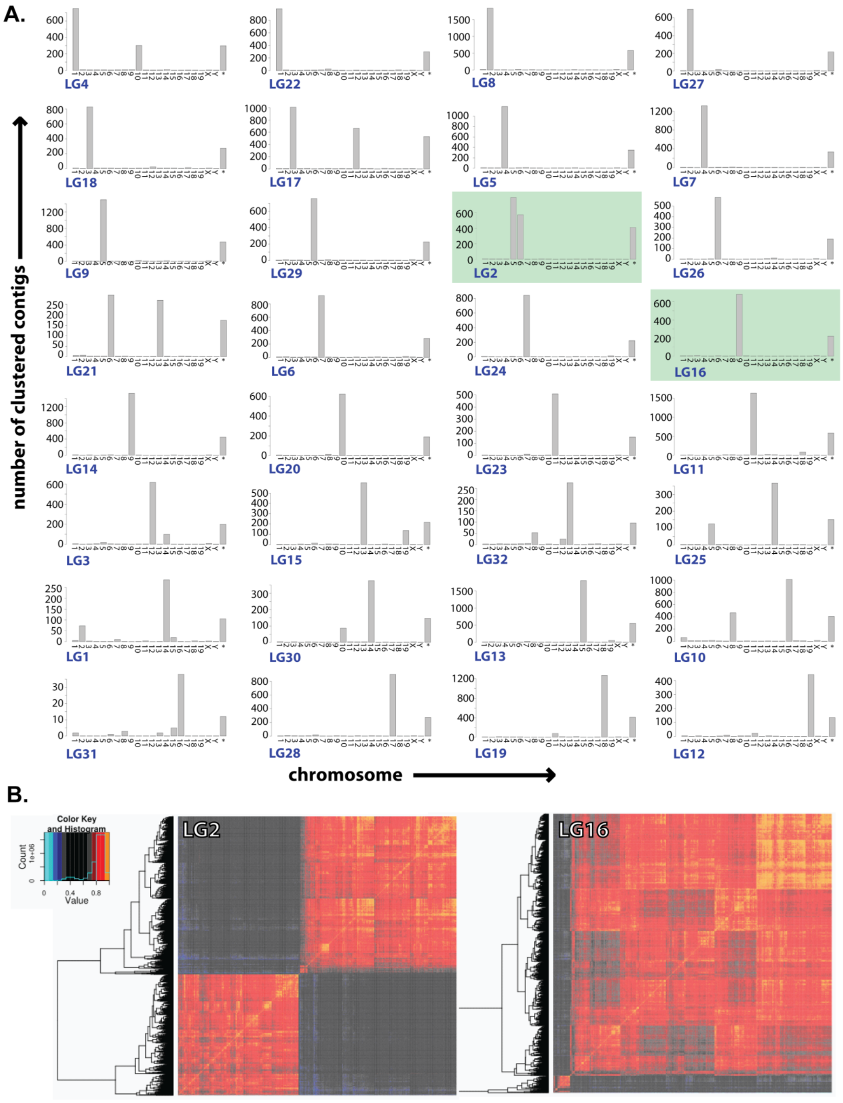

Figure 7.6: Contig locations in Bioinformatic Analysis of Inherited Templates (BAIT)-compiled linkage groups. strand-seq data was aligned to the MGSCv3 assembly, and was stratified solely on strand inheritance patterns. The resulting data were compared directly to the known locations of the MGSCv3 contigs in the current GRCm38/mm10 assembly. **(a)** Linkage groups determined by BAIT predominantly contain contigs derived from a single chromosome. The linkage groups (denoted as LG# under each histogram) contain different numbers of clustered contigs, with the total length of each linkage group shown (y-axis) but tend to map to only one chromosome. **(b)** Of the linkage groups that map to more than one chromosome, a heatmap plot shows that the linkage group should be subdivided. Linkage group 1 (green highlight) contains contigs from chromosome 1 (chr1), chr15, and chr7, but generates three distinct clusters (left panel) where each cluster contains contigs derived from one chromosome (colours beneath dendrogram). An example of a linkage group with contigs mapping to a single locus (LG11, green highlight) shows that the majority of contigs within this group cluster tightly together (right panel).

Ensemble Clustering

To evaluate ensemble clustering, I ran TSCRC 100 times on the early mouse genome (mm2) data used for evaluation in [6]. I ran reorientation and merging on each of these, and took those results as the non-ensemble baseline. I then took $1,000$ bootstrap samples for each of size $\{2, 4, 6, 8, 10, 12, 14, 16\}$, and reoriented and merged each of these. To evaluate the accuracy of each clustering, I computed the F-measure as per [5], specifically:

$$F(C, K) = \sum_{c_i \in C} \frac{c_i}{N} \max_{k_j \in K} \{F(c_i, k_j)\} \tag{7.9}$$

Where C represents the true chromosome assignments of contigs (lifted over to mm10), and K represents the cluster assignments produced by contiBAIT.

The results are shown in Figure 7.7a. Ensembles of size 4–16 showed improved F-measures over single clusterings, with a peak at 14, and most of the gains achieved by 8 clusters in the ensemble.

Effect of Contig Size

Using the current build of the mouse genome (mm10), I divided chromosomes into equal-sized sections of varying sizes (1 Mb, 500 Kb, 250 Kb, 100 Kb, 50 Kb). I then ran contiBAIT 100 times (without ensemble) on each of these to assemble them into their original chromosomes, and computed the F-measure for each as per Equation 7.9.

The results are shown in Figure 7.7b. Contig size, at least over this range, had minimal effect on F-measure. Although there was an increase in F-measure with decreasing contig size down to 100kb, on examination of the clusterings themselves, this was due to there being only 1-2 contigs misplaced in each result. Those 1-2 misplaced contigs represented a greater proportion of the total chromosome size in the data with larger contigs, hence the slightly lowered F-measure.

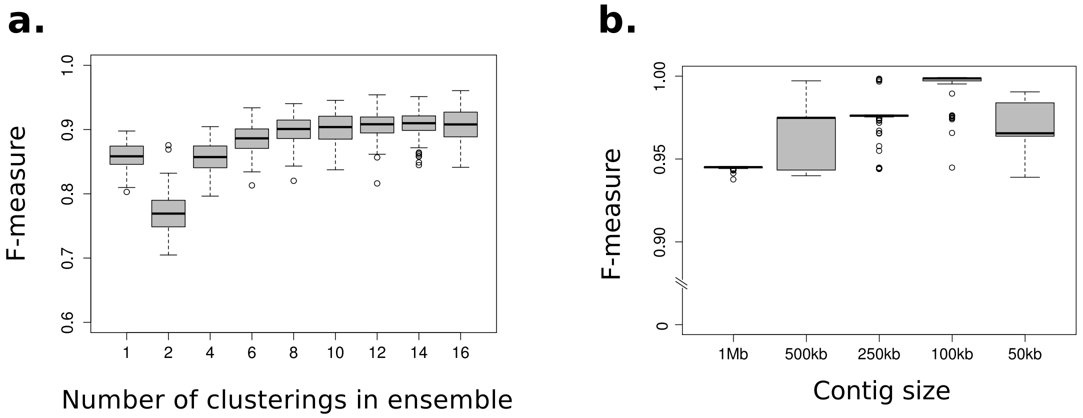

Figure 7.7: Evaluation of contiBAIT chromosome assignment with different-sized cluster ensembles and different-sized contigs. a. F-measure vs size of cluster ensemble for mm2 data. Without ensemble clustering, median F-measure was 0.86. Ensembles of size 2 actually decreased F-measure (median=0.77), while those of size 4 and larger show an overall (but not significant) increase, peaking at ensembles of size 12–14 (median=0.91). b. Effect of contig size on accuracy of chromosome assignment for uniform-sized synthetic contigs from mm10 data. F-measure was relatively uniform (within the range of 0.94 to 1), however there was a slight trend of increasing F-measure as contig size decreased from 1 Mb down to 100 Kb. This trend reversed at 50kb, with a slight decrease in F-measure from 100 Kb.

Overall Performance

ContiBAIT was run on the same early-build genome data (mm2) described earlier in Fig 7.5 and in [6], but with TSCRC and ensemble clustering. The results are shown in Fig 7.8, and include no major incorrect merging of contigs from different chromosomes, and most chromosomes almost entirely accounted for by a single linkage group.

7.3.2 Validation of Contig Ordering

To validate contig ordering, I used the 1 Mb, 500 Kb, 250 Kb simulated contigs as described in the earlier section for anchoring. I took the contigs assigned to each linkage group as predicted by contiBAIT anchoring, and applied contiBAIT to attempt to correctly order them. I then compared the positions of the contigs in order to their true start positions, both visually and by using Spearman's Rho correlation. These results are summarised in Fig 7.9. Visually, contiBAIT was able to recapture the overall trend of contig location, although frequently there were discontinuities and opposite-ordered

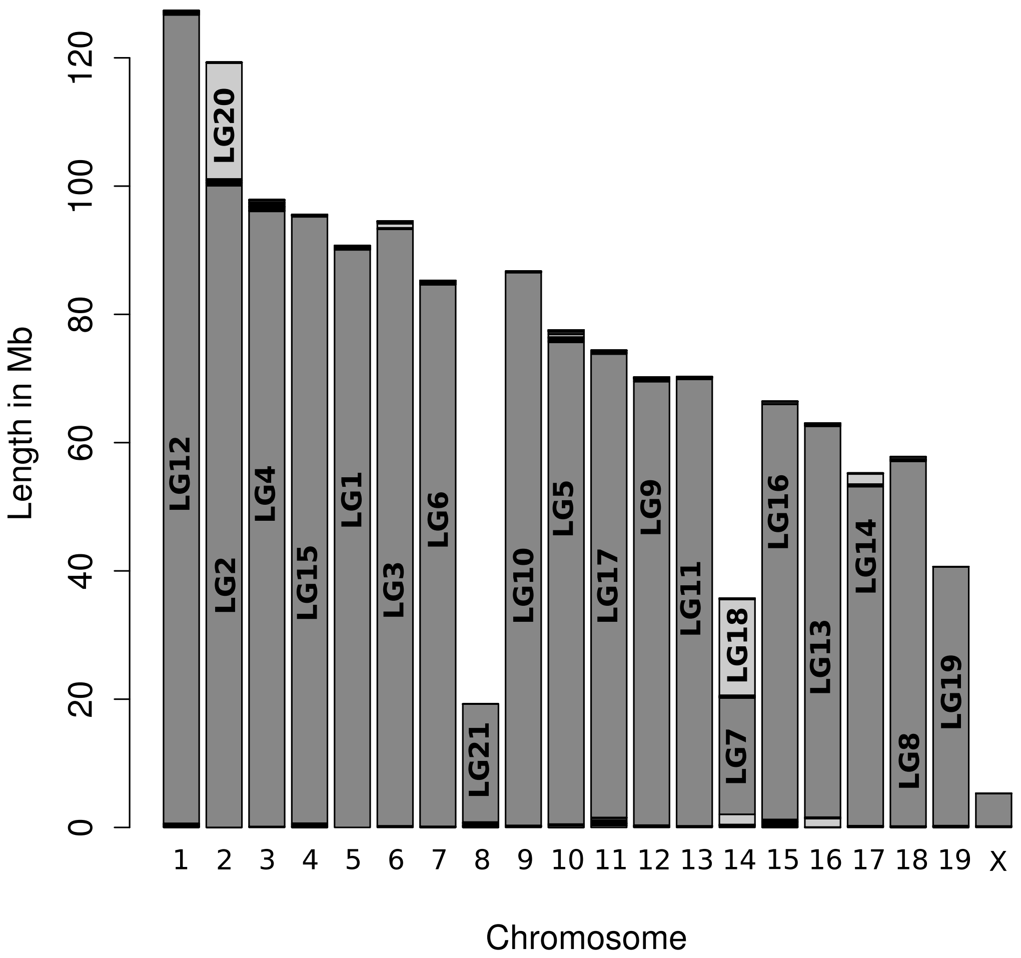

Figure 7.8: A chromosome assignment result after reorientation and merging. This plot was generated on mm2 data, following clustering with an ensemble of size 8, reorienting of misoriented linkage groups, and merging. This is a marked improvement in comparison with the previously published performance shown in Figure 7.5B. No linkage group contains substantial numbers of contigs from any but the primary chromosome, so that, although chromosomes 2 and 14 ended up split over two main linkage groups each, no chimeric chromosomes were created.

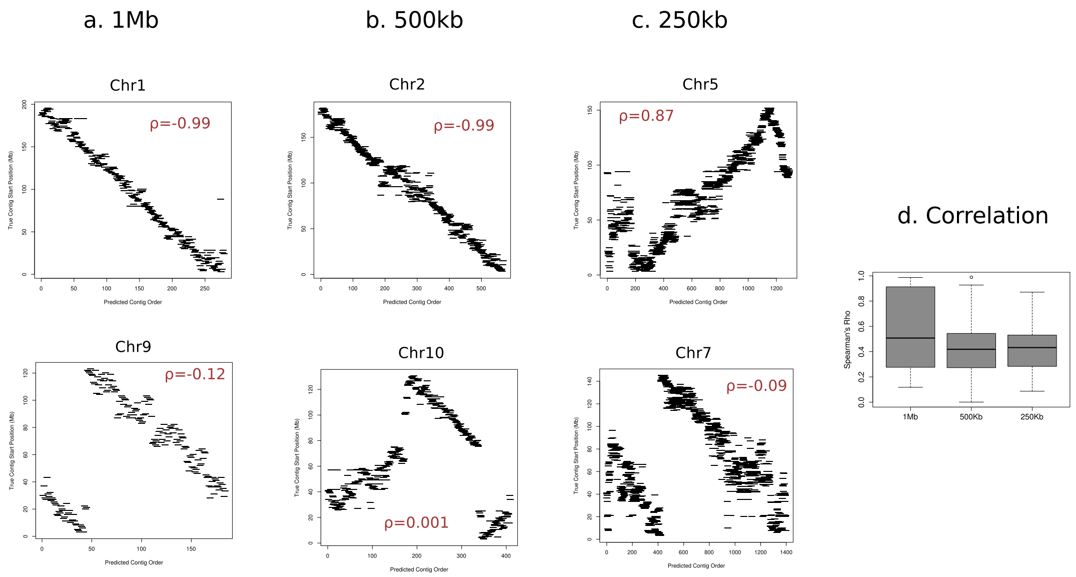

Figure 7.9: Testing of ordering of contigs on synthetic data Predicted ordering is plotted against true start positions of contigs. **a–c.** For each of the data sets tested, the linkage groups with the best and worst Spearman correlation are shown. **d.** Distribution of Spearman correlation for each of the data sets. Both the maximum and the mean decrease with smaller contig sizes.

regions.

7.4 Package

Unlike BAIT, ContiBAIT has been developed as a fully-contained R package, capable of accepting BAM and BED files and performing all analysis described in this chapter. It is intended for it to submitted to Bioconductor to be made freely available.

7.5 Discussion

I have demonstrated that contiBAIT can anchor contigs into common linkage groups based on shared template inheritance, and can order some contigs within linkage groups using sister chromatid exchanges.

For contig anchoring, I have presented a complete pipeline for clustering and reorienting contigs. As part of that pipeline, I have developed and optimised three-star Chinese

restaurant clustering, a novel, memory-efficient clustering algorithm for symmetric binary variables. Although this method is somewhat susceptible to errors due to starting bias, I have shown that combining multiple runs in an ensemble of six or more clusterings improves its performance. On an actual early-assembly genome, assigning of contigs to chromosomes performed well (mean F-measure 0.91). On simulated genomes, created by cutting the current (mm10) build of the *Mus musculis* genome into uniformly sized "contigs", F-measure consistently exceeded 0.95 even for contigs as small as 50kb.

For contig ordering, I have presented a pipeline for assigning the orders of contigs relative to each other using a travelling salesman problem formulation. This was able to capture the overall ordering of contigs, but with a great deal of local noise, as well as regions ordered opposite to each other. While this may be sufficient for the purposes of early-build genomes (and certainly provides more information than is available for unbridged contigs), this is far from ideal. It is possible that the use of the Concorde TSP solver [138] may improve the ordering. Furthermore, more stringent filtering of both libraries and contigs could reduce the confounding noise, at the expense of fewer contigs ordered. It would also be possible to first order a small subset of higher-quality contigs, then use BAIT to detect misorientations and to place some of the remaining contigs [6].

Preliminary sequencing efforts in lesser-studied organisms suffer from fewer resources spent on deep sequencing and subsequent curating and refining of the reference genome assemblies. With several ambitious sequencing projects in development [140], there is an increasing need for rapid and cost-effective construction of accurate and useful reference genomes. Arranging contigs to facilitate building chromosome-level and genome-level hierarchies represents an attractive advance toward this goal, especially in conjunction with existing technologies, and it is likely that strand-seq and contiBAIT will be widely adopted in standard genome assembly pipelines [130]. ContiBAIT is positioned to join alternate, low-cost methods for anchoring contigs in draft-quality genomes, such as optical mapping,[141] chromatin interactions [142] and POPSEQ [143] as a vital tool for low-budget genome sequencing efforts.

Chapter 8

Conclusions and Future Work

8.1 Summary of Contributions

Firstly, I have developed an enhanced version of flowType, enabling it to be used on newer, higher dimensional data. I have developed flowBin, a new, imputation-free tool for combining multitube flow cytometry data. Both of these steps were necessary to enable deep, automated profiling of retrospective flow cytometry data from AML diagnoses. FlowBin was needed to transform the data into a form that could be analysed while accounting for the true correlations among the markers in different tubes. FlowType-DP was necessary to analyse the very high-dimensional data from flowBin, which was not possible with flowType-BF. I have shown how, taken together, flowBin and flowType-DP form a powerful platform for high-throughput analysis of multitube flow cytometry data.

Multitube flow cytometry data is commonly used for leukemia diagnosis, and I have applied flowBin and flowType-DP to three retrospective acute myeloid leukemia data sets. I have demonstrated that flowBin can be used to distinguish normal from leukemic cell types, to find cell types associated with a particular genotype, and to survey the immunophenotypic landscape of a patient cohort. In the process, I have presented a more detailed survey of the immunophenotypes associated with NPM1 mutations in AML, shown the neoplastic immunophenotypic landscape of AML, and associated immunophenotype with a comprehensive survey of AML genetic lesions.

Secondly, I have I have contributed significantly to developing BAIT, a tool for detecting sister chromatid exchanges in strand-seq data. This lays the foundations for detecting translocations and other genomic rearrangements at the single cell level, which

may have profound impacts for researching drug resistance and relapse in AML. BAIT also enables the placing of unbridged contigs in late-build genomes, and I present the placing of half the remaining unplaced contigs in the current build of the mouse genome. Mice are an important model species for AML research,[144] and improvements to the quality of the mouse genome will aid their usage for this purpose.

Following from BAIT, I took the lead in developing contiBAIT, an R package for *de novo* assembling genomes with unbridged gaps using strand-seq. There are numerous model organisms with genomes consisting only of large numbers of unbridged, unanchored contigs. Many of these organisms are likely to be of use in cancer research. For example, the naked mole rat (*Heterocephalus glaber*), has unusually high cancer resistance,[145] but a genome currently only assembled into 4,230 unplaced contigs. ContiBAIT, with strand-seq, can be used to cheaply assemble these genomes into chromosomes, and to create preliminary ordering of those contigs along the chromosomes, for relatively little sequencing cost.

8.2 Reiteration of Problem Statement

Although the high-throughput methods of flow cytometry and strand-seq have produced large quantities of data that may facilitate life-saving research into AML treatment, the bioinformatics tools to analyze this data have been limited. Prior to this thesis work, tools for recombining the multitube flow cytometry data typical in AML diagnosis were few and produced imputation errors, while existing downstream methods were poorly suited for the high dimensionality of the resulting data. Tools for automatically analysing strand-seq were non-existent.

By developing flowBin and improving flowType, I have created tools to be able to accurately recombine multitube AML flow cytometry data, and further characterise that data in spite of its high-dimensional nature. By developing BAIT and contiBAIT, I have created tools to analyse strand-seq data, with applications in AML of measuring genomic instability, detecting subclonal genomic rearrangements, and improving the

assemblies of cancer model organism genomes. These tools will facilitate much-needed research using these data towards understanding AML biology and hence improving treatment outcomes.

8.3 Future Directions

8.3.1 FlowType-DP

A possible further refinement to FlowType-DP might include heuristic search of the cell type space. P-values, area under the curve, or other outcome metrics could be used as optimality criteria. This could enable much deeper exploration of the cell type space than flowType-DP's breadth-first strategy. However, such a search method may be susceptible to convergence at local optima (and thus miss phenotypes which a more comprehensive strategy would have found). Also, since the search is biased towards phenotypes with higher P-values or better classification scores, the search could be susceptible to overfitting, and in a statistical context, care would need to be taken to ensure that an appropriate multiple test correction was developed and applied.

Another possible refinement to flowType analysis would be the development of an appropriate multiple test correction for the comprehensive case. Existing methods, e.g. Bonferonni-Holm[146] and Benjamini-Hochbergi[147], assume independence between variables. However, the phenotypes produced by flowType are related hierarchically, and tend to be highly interdependent. Indeed, in some cases, adding a particular gate to a phenotype may have no effect whatsoever (for example, CD34+ cells are rarely also CD20+), so that some phenotypes are completely redundant. A multiple test correction which accounts for this feature of flowType data could substantially improve the statistical power of analyses incorporating flowType.

8.3.2 FlowBin

Future enhancements to the flowBin could include support for user-specified binning using the gating data structures within the flowCore package.[20] This would by extension support importing bin definitions from other software saved in Gating-ML format.[148] Another useful addition would be the option to support callback functions for computing the expression of bins. This would enable users to implement alternative methods for thresholding based on negative controls, such as Overton's cumulative subtraction

of histograms[149], Bagwell's super enhanced Dmax subtraction[150], Lampariello's ratio analysis of empirical cumulative density functions[151], or the more recent use of F-measure [152].

8.3.3 Applications of FlowBin to AML

In AML, blast count is typically quite high (at least 20% of bone marrow), but varies substantially and may be as high as 90%. This acts as a source of noise in flowBin-flowType analyses. The detection of abnormal cell types (and filtering out of normal) could be used to reduce that noise. However, in the other AML data sets I analysed, there were no normal patients available to use for this purpose. So a future direction would be to determine whether the FlowCAP2 AML data set (which is publicly available in FlowRepository) could be used as a training set for other data sets.

For the analysis associating NPM1 with phenotypes, the next step was to apply the same methods to a more comprehensive genotyping effort, which I did. For that genotyping effort, overall survival information is available, and it could be useful both for future prognostic purposes and to understanding the biology of AML to perform survival analysis. Both the NMF clusters, individual cell types, and combinations of these with recurrent gene mutations could be examined for survival differences and/or correlation. Another future analysis to be performed on that data set would be a comparison of the flow cytometry NMF clusters with NMF clustering of the same patients in terms of both their mRNA and miRNA expression (data for which is available). This could help to elucidate more of the biology underlying the genotype-phenotype correlations I observed. Lastly, in recent years software has become available for inferring clonal prevalence of genotypes, such as PyClone.[153] The clonal prevalence could then be compared with immunophenotype prevalences to attempt to infer the relationships between immunophenotypic variation within AML and genotypic variations at the single-cell level.

8.3.4 BAIT

The ability to detect sister chromatid exchanges (and similar discontinuities in strand inheritance) has many potential applications. One of the most important applications to AML diagnosis is the ability to detect genomic rearrangements, which appear as SCEs.[6] Since strand-seq is single cell, it could be possible to use it as a cheap, sensitive and automated method for detecting subclonal translocations and inversions, an important next step for AML research. There will be some challenges to this, such as the problem that some rearrangements would be masked in some cells by chance. Once per-cell predictions of rearrangements have been made, they could be used similarly to FISH to infer the clonal phylogeny of the tumour [154].

8.3.5 contiBAIT

My collaborator on BAIT and contiBAIT, Mark Hills, has sequenced strand-seq libraries from several organisms with early-build genomes, including zebrafish (*Danio rerio*), pig (*Sus scrofa*), ferret (*Mustela putorius*), Tasmanian devil (*Sarcophilus harrisii*) with accompanying facial tumour, and platypus (*Ornithorhynchus anatinus*). Mark is in the process of applying contiBAIT to this data, in order to improve the quality of the genomes for these organisms. In the future, strand-seq and contiBAIT will likely become one of a suite of low-cost tools complimenting and enhancing the results of whole-genome shotgun sequencing efforts.

While contiBAIT's current anchoring approach performs extremely well, the ordering component could be improved. A procedure of filtering more stringently and only attempting to order the higher-quality contigs could help. It may also be possible to estimate the background noise of the individual libraries, as BAIT does[6]; although this requires complete chromosomes, it could be adapted to work with larger contigs.

The ordering algorithm itself could also stand to be improved. Applying the Concorde framework[138] may produce more optimal travelling salesman problem solutions. Furthermore, the shortest Hamiltonian path / travelling salesman problem formulation

itself has some drawbacks, in that it does not account for some biological information, such as impossible strand state transitions, and detectable SCEs within contigs. A metaheuristic approach (such as a genetic algorithm) could be developed instead, with a customised objective function accounting for these factors. Although genetic algorithms (GAs) in their original formulation are notoriously poor at solving ordering problems due to becoming stuck in local sub-optima,[155] a formulation incorporating local heuristic search in place of the mutation operator has been used to solve TSPs quickly and with solutions equally optimal to benchmarks.[155] Incorporating this strategy with a more intelligent objective function could dramatically improve the ability of contiBAIT to correctly order contigs.

Bibliography

[1] K. O'Neill, N. Aghaeepour, J. Špidlen, and R. Brinkman, "Flow Cytometry Bioinformatics," *PLoS Comput Biol*, vol. 9, p. e1003365, Dec. 2013.

[2] K. O'Neill, A. Jalali, N. Aghaeepour, H. Hoos, and R. R. Brinkman, "Enhanced flowType/RchyOptimyx: A Bioconductor pipeline for discovery in high-dimensional cytometry data," *Bioinformatics*, vol. 30, pp. 1329–30, Jan. 2014.

[3] N. Aghaeepour, P. K. Chattopadhyay, A. Ganesan, K. O'Neill, H. Zare, A. Jalali, H. H. Hoos, M. Roederer, and R. R. Brinkman, "Early immunologic correlates of HIV protection can be identified from computational analysis of complex multivariate T-cell flow cytometry assays," *Bioinformatics*, vol. 28, no. 7, 2012.

[4] N. Aghaeepour, A. Jalali, K. O'Neill, P. K. Chattopadhyay, M. Roederer, H. H. Hoos, and R. R. Brinkman, "RchyOptimyx: Cellular hierarchy optimization for flow cytometry," *Cytometry Part A*, vol. 81, no. 12, pp. n/a–n/a, 2012.

[5] N. Aghaeepour, G. Finak, T. F. Consortium, T. D. Consortium, H. Hoos, T. R. Mosmann, R. Brinkman, R. Gottardo, and R. H. Scheuermann, "Critical assessment of automated flow cytometry data analysis techniques," *Nature Methods*, vol. 10, pp. 445–445, May 2013.

[6] M. Hills, K. O'Neill, E. Falconer, R. Brinkman, and P. M. Lansdorp, "BAIT: Organizing genomes and mapping rearrangements in single cells," *Genome Medicine*, vol. 5, p. 82, Sept. 2013.

[7] M. Roederer, "How many events is enough? Are you positive?," *Cytometry Part A*, vol. 73A, pp. 384–385, May 2008.

[8] B. D. Hedley and M. Keeney, "Technical issues: flow cytometry and rare event analysis," *International Journal of Laboratory Hematology*, vol. 35, pp. 344–350, June 2013.

[9] B. Brando, D. Barnett, G. Janossy, F. Mandy, B. Autran, G. Rothe, B. Scarpati, G. D'Avanzo, J.-L. D'Hautcourt, R. Lenkei, G. Schmitz, A. Kunkl, R. Chianese, S. Papa, and J. W. Gratama, "Cytofluorometric methods for assessing absolute numbers of cell subsets in blood," *Cytometry*, vol. 42, no. 6, pp. 327–346, 2000.

[10] C. S. Ferreira-Facio, C. Milito, V. Botafogo, M. Fontana, L. S. Thiago, E. Oliveira, A. S. da Rocha-Filho, F. Werneck, D. N. Forny, S. Dekermacher, A. P. de Azambuja, S. E. Ferman, P. A. S. de Faria, M. G. P. Land, A. Orfao, and E. S. Costa, "Contribution of Multiparameter Flow Cytometry Immunophenotyping to the Diagnostic Screening and Classification of Pediatric Cancer," *PLoS ONE*, vol. 8, p. e55534, Mar. 2013.

[11] D. Wu, B. L. Wood, and J. R. Fromm, "Flow Cytometry for Non-Hodgkin and Classical Hodgkin Lymphoma," *Methods in Molecular Biology*, vol. 971, pp. 27–47, Jan. 2013.

[12] Y. Wang, F. Hammes, K. De Roy, W. Verstraete, and N. Boon, "Past, present and future applications of flow cytometry in aquatic microbiology," *Trends in Biotechnology*, vol. 28, pp. 416–424, Aug. 2010.

[13] L. A. Johnson, J. P. Flook, M. V. Look, and D. Pinkel, "Flow sorting of X and Y chromosome-bearing spermatozoa into two populations," *Gamete research*, vol. 16, pp. 1–9, Jan. 1987.

[14] G. M. Baerlocher, I. Vulto, G. de Jong, and P. M. Lansdorp, "Flow cytometry and FISH to measure the average length of telomeres (flow FISH)," *Nature Protocols*, vol. 1, pp. 2365–2376, Dec. 2006.

[15] P. K. Chattopadhyay, C. M. Hogerkorp, and M. Roederer, "A chromatic explosion: the development and future of multiparameter flow cytometry.," *Immunology*, vol. 125, no. 4, p. 441, 2008.

[16] G. K. Behbehani, S. C. Bendall, M. R. Clutter, W. J. Fantl, and G. P. Nolan, "Single Cell Mass Cytometry Adapted to Measurements of the Cell Cycle," *Cytometry. Part A : the journal of the International Society for Analytical Cytology*, vol. 81, pp. 552–566, July 2012.

[17] A. K. White, M. VanInsberghe, O. I. Petriv, M. Hamidi, D. Sikorski, M. A. Marra, J. Piret, S. Aparicio, and C. L. Hansen, "High-throughput microfluidic single-cell RT-qPCR," *Proceedings of the National Academy of Sciences*, vol. 108, Aug. 2011.

[18] M. Roederer, "Compensation in Flow Cytometry," in *Current Protocols in Cytometry*, John Wiley and Sons, Inc., 2001.

[19] C. B. Bagwell and E. G. Adams, "Fluorescence Spectral Overlap Compensation for Any Number of Flow Cytometry Parameters," *Annals of the New York Academy of Sciences*, vol. 677, no. 1, pp. 167–184, 1993.

[20] F. Hahne, N. LeMeur, R. R. Brinkman, B. Ellis, P. Haaland, D. Sarkar, J. Spidlen, E. Strain, and R. Gentleman, "flowCore: a Bioconductor package for high throughput flow cytometry," *BMC Bioinformatics*, vol. 10, p. 106, Apr. 2009.

[21] H. M. Shapiro, *Practical flow cytometry*. Wiley-Liss New York, 2003.

[22] D. R. Parks, M. Roederer, and W. A. Moore, “A new “Logicle” display method avoids deceptive effects of logarithmic scaling for low signals and compensated data,” *Cytometry Part A*, vol. 69A, no. 6, pp. 541–551, 2006.

[23] G. Finak, J.-M. Perez, A. Weng, and R. Gottardo, “Optimizing transformations for automated, high throughput analysis of flow cytometry data,” *BMC Bioinformatics*, vol. 11, p. 546, Nov. 2010.

[24] W. A. Moore and D. R. Parks, “Update for the logicle data scale including operational code implementations,” *Cytometry Part A*, vol. 81A, no. 4, pp. 273–277, 2012.

[25] C. B. Bagwell, “Hyperlog—A flexible log-like transform for negative, zero, and positive valued data,” *Cytometry Part A*, vol. 64A, no. 1, pp. 34–42, 2005.

[26] K. Lo, R. R. Brinkman, and R. Gottardo, “Automated gating of flow cytometry data via robust model-based clustering.,” *Cytometry.Part A : the journal of the International Society for Analytical Cytology*, vol. 73, pp. 321–332, Apr. 2008.

[27] Y. Qian, Y. Liu, J. Campbell, E. Thomson, Y. M. Kong, and R. H. Scheuermann, “FCSTrans: An open source software system for FCS file conversion and data transformation,” *Cytometry Part A*, vol. 81A, no. 5, pp. 353–356, 2012.

[28] N. Le Meur, A. Rossini, M. Gasparetto, C. Smith, R. R. Brinkman, and R. Gentleman, “Data quality assessment of ungated flow cytometry data in high throughput experiments,” *Cytometry Part A*, vol. 71, no. 6, pp. 393–403, 2007.

[29] W. T. Rogers, A. R. Moser, H. A. Holyst, A. Bantly, E. R. Mohler III, G. Scangas, and J. S. Moore, “Cytometric fingerprinting: Quantitative characterization of multivariate distributions,” *Cytometry Part A*, vol. 73, no. 5, pp. 430–441, 2008.

[30] F. Hahne, A. H. Khodabakhshi, A. Bashashati, C. J. Wong, R. D. Gascoyne, A. P. Weng, V. Seyfert-Margolis, K. Bourcier, A. Asare, T. Lumley, and Others, “Per-channel basis normalization methods for flow cytometry data,” *Cytometry Part A*, vol. 77, no. 2, pp. 121–131, 2010.

[31] K. Hornik, “A CLUE for CLUster Ensembles,” *Journal of Statistical Software*, vol. 14, no. 12, pp. 1–25, 2005.

[32] E. Lugli, M. Roederer, and A. Cossarizza, “Data analysis in flow cytometry: the future just started,” *Cytometry. Part A: the journal of the International Society for Analytical Cytology*, vol. 77, pp. 705–713, July 2010.

[33] S. C. Bendall and G. P. Nolan, “From single cells to deep phenotypes in cancer,” *Nature Biotechnology*, vol. 30, no. 7, pp. 639–647, 2012.

[34] H. T. Maecker and J. Trotter, “Flow cytometry controls, instrument setup, and the determination of positivity,” *Cytometry Part A*, vol. 69A, no. 9, pp. 1037–1042, 2006.

[35] L. K. McNeil, L. Price, C. M. Britten, M. Jaimes, H. Maecker, K. Odunsi, J. Matsuzaki, J. S. Staats, J. Thorpe, J. Yuan, and S. Janetzki, "A harmonized approach to intracellular cytokine staining gating: Results from an international multiconsortia proficiency panel conducted by the Cancer Immunotherapy Consortium (CIC/CRI)," *Cytometry Part A*, vol. 83A, pp. 728–738, Aug. 2013.

[36] S. Bendall, E. Simonds, and P. Qiu, "Single-cell mass cytometry of differential immune and drug responses across a human hematopoietic continuum," *Science . . .*, vol. 332, no. 6030, p. 687, 2011.

[37] P. F. Virgo and G. J. Gibbs, "Flow cytometry in clinical pathology," *Annals of Clinical Biochemistry*, vol. 49, pp. 17–28, Jan. 2012.

[38] E. S. da Costa, C. E. Pedreira, S. Barrena, Q. Lecrevisse, J. Flores, S. Quijano, J. Almeida, M. del Carmen García-Macias, S. Bottcher, J. J. M. Van Dongen, and Others, "Automated pattern-guided principal component analysis vs expert-based immunophenotypic classification of B-cell chronic lymphoproliferative disorders: a step forward in the standardization of clinical immunophenotyping," *Leukemia*, 2010.

[39] P. Qiu, E. F. Simonds, S. C. Bendall, K. D. Gibbs Jr, R. V. Bruggner, M. D. Linderman, K. Sachs, G. P. Nolan, and S. K. Plevritis, "Extracting a cellular hierarchy from high-dimensional cytometry data with SPADE," *Nature biotechnology*, 2011.

[40] S. Pyne, X. Hu, K. Wang, E. Rossin, T.-I. Lin, L. M. Maier, C. Baecher-Allan, G. J. McLachlan, P. Tamayo, D. A. Hafler, P. L. D. Jager, and J. P. Mesirov, "Automated high-dimensional flow cytometric data analysis," *Proc Natl Acad Sci U S A*, 2009.

[41] Y. Qian, C. Wei, F. Eun-Hyung Lee, J. Campbell, J. Halliley, J. A. Lee, J. Cai, Y. M. Kong, E. Sadat, E. Thomson, P. Dunn, A. C. Seegmiller, N. J. Karandikar, C. M. Tipton, T. Mosmann, I. n. Sanz, and R. H. Scheuermann, "Elucidation of seventeen human peripheral blood B-cell subsets and quantification of the tetanus response using a density-based method for the automated identification of cell populations in multidimensional flow cytometry data," *Cytometry Part B: Clinical Cytometry*, vol. 78B, no. S1, pp. S69–S82, 2010.

[42] H. Zare, P. Shooshtari, A. Gupta, and R. R. Brinkman, "Data reduction for spectral clustering to analyze high throughput flow cytometry data," *BMC bioinformatics*, vol. 11, no. 1, p. 403, 2010.

[43] N. Aghaeepour, R. Nikolic, H. H. Hoos, and R. R. Brinkman, "Rapid Cell Population Identification in Flow Cytometry Data," *submitted to Cytometry Part A*, 2010.

[44] Y. Ge and S. C. Sealfon, "flowPeaks: a fast unsupervised clustering for flow cytometry data via K-means and density peak finding," *Bioinformatics*, vol. 28, pp. 2052–2058, Aug. 2012.

[45] M. Roederer, W. Moore, A. Treister, R. R. Hardy, and L. A. Herzenberg, "Probability binning comparison: a metric for quantitating multivariate distribution differences," *Cytometry Part A*, vol. 45, no. 1, pp. 47–55, 2001.

[46] M. Roederer and R. R. Hardy, "Frequency difference gating: A multivariate method for identifying subsets that differ between samples," *Cytometry*, vol. 45, no. 1, pp. 56–64, 2001.

[47] A. Cron, C. Gouttefangeas, J. Frelinger, L. Lin, S. K. Singh, C. M. Britten, M. J. P. Welters, S. H. van der Burg, M. West, and C. Chan, "Hierarchical Modeling for Rare Event Detection and Cell Subset Alignment across Flow Cytometry Samples," *PLoS Comput Biol*, vol. 9, p. e1003130, July 2013.

[48] "FlowJo - vX Manual - Boolean Gates," 2013.

[49] H. Zare, A. Bashashati, R. Kridel, N. Aghaeepour, G. Haffari, J. M. Connors, R. D. Gascoyne, A. Gupta, R. R. Brinkman, and A. P. Weng, "Automated Analysis of Multidimensional Flow Cytometry Data Improves Diagnostic Accuracy Between Mantle Cell Lymphoma and Small Lymphocytic Lymphoma," *American Journal of Clinical Pathology*, vol. 137, pp. 75–85, Jan. 2012.

[50] P. Qiu, "Inferring Phenotypic Properties from Single-Cell Characteristics," *PLoS ONE*, vol. 7, p. e37038, May 2012.

[51] B. Bodenmiller, E. R. Zunder, R. Finck, T. J. Chen, E. S. Savig, R. V. Bruggner, E. F. Simonds, S. C. Bendall, K. Sachs, P. O. Krutzik, and G. P. Nolan, "Multiplexed mass cytometry profiling of cellular states perturbed by small-molecule regulators," *Nature Biotechnology*, vol. 30, pp. 858–867, Sept. 2012.

[52] A. Bashashati, N. A. Johnson, A. H. Khodabakhshi, M. D. Whiteside, H. Zare, D. W. Scott, K. Lo, R. Gottardo, F. S. Brinkman, J. M. Connors, G. W. Slack, R. D. Gascoyne, A. P. Weng, and R. R. Brinkman, "B Cells With High Side Scatter Parameter by Flow Cytometry Correlate With Inferior Survival in Diffuse Large B-Cell Lymphoma," *American journal of clinical pathology*, vol. 137, pp. 805–814, May 2012.

[53] E. J. Freireich and M. J. Keating, *The Merck manual of diagnosis and therapy*, ch. 142: Leuke. Merck Research Laboratories Whitehouse Station (NJ), 2006.

[54] S. H. Swerdlow, E. Campo, N. L. Harris, E. S. Jaffe, S. A. Pileri, H. Stein, J. Thiele, and J. W. Vardiman, eds., *WHO classification of tumours of haematopoietic and lymphoid tissues*. International Agency for Research on Cancer, 2008.

[55] E. Estey and H. Döhner, "Acute myeloid leukaemia," *The Lancet*, vol. 368, pp. 1894–1907, Dec. 2006.

[56] D. Grimwade, H. Walker, G. Harrison, F. Oliver, S. Chatters, C. J. Harrison, K. Wheatley, A. K. Burnett, and A. H. Goldstone, "The predictive value of hierarchical cytogenetic classification in older adults with acute myeloid leukemia (AML): analysis of 1065 patients entered into the United Kingdom Medical Research Council AML11 trial," *Blood*, vol. 98, no. 5, p. 1312, 2001.

[57] B. B. Zeisig, A. G. Kulasekararaj, G. J. Mufti, and C. W. Eric So, "SnapShot: Acute Myeloid Leukemia," *Cancer Cell*, vol. 22, pp. 698–698.e1, Nov. 2012.

[58] R. F. Schlenk, K. Döhner, J. Krauter, S. Fröhling, A. Corbacioglu, L. Bullinger, M. Habdank, D. Späth, M. Morgan, A. Benner, B. Schlegelberger, G. Heil, A. Ganser, and H. Döhner, "Mutations and Treatment Outcome in Cytogenetically Normal Acute Myeloid Leukemia," *New England Journal of Medicine*, vol. 358, no. 18, pp. 1909–1918, 2008.

[59] J. M. Bennett, D. Catovsky, M. T. Daniel, G. Flandrin, D. A. Galton, H. R. Gralnick, and C. Sultan, "Proposals for the classification of the acute leukaemias. French-American-British (FAB) co-operative group.," *Br J Haematol*, vol. 33, pp. 451–458, Aug. 1976.

[60] K. A. Foon, R. F. Todd, and Others, "Immunologic classification of leukemia and lymphoma," *Blood*, vol. 68, no. 1, p. 1, 1986.

[61] C. D. Jennings and K. A. Foon, "Recent advances in flow cytometry: application to the diagnosis of hematologic malignancy," *Blood*, vol. 90, no. 8, p. 2863, 1997.

[62] M. C. Bene, G. Castoldi, W. Knapp, W. D. Ludwig, E. Matutes, A. Orfao, and V. VAN'T, "Proposals for the immunological classification of acute leukemias," *Leukemia*, vol. 9, no. 10, pp. 1783–1786, 1995.

[63] E. S. Jaffe, N. L. Harris, H. Stein, and J. W. Vardiman, eds., *WHO classification of tumours of haematopoietic and lymphoid tissues.* International Agency for Research on Cancer, 2001.

[64] S. Grisendi and P. P. Pandolfi, "NPM mutations in acute myelogenous leukemia," *New England Journal of Medicine*, vol. 352, no. 3, p. 291, 2005.

[65] R. G. W. Verhaak, C. S. Goudswaard, W. van Putten, M. A. Bijl, M. A. Sanders, W. Hugens, A. G. Uitterlinden, C. A. J. Erpelinck, R. Delwel, B. Lowenberg, and Others, "Mutations in nucleophosmin (NPM1) in acute myeloid leukemia (AML): association with other gene abnormalities and previously established gene expression signatures and their favorable prognostic significance," *Blood*, vol. 106, no. 12, p. 3747, 2005.

[66] K. Dohner, R. F. Schlenk, M. Habdank, C. Scholl, F. G. Rucker, A. Corbacioglu, L. Bullinger, S. Frohling, and H. Dohner, "Mutant nucleophosmin (NPM1) predicts favorable prognosis in younger adults with acute myeloid leukemia and normal cytogenetics: interaction with other gene mutations," *Blood*, vol. 106, no. 12, p. 3740, 2005.

[67] S. Schnittger, C. Schoch, W. Kern, C. Mecucci, C. Tschulik, M. F. Martelli, T. Haferlach, W. Hiddemann, and B. Falini, "Nucleophosmin gene mutations are predictors of favorable prognosis in acute myelogenous leukemia with a normal karyotype," *Blood*, vol. 106, no. 12, p. 3733, 2005.

[68] P. A. Ho, T. A. Alonzo, R. B. Gerbing, J. Pollard, D. L. Stirewalt, C. Hurwitz, N. A. Heerema, B. Hirsch, S. C. Raimondi, B. Lange, J. L. Franklin, J. P. Radich, and S. Meshinchi, "Prevalence and prognostic implications of CEBPA mutations in pediatric acute myeloid leukemia (AML): a report from the Children's Oncology Group," *Blood*, vol. 113, pp. 6558–6566, June 2009.

[69] P. D. Kottaridis, R. E. Gale, M. E. Frew, G. Harrison, S. E. Langabeer, A. A. Belton, H. Walker, K. Wheatley, D. T. Bowen, A. K. Burnett, and Others, "The presence of a FLT3 internal tandem duplication in patients with acute myeloid leukemia (AML) adds important prognostic information to cytogenetic risk group and response to the first cycle of chemotherapy: analysis of 854 patients from the United King," *Blood*, vol. 98, no. 6, p. 1752, 2001.

[70] T. J. Ley, E. R. Mardis, L. Ding, B. Fulton, M. D. McLellan, K. Chen, D. Dooling, B. H. Dunford-Shore, S. McGrath, M. Hickenbotham, L. Cook, R. Abbott, D. E. Larson, D. C. Koboldt, C. Pohl, S. Smith, A. Hawkins, S. Abbott, D. Locke, L. W. Hillier, T. Miner, L. Fulton, V. Magrini, T. Wylie, J. Glasscock, J. Conyers, N. Sander, X. Shi, J. R. Osborne, P. Minx, D. Gordon, A. Chinwalla, Y. Zhao, R. E. Ries, J. E. Payton, P. Westervelt, M. H. Tomasson, M. Watson, J. Baty, J. Ivanovich, S. Heath, W. D. Shannon, R. Nagarajan, M. J. Walter, D. C. Link, T. A. Graubert, J. F. DiPersio, and R. K. Wilson, "DNA sequencing of a cytogenetically normal acute myeloid leukemia genome," *Nature*, vol. 456, pp. 66–72, Nov. 2008.

[71] E. R. Mardis, L. Ding, D. J. Dooling, D. E. Larson, M. D. McLellan, K. Chen, D. C. Koboldt, R. S. Fulton, K. D. Delehaunty, S. D. McGrath, L. A. Fulton, D. P. Locke, V. J. Magrini, R. M. Abbott, T. L. Vickery, J. S. Reed, J. S. Robinson, T. Wylie, S. M. Smith, L. Carmichael, J. M. Eldred, C. C. Harris, J. Walker, J. B. Peck, F. Du, A. F. Dukes, G. E. Sanderson, A. M. Brummett, E. Clark, J. F. McMichael, R. J. Meyer, J. K. Schindler, C. S. Pohl, J. W. Wallis, X. Shi, L. Lin, H. Schmidt, Y. Tang, C. Haipek, M. E. Wiechert, J. V. Ivy, J. Kalicki, G. Elliott, R. E. Ries, J. E. Payton, P. Westervelt, M. H. Tomasson, M. A. Watson, J. Baty, S. Heath, W. D. Shannon, R. Nagarajan, D. C. Link, M. J. Walter, T. A. Graubert, J. F. DiPersio, R. K. Wilson, and T. J. Ley, "Recurring Mutations Found by Sequencing an Acute Myeloid Leukemia Genome," *New England Journal of Medicine*, vol. 361, no. 11, pp. 1058–1066, 2009.

[72] T. Ley, C. Miller, L. Ding, B. Raphael, A. Mungall, A. Robertson, K. Hoadley, T. Triche, P. Laird, J. Baty, and Others, "Genomic and Epigenomic Landscapes of Adult De Novo Acute Myeloid Leukemia," *New England Journal of Medicine*, vol. 368, no. 22, pp. 2059–2074, 2013.

[73] F. E. Craig and K. A. Foon, "Flow cytometric immunophenotyping for hematologic neoplasms," *Blood*, vol. 111, no. 8, p. 3941, 2008.

[74] B. L. Wood, M. Arroz, D. Barnett, J. DiGiuseppe, B. Greig, S. J. Kussick, T. Oldaker, M. Shenkin, E. Stone, and P. Wallace, "2006 Bethesda International Consensus recommendations on the immunophenotypic analysis of hematolymphoid neoplasia by flow cytometry: Optimal reagents and reporting for the flow cytometric

diagnosis of hematopoietic neoplasia," *Cytometry Part B: Clinical Cytometry*, vol. 72, no. S1, pp. S14—-S22, 2007.

[75] B. H. Davis, J. T. Holden, M. C. Bene, M. J. Borowitz, R. C. Braylan, D. Cornfield, W. Gorczyca, R. Lee, R. Maiese, A. Orfao, and Others, "2006 Bethesda International Consensus recommendations on the flow cytometric immunophenotypic analysis of hematolymphoid neoplasia: Medical indications," *Cytometry Part B: Clinical Cytometry*, vol. 72, no. S1, pp. S5—-S13, 2007.

[76] J. J. M. van Dongen, L. Lhermitte, S. Böttcher, J. Almeida, V. H. J. van der Velden, J. Flores-Montero, A. Rawstron, V. Asnafi, Q. Lécrevisse, P. Lucio, E. Mejstrikova, T. Szczepański, T. Kalina, R. de Tute, M. Brüggemann, L. Sedek, M. Cullen, A. W. Langerak, A. Mendonça, E. Macintyre, M. Martin-Ayuso, O. Hrusak, M. B. Vidriales, and A. Orfao, "EuroFlow antibody panels for standardized n-dimensional flow cytometric immunophenotyping of normal, reactive and malignant leukocytes," *Leukemia*, vol. 26, no. 9, pp. 1908–1975, 2012.

[77] M. J. Borowitz, K. L. Guenther, K. E. Shults, and G. T. Stelzer, "Immunophenotyping of acute leukemia by flow cytometric analysis: use of CD45 and right-angle light scatter to gate on leukemic blasts in three-color analysis," *American journal of clinical pathology*, vol. 100, no. 5, pp. 534–540, 1993.

[78] R. O. Rainer, L. Hodges, and G. T. Seltzer, "CD 45 gating correlates with bone marrow differential," *Cytometry*, vol. 22, no. 2, pp. 139–145, 1995.

[79] F. Lacombe, F. Durrieu, A. Briais, P. Dumain, F. Belloc, E. Bascans, J. Reiffers, M. R. Boisseau, and P. Bernard, "Flow cytometry CD45 gating for immunophenotyping of acute myeloid leukemia," *Leukemia*, vol. 11, no. 11, pp. 1878–1886, 1997.

[80] C. E. Pedreira, E. S. Costa, S. Barrena, Q. Lecrevisse, J. Almeida, J. J. M. van Dongen, A. Orfao, and Others, "Generation of flow cytometry data files with a potentially infinite number of dimensions," *Cytometry Part A*, vol. 73, no. 9, pp. 834–846, 2008.

[81] G. Lee, W. Finn, and C. Scott, "Statistical file matching of flow cytometry data," *Journal of Biomedical Informatics*, 2011.

[82] E. Zamir, B. Geiger, N. Cohen, Z. Kam, and B. Z. Katz, "Resolving and classifying haematopoietic bone-marrow cell populations by multi-dimensional analysis of flow-cytometry data," *British journal of haematology*, vol. 129, no. 3, pp. 420–431, 2005.

[83] E. Falconer, M. Hills, U. Naumann, S. S. S. Poon, E. A. Chavez, A. D. Sanders, Y. Zhao, M. Hirst, and P. M. Lansdorp, "DNA template strand sequencing of single-cells maps genomic rearrangements at high resolution," *Nature Methods*, vol. 9, pp. 1107–1112, Nov. 2012.

[84] E. Falconer and P. M. Lansdorp, “Strand-seq: A unifying tool for studies of chromosome segregation,” *Seminars in Cell and Developmental Biology*, vol. 24, no. 8-9, pp. 643–652, 2013.

[85] P. M. Lansdorp, “Immortal Strands? Give Me a Break,” *Cell*, vol. 129, pp. 1244–1247, June 2007.

[86] J. Cairns, “Mutation selection and the natural history of cancer,” *Nature*, vol. 255, pp. 197–200, May 1975.

[87] E. Sonoda, M. S. Sasaki, C. Morrison, Y. Yamaguchi-Iwai, M. Takata, and S. Takeda, “Sister Chromatid Exchanges Are Mediated by Homologous Recombination in Vertebrate Cells,” *Molecular and Cellular Biology*, vol. 19, pp. 5166–5169, July 1999.

[88] R. G. Langlois, W. L. Bigbee, R. H. Jensen, and J. German, “Evidence for increased in vivo mutation and somatic recombination in Bloom’s syndrome.,” *Proceedings of the National Academy of Sciences of the United States of America*, vol. 86, pp. 670–674, Jan. 1989.

[89] R. A. Burrell, N. McGranahan, J. Bartek, and C. Swanton, “The causes and consequences of genetic heterogeneity in cancer evolution,” *Nature*, vol. 501, pp. 338–345, Sept. 2013.

[90] M. E. Pretorius, H. k. Waehre, V. M. Abeler, B. Davidson, L. Vlatkovic, R. A. Lothe, K.-E. Giercksky, and H. v. E. Danielsen, “Large scale genomic instability as an additive prognostic marker in early prostate cancer,” *Cellular oncology: the official journal of the International Society for Cellular Oncology*, vol. 31, no. 4, pp. 251–259, 2009.

[91] A. Vincent-Salomon, V. Benhamo, E. Gravier, G. Rigaill, N. Gruel, S. Robin, Y. de Rycke, O. Mariani, G. Pierron, D. Gentien, F. Reyal, P. Cottu, A. Fourquet, R. Rouzier, X. Sastre-Garau, and O. Delattre, “Genomic Instability: A Stronger Prognostic Marker Than Proliferation for Early Stage Luminal Breast Carcinomas,” *PLoS ONE*, vol. 8, p. e76496, Oct. 2013.

[92] R. K. R. Mettu, Y.-W. Wan, J. K. Habermann, T. Ried, and N. L. Guo, “A 12-Gene Genomic Instability Signature Predicts Clinical Outcomes in Multiple Cancer Types,” *The International journal of biological markers*, vol. 25, no. 4, pp. 219–228, 2010.

[93] J. S. Wainscoat and M. F. Fey, “Assessment of clonality in human tumors: a review,” *Cancer research*, vol. 50, pp. 1355–1360, Mar. 1990.

[94] J. Brewin, G. Horn, and T. Chevassut, “Genomic Landscapes and Clonality of De Novo AML,” *New England Journal of Medicine*, vol. 369, no. 15, pp. 1472–1473, 2013.

[95] L. Ding, T. J. Ley, D. E. Larson, C. A. Miller, D. C. Koboldt, J. S. Welch, J. K. Ritchey, M. A. Young, T. Lamprecht, M. D. McLellan, J. F. McMichael, J. W. Wallis, C. Lu, D. Shen, C. C. Harris, D. J. Dooling, R. S. Fulton, L. L. Fulton, K. Chen, H. Schmidt, J. Kalicki-Veizer, V. J. Magrini, L. Cook, S. D. McGrath, T. L. Vickery, M. C. Wendl, S. Heath, M. A. Watson, D. C. Link, M. H. Tomasson, W. D. Shannon, J. E. Payton, S. Kulkarni, P. Westervelt, M. J. Walter, T. A. Graubert, E. R. Mardis, R. K. Wilson, and J. F. DiPersio, "Clonal evolution in relapsed acute myeloid leukaemia revealed by whole-genome sequencing," *Nature*, vol. 481, pp. 506–510, Jan. 2012.

[96] M. Greaves and C. C. Maley, "Clonal Evolution in Cancer," *Nature*, vol. 481, pp. 306–313, Jan. 2012.

[97] S. Fröhling, S. Skelin, C. Liebisch, C. Scholl, R. F. Schlenk, H. Döhner, and K. Döhner, "Comparison of Cytogenetic and Molecular Cytogenetic Detection of Chromosome Abnormalities in 240 Consecutive Adult Patients With Acute Myeloid Leukemia," *Journal of Clinical Oncology*, vol. 20, pp. 2480–2485, May 2002.

[98] S. C. Bendall, G. P. Nolan, M. Roederer, and P. K. Chattopadhyay, "A deep profiler's guide to cytometry," *Trends in Immunology*, vol. 33, pp. 323–332, July 2012.

[99] F. Villanova, P. D. Meglio, M. Inokumad, N. Aghaeepour, E. Perucha, J. Mollon, L. Nomura, M. Hernandez-Fuentes, A. Copeh, T. Prevosti, S. Heck, V. Maino, G. Lord, R. R. Brinkman, and and Frank O. Nestle, "Integration of Lyoplate based Flow Cytometry and Computational Analysis for Standardized Immunological Biomarker Discovery," *PLoS ONE*, vol. 8, no. 7, 2013.

[100] F. Craig, R. Brinkman, E. Ten, and N. Aghaeepour, "Computational Analysis Optimizes the Flow Cytometric Evaluation for Lymphoma," *Cytometry Part B - Clinical Cytometry*, vol. Digital pr, 2013.

[101] D. Eppstein, "Finding the k shortest paths," *SIAM Journal on computing*, vol. 28, no. 2, pp. 652–673, 1998.

[102] J. P. Vial and F. Lacombe, "Immunophenotyping of acute leukemia: Utility of CD45 for blast cell identification," *Methods in cell biology*, vol. 64, pp. 343–358, 2001.

[103] C. E. Pedreira, E. S. Costa, M. E. Arroyo, J. Almeida, and A. Orfao, "A multidimensional classification approach for the automated analysis of flow cytometry data," *IEEE Transactions on Biomedical Engineering*, vol. 55, no. 3, pp. 1155–1162, 2008.

[104] B. M. Bolstad, R. A. Irizarry, M. Astrand, and T. P. Speed, "A comparison of normalization methods for high density oligonucleotide array data based on variance and bias," *Bioinformatics*, vol. 19, no. 2, p. 185, 2003.

[105] G. K. Smyth, "limma: Linear Models for Microarray Data," in *Bioinformatics and Computational Biology Solutions Using R and Bioconductor* (R. Gentleman, V. J. Carey, W. Huber, R. A. Irizarry, and S. Dudoit, eds.), Statistics for Biology and Health, pp. 397–420, Springer New York, Jan. 2005.

[106] E. M. A. Hensor, L. Hunt, R. Parmar, A. Burska, P. Emery, and F. Ponchel, "A1.33 Predicting the evolution of inflammatory arthritis in ACPA-positive individuals: can T-cell subsets help?," *Annals of the Rheumatic Diseases*, vol. 73, pp. A14–A14, Mar. 2014.

[107] N. T. Colburn, K. J. M. Zaal, F. Wang, and R. S. Tuan, "A role for γ/δ T cells in a mouse model of fracture healing," *Arthritis and Rheumatism*, vol. 60, pp. 1694–1703, June 2009.

[108] S. M. Garrido, F. R. Appelbaum, C. L. Willman, and D. E. Banker, "Acute myeloid leukemia cells are protected from spontaneous and drug-induced apoptosis by direct contact with a human bone marrow stromal cell line (HS-5)," *Experimental Hematology*, vol. 29, pp. 448–457, Apr. 2001.

[109] Y. Parel and C. Chizzolini, "CD4+ CD8+ double positive (DP) T cells in health and disease," *Autoimmunity Reviews*, vol. 3, pp. 215–220, Mar. 2004.

[110] Y. Chen, X. S. Zhou, and T. Huang, "One-class SVM for learning in image retrieval," in *International Conference on Image Processing*, vol. 1, pp. 34–37 vol.1, 2001.

[111] C. Thiede, S. Koch, E. Creutzig, C. Steudel, T. Illmer, M. Schaich, and G. Ehninger, "Prevalence and prognostic impact of NPM1 mutations in 1485 adult patients with acute myeloid leukemia (AML)," *Blood*, vol. 107, no. 10, p. 4011, 2006.

[112] M. Syampurnawati, E. Tatsumi, B. Ardianto, M. Takenokuchi, Y. Nakamachi, S. Kawano, S. Kumagai, K. Saigo, T. Matsui, T. Takahashi, and Others, "DR negativity is a distinctive feature of M1/M2 AML cases with NPM1 mutation," *Leukemia research*, vol. 32, no. 7, pp. 1141–1143, 2008.

[113] B. Dalal, S. Mansoor, M. Manna, S. Pi, G. Sauro, and D. Hogge, "Detection of CD34, TdT, CD56, CD2, CD4, and CD14 by Flow Cytometry Is Associated With¡ i¿ NPM1¡/i¿ and¡ i¿ FLT3¡/i¿ Mutation Status in Cytogenetically Normal Acute Myeloid Leukemia," *Clinical Lymphoma Myeloma and Leukemia*, 2012.

[114] A. Karatzoglou, A. Smola, K. Hornik, and A. Zeileis, "kernlab – An {S4} Package for Kernel Methods in {R}," *Journal of Statistical Software*, vol. 11, no. 9, pp. 1–20, 2004.

[115] R. Gaujoux and C. Seoighe, "A flexible R package for nonnegative matrix factorization," *BMC Bioinformatics*, vol. 11, p. 367, July 2010.

[116] L. Breiman, "Bagging Predictors," *Machine Learning*, vol. 24, pp. 123–140, Aug. 1996.

[117] L. I. Shlush, S. Zandi, A. Mitchell, W. C. Chen, J. M. Brandwein, V. Gupta, J. A. Kennedy, A. D. Schimmer, A. C. Schuh, K. W. Yee, J. L. McLeod, M. Doedens, J. J. F. Medeiros, R. Marke, H. J. Kim, K. Lee, J. D. McPherson, T. J. Hudson, T. H. Pan-Leukemia Gene Panel Consortium, A. M. K. Brown, Q. M. Trinh, L. D. Stein, M. D. Minden, J. C. Y. Wang, and J. E. Dick, "Identification of pre-leukaemic haematopoietic stem cells in acute leukaemia," *Nature*, vol. 506, pp. 328–333, Feb. 2014.

[118] L. M. Kelly and D. G. Gilliland, "Genetics of Myeloid Leukemias," *Annual Review of Genomics and Human Genetics*, vol. 3, no. 1, pp. 179–198, 2002.

[119] Y. Ishikawa, H. Kiyoi, A. Tsujimura, S. Miyawaki, Y. Miyazaki, K. Kuriyama, M. Tomonaga, and T. Naoe, "Comprehensive analysis of cooperative gene mutations between class I and class II in de novo acute myeloid leukemia," *European Journal of Haematology*, vol. 83, pp. 90–98, Aug. 2009.

[120] Z. Li, X. Cai, C.-L. Cai, J. Wang, W. Zhang, B. E. Petersen, F.-C. Yang, and M. Xu, "Deletion of Tet2 in mice leads to dysregulated hematopoietic stem cells and subsequent development of myeloid malignancies," *Blood*, vol. 118, pp. 4509–4518, Oct. 2011.

[121] F. Albano, A. Mestice, A. Pannunzio, F. Lanza, B. Martino, D. Pastore, F. Ferrara, P. Carluccio, F. Nobile, G. Castoldi, V. Liso, and G. Specchia, "The biological characteristics of CD34+ CD2+ adult acute promyelocytic leukemia and the CD34 CD2 hypergranular (M3) and microgranular (M3v) phenotypes," *Haematologica*, vol. 91, pp. 311–316, Jan. 2006.

[122] B. Falini, K. Macijewski, T. Weiss, U. Bacher, S. Schnittger, W. Kern, A. Kohlmann, H. Klein, M. Vignetti, A. Piciocchi, and Others, "Multilineage dysplasia has no impact on biologic, clinicopathologic, and prognostic features of AML with mutated nucleophosmin (NPM1)," *Blood*, vol. 115, no. 18, pp. 3776–3786, 2010.

[123] A. B. Olshen, E. S. Venkatraman, R. Lucito, and M. Wigler, "Circular binary segmentation for the analysis of array-based DNA copy number data," *Biostatistics*, vol. 5, pp. 557–572, Oct. 2004.

[124] V. E. Seshan and A. Olshen, *DNAcopy: DNA copy number data analysis*, 2013.

[125] N. Nagarajan, C. Cook, M. D. Bonaventura, H. Ge, A. Richards, K. A. Bishop-Lilly, R. DeSalle, T. D. Read, and M. Pop, "Finishing genomes with limited resources: lessons from an ensemble of microbial genomes," *BMC Genomics*, vol. 11, p. 242, Apr. 2010.

[126] W. J. Kent, C. W. Sugnet, T. S. Furey, K. M. Roskin, T. H. Pringle, A. M. Zahler, and D. Haussler, "The Human Genome Browser at UCSC," *Genome Research*, vol. 12, pp. 996–1006, June 2002.

[127] L. R. Meyer, A. S. Zweig, A. S. Hinrichs, D. Karolchik, R. M. Kuhn, M. Wong, C. A. Sloan, K. R. Rosenbloom, G. Roe, B. Rhead, B. J. Raney, A. Pohl, V. S. Malladi, C. H. Li, B. T. Lee, K. Learned, V. Kirkup, F. Hsu, S. Heitner, R. A. Harte, M. Haeussler, L. Guruvadoo, M. Goldman, B. M. Giardine, P. A. Fujita, T. R. Dreszer, M. Diekhans, M. S. Cline, H. Clawson, G. P. Barber, D. Haussler, and W. J. Kent, "The UCSC Genome Browser database: extensions and updates 2013," *Nucleic Acids Research*, vol. 41, pp. D64–D69, Jan. 2013.

[128] G. R. Sutherland, E. Baker, and R. S. Seshadri, "Heritable fragile sites on human chromosomes. V. A new class of fragile site requiring BrdU for expression.," *American Journal of Human Genetics*, vol. 32, pp. 542–548, July 1980.

[129] W. Chen, V. Kalscheuer, A. Tzschach, C. Menzel, R. Ullmann, M. H. Schulz, F. Erdogan, N. Li, Z. Kijas, G. Arkesteijn, I. L. Pajares, M. Goetz-Sothmann, U. Heinrich, I. Rost, A. Dufke, U. Grasshoff, B. Glaeser, M. Vingron, and H. H. Ropers, "Mapping translocation breakpoints by next-generation sequencing," *Genome Research*, vol. 18, pp. 1143–1149, July 2008.

[130] M. Hunt, C. Newbold, M. Berriman, and T. D. Otto, "A comprehensive evaluation of assembly scaffolding tools," *Genome Biology*, vol. 15, p. R42, Mar. 2014.

[131] W. Bateson, E. R. Saunders, and R. C. Punnett, "Report II. Experimental studies in the physiology of heredity," tech. rep., Royal Society, 1904.

[132] N. G. Copeland and N. A. Jenkins, "Development and applications of a molecular genetic linkage map of the mouse genome," *Trends in Genetics*, vol. 7, pp. 113–118, Apr. 1991.

[133] Z. Huang, "A Fast Clustering Algorithm to Cluster Very Large Categorical Data Sets in Data Mining," in *Research Issues on Data Mining and Knowledge Discovery*, pp. 1–8, 1997.

[134] Z. S. Qin, "Clustering microarray gene expression data using weighted Chinese restaurant process," *Bioinformatics*, vol. 22, pp. 1988–1997, Aug. 2006.

[135] *BOOST C++ Libraries*, 2014.

[136] Intel, "Intel SSE4 Programming Reference," Tech. Rep. July, Intel, 2007.

[137] M. Maechler, P. Rousseeuw, A. Struyf, M. Hubert, and K. Hornik, "cluster: Cluster Analysis Basics and Extensions," 2014.

[138] D. Applegate and R. Bixby, "On the Solution of Traveling Salesman Problems," *Documenta Mathematica*, vol. Proceeding, pp. 645–656, 1998.

[139] G. A. Croes, "A Method for Solving Traveling-Salesman Problems," *Operations Research*, vol. 6, pp. 791–812, Dec. 1958.

[140] D. Haussler, S. J. O'Brien, O. A. Ryder, F. K. Barker, M. Clamp, A. J. Crawford, R. Hanner, O. Hanotte, W. E. Johnson, J. A. McGuire, and Others, "Genome 10K: a proposal to obtain whole-genome sequence for 10,000 vertebrate species.," *The Journal of heredity*, vol. 100, no. 6, pp. 659–674, 2008.

[141] E. T. Lam, A. Hastie, C. Lin, D. Ehrlich, S. K. Das, M. D. Austin, P. Deshpande, H. Cao, N. Nagarajan, M. Xiao, and P.-Y. Kwok, "Genome mapping on nanochannel arrays for structural variation analysis and sequence assembly," *Nature Biotechnology*, vol. 30, pp. 771–776, Aug. 2012.

[142] J. N. Burton, A. Adey, R. P. Patwardhan, R. Qiu, J. O. Kitzman, and J. Shendure, "Chromosome-scale scaffolding of de novo genome assemblies based on chromatin interactions," *Nature Biotechnology*, vol. 31, pp. 1119–1125, Dec. 2013.

[143] M. Mascher, G. J. Muehlbauer, D. S. Rokhsar, J. Chapman, J. Schmutz, K. Barry, M. Muñoz Amatriaín, T. J. Close, R. P. Wise, A. H. Schulman, A. Himmelbach, K. F. Mayer, U. Scholz, J. A. Poland, N. Stein, and R. Waugh, "Anchoring and ordering NGS contig assemblies by population sequencing (POPSEQ)," *The Plant Journal*, vol. 76, pp. 718–727, Nov. 2013.

[144] N. McCarthy, "Mouse Models: Closer than you think," *Nature Reviews Cancer*, vol. 9, pp. 382–383, June 2009.

[145] E. B. Kim, X. Fang, A. A. Fushan, Z. Huang, A. V. Lobanov, L. Han, S. M. Marino, X. Sun, A. A. Turanov, P. Yang, S. H. Yim, X. Zhao, M. V. Kasaikina, N. Stoletzki, C. Peng, P. Polak, Z. Xiong, A. Kiezun, Y. Zhu, Y. Chen, G. V. Kryukov, Q. Zhang, L. Peshkin, L. Yang, R. T. Bronson, R. Buffenstein, B. Wang, C. Han, Q. Li, L. Chen, W. Zhao, S. R. Sunyaev, T. J. Park, G. Zhang, J. Wang, and V. N. Gladyshev, "Genome sequencing reveals insights into physiology and longevity of the naked mole rat," *Nature*, vol. 479, pp. 223–227, Nov. 2011.

[146] S. Holm, "A simple sequentially rejective multiple test procedure," *Scandinavian Journal of Statistics*, vol. 6, no. 2, pp. 65–70, 1979.

[147] Y. Benjamini and Y. Hochberg, "Controlling the false discovery rate: a practical and powerful approach to multiple testing," *Journal of the Royal Statistical Society. Series B (Methodological)*, vol. 57, no. 1, pp. 289–300, 1995.

[148] J. Spidlen, R. C. Leif, W. Moore, M. Roederer, International Society for the Advancement of Cytometry Data Standards Task Force, and R. R. Brinkman, "Gating-ML: XML-based gating descriptions in flow cytometry," *Cytometry. Part A: the journal of the International Society for Analytical Cytology*, vol. 73A, pp. 1151–1157, Dec. 2008.

[149] W. R. Overton, "Modified histogram subtraction technique for analysis of flow cytometry data," *Cytometry*, vol. 9, no. 6, pp. 619–626, 1988.

[150] B. Bagwell, "A journey through flow cytometric immunofluorescence analyses—Finding accurate and robust algorithms that estimate positive fraction distributions," *Clinical Immunology Newsletter*, vol. 16, pp. 33–37, Mar. 1996.

[151] F. Lampariello, "Evaluation of the number of positive cells from flow cytometric immunoassays by mathematical modeling of cellular autofluorescence," *Cytometry*, vol. 15, no. 4, pp. 294–301, 1994.

[152] A. J. Richards, J. Staats, J. Enzor, K. McKinnon, J. Frelinger, T. N. Denny, K. J. Weinhold, and C. Chan, "Setting objective thresholds for rare event detection in flow cytometry.," *Journal of immunological methods*, Apr. 2014.

[153] A. Roth, J. Khattra, D. Yap, A. Wan, E. Laks, J. Biele, G. Ha, S. Aparicio, A. Bouchard-Côté, and S. P. Shah, "PyClone: statistical inference of clonal population structure in cancer," *Nature Methods*, vol. 11, pp. 396–398, Apr. 2014.

[154] S. A. Chowdhury, S. E. Shackney, K. Heselmeyer-Haddad, T. Ried, A. A. Schäffer, and R. Schwartz, "Phylogenetic analysis of multiprobe fluorescence in situ hybridization data from tumor cell populations," *Bioinformatics*, vol. 29, pp. i189–i198, July 2013.

[155] S. Sabba and S. Chikhi, "Integrating the best 2-Opt method to enhance the genetic algorithm execution time in solving the traveler salesman problem," in *Complex Systems and Dependability*, pp. 195–208, Springer, 2012.
